# Unilateral ureteral obstruction causes gut microbial dysbiosis and metabolome disorders contributing to tubulointerstitial fibrosis

**DOI:** 10.1038/s12276-019-0234-2

**Published:** 2019-03-27

**Authors:** Lin Chen, Dan-Qian Chen, Jing-Ru Liu, Jun Zhang, Nosratola D. Vaziri, Shougang Zhuang, Hua Chen, Ya-Long Feng, Yan Guo, Ying-Yong Zhao

**Affiliations:** 1 School of Pharmacy, Faculty of Life Science and Medicine, Northwest University, No. 229 Taibai North Road, 710069 Xi’an, Shaanxi China; 2 Division of Nephrology and Hypertension, School of Medicine, University of California Irvine, Irvine, CA 92897 USA; 3 Department of Internal Medicine, University of New Mexico, Comprehensive Cancer Center, Albuquerque, NM 87131 USA; 4 Department of Nephrology, Shanghai East Hospital, Tongji University School of Medicine, No. 150 Jimo Road, 200120 Shanghai, China

**Keywords:** Kidney diseases, Microbial genetics

## Abstract

Chronic kidney disease (CKD) increases the risk and prevalence of cardiovascular disease (CVD) morbidity and mortality. Recent studies have revealed marked changes in the composition of the microbiome and the metabolome and their potential influence in renal disease and CVD via the accumulation of microbial-derived uremic toxins. However, the effect of unilateral ureteral obstruction (UUO) on the gut microbiome and circulating metabolites is unknown. Male Sprague-Dawley rats were randomized to UUO and sham-operated control groups. Renal histology, colonic microbiota, and plasma metabolites were examined two weeks later. We employed 16S rRNA sequence and untargeted metabolomic analyses to explore the changes in colonic microbiota and plasma metabolites and their relationship with tubulointerstitial fibrosis (TIF). The UUO rats exhibited tubular atrophy and dilatation, interstitial fibrosis and inflammatory cell infiltration in the obstructed kidney. UUO rats showed significant colonic enrichment and depletion of genera. Significant differences were identified in 219 plasma metabolites involved in lipid, amino acid, and bile acid metabolism, which were consistent with gut microbiota-related metabolism. Interestingly, tryptophan and its metabolites kynurenine, 5-hydroxytryptophan and 5-hydroxytryptamine levels, which were linked with TIF, correlated with nine specific genera. Plasma tryptophan level was positively correlated with *Clostridium IV,* *Turicibacter*, *Pseudomonas* and *Lactobacillales*, and negatively correlated with *Oscillibacter*, *Blautia*, and *Intestinimonas*, which possess the genes encoding tryptophan synthase (K16187), indoleamine 2,3-dioxygenase (K00463) and tryptophan 2,3-dioxygenase (K00453) and their corresponding enzymes (EC:1.13.11.52 and EC:1.13.11.11) that exacerbate TIF. In conclusion, UUO results in profound changes in the gut microbiome and circulating metabolites, events that contribute to the pathogenesis of inflammation and TIF.

## Chronic kidney disease: The contribution of gut bacteria

An imbalance in gut bacteria contributes to kidney tissue scarring and declined kidney function. An international study led by Ying-Yong Zhao at Northwest University, Xi’an, China, analyzed the composition of the gut microbes in a rat model of chronic renal injury. They found that urinary tract obstruction was associated with changes in gut microbe composition and altered gut microbe-related metabolism of lipids, amino acids and bile acid. Lower levels of the essential amino acid tryptophan in plasma were linked to the deterioration of renal function. Treatment with ergone, a compound extracted from medicinal mushrooms, restored the levels of plasma tryptophan as well as the expression of proteins involved in maintaining the intestinal barrier. These findings suggest that restoring the function of the intestinal barrier could prevent kidney damage due to microbial imbalance in the gut.

## Introduction

Tubulointerstitial fibrosis (TIF) is the final manifestation of chronic kidney disease (CKD). Patients with progressive CKD inevitably reach end-stage renal disease (ESRD) requiring renal replacement therapies including dialysis and transplantation^1–4^. Although CKD is caused by a wide variety of diseases, such as diabetes, glomerulonephritis, interstitial nephritis, obstructive nephropathy, and polycystic kidney disease, etc., TIF represents the final common pathway of progression to ESRD^5^. TIF is characterized by excessive accumulation and deposition of extracellular matrix (ECM) components. This pathologic process usually originates from underlying diseases and is mediated by a number of independent and overlapping cellular and molecular pathways, such as monocyte/macrophage infiltration, fibroblast activation, epithelial-to-mesenchymal transition (EMT) and cellular apoptosis, as well as the activation of signaling molecules such as transforming growth factor beta (TGF-β) and angiotensin II^6–9^. To illuminate the specific pathways involved in the pathogenesis of TIF, many experimental studies have been conducted using animal models.

Recently, the imbalance of the gut microbiota has been identified as a new contributing factor to the pathogenesis of chronic inflammation and oxidative stress, which are the major mediators of cardiovascular disease and other complications in CKD patients^10^. The human gut is the natural habitat of ~10–100 trillion microorganisms, consisting of 500–1000 different species, harboring the largest and most diverse microbial ecosystem in the human body^11,12^. More than five phyla have been identified, among which *Bacteroidetes* and *Firmicutes* dominate the flora in the human gut. Various clinical and animal studies have demonstrated that gut microbiota interacts with the host in a mutually beneficial coexistence (symbiotic relationship) and performs an important role in both health maintenance and disease pathogenesis^13,14^. The normal gut microbiome forms a natural defense force and affects the well-being of the host by modulating physiology, nutrition, metabolism, and the immune system^13,14^. The normal gut microbiome protects the host against pathogenic microorganisms and chronic inflammation by protecting the intestinal epithelial barrier structure and function and promoting epithelial repair following injury^15,16^.

Recently, a few studies have demonstrated significant dysbiosis of the composition and function of gut microbiota in both patients and animals with CKD^15^. The dysbiosis contributes to an elevation of the gut microbiota-derived uremic toxins such as indoxyl sulfate (IS), p-cresyl sulfate (p-CS), and trimethylamine N-oxide (TMAO), and increased intestinal permeability by the expansion of urease-possessing bacteria^10,17–20^. The disruption of the intestinal barrier causes translocation of uremic toxins and microbial products into the systemic circulation. Thus, this process activates innate immunity, resulting in systemic inflammation, which plays a central role in CKD and the pathogenesis of CKD-associated complications^10,15^. Recently, new therapeutic strategies have emerged to modulate the gut microbiota imbalance in CKD patients by using probiotics, prebiotics, or symbiotics^10,15^. Because the pathogenesis of TIF is extremely complicated and our knowledge regarding the relationship of gut microbiota and related metabolites is limited, further investigations are needed to elucidate the influence of the gut-microbiota–kidney axis on TIF by the interaction of the gut microbiome and endogenous metabolites. The unilateral ureteral obstruction (UUO) model is a well-established model of experimental chronic renal injury characterized by TIF. Both acute and chronic ureteral obstruction occurs in various clinical settings. Individuals with chronic obstructive uropathy may progress to ESRD. To dissect the effect of urinary tract obstruction from that of uremia on the microbiome and metabolome we used UUO, which, by promoting contralateral kidney hypertrophy, minimizes the accumulation of uremic toxins/metabolites. Using the combination of microbiome and metabolome analyses, we investigated the direct links between the gut microbiome and metabolome and their impact on TIF in UUO rats.

## Materials and methods

### Animals and the experimental protocol

Male Sprague-Dawley rats (180–200 g) were purchased from the Central Animal Breeding House of Xi’an Jiaotong University (Xi’an, Shaanxi, China). Animal protocols were approved by Northwest University institutional animal care and use committee. The rats (*n* = 12) were randomized into two groups: sham and UUO groups. The UUO procedure was performed as previously described^21,22^. *Polyporus umbellatus* (PU, a medicinal fungus) and ergone (ERG, a steroid compound from PU) treatment were further used to investigate their effect on plasma metabolites, gut microbiota and TIF. Rats used in the additional experiment were randomized into the sham, UUO, UUO + ergone, and UUO + PU groups. The n-hexane extract of PU (184 mg/kg) and ergone (10 mg/kg) was given to UUO rats by intragastric administration. All animals were provided access to water and food ad libitum. The sham-operated, UUO and treated rats were sacrificed at week 2. Feces, blood, luminal contents of the colon, colon tissues, and kidney tissues were collected and processed for 16S rRNA sequence, untargeted and targeted metabolomic, histological, and western blot analyses.

### Histological analysis

Periodic acid–Schiff (PAS) staining and Masson’s Trichrome staining were carried out and glomerulosclerosis and tubulointerstitial damage were assessed as previously described^23^. Immunohistochemistry (IHC) for tissues was carried out as previously described. After immunohistochemical analysis, Image-Pro Plus software (Version 6.0, Media Cybernetics, Inc., Rockville, MD, USA) was used to analyze the optical density of the images as described previously^24,25^. Immunofluorescence staining for tissues or cells was performed as described previously^26^. The sections were fixed with 4% paraformaldehyde. After they were blocked with normal goat serum, sections were stained with primary antibodies. Image analysis was done by using Image-Pro Plus 6.0 software.

### Western blot analysis

The following primary antibodies were employed (dilution): ZO1 (1:1000, ab96587, Abcam, USA), occludin (1:1000, ab167161, Abcam, USA), claudin-1 (1:1000, ab15098, Abcam, USA), IκBα (1:2000, 4812, Cell Signaling Technology, USA), p-IκBα (1:2000, 2859, Cell Signaling Technology, USA), NF-κB p65 (1:1000, ab16502, Abcam, USA), monocyte chemotactic protein-1 (MCP-1, 1:1000, ab7202; Abcam, USA), cyclooxygenase-2 (COX-2, 1:1000, ab62331; Abcam, USA), Keap1 (1:1000, ab139729, Abcam, USA), Nrf2 (1:1000, ab31163, Abcam, USA), heme oxygenase 1 (HO-1, 1:2000, ab68477, Abcam, USA), catalase (1:1000, ab52477, Abcam, USA), NAD(P)H quinone dehydrogenase 1 (NQO1, 1:1000, ab28947, Abcam, USA), α smooth muscle actin (α-SMA, 1:300, ab7817, Abcam, USA), collagen I (1:5000, ab34710, Abcam, USA), and fibronectin (1:1000, ab2413, Abcam, USA). Western blot analysis was performed as previously described^24,26,27^. Blots were obtained with ECL reagent and protein concentrations were normalized by actin expression. Specific bands indicating target proteins were analyzed using ImageJ 1.48 v software.

### High throughput sequencing of colon lumen DNA

The colon lumen of each sample was dissected with a sterilized blade and stored at −80 °C for DNA extraction. Genomic DNA was extracted using an E.Z.N.A.^®^ Soil DNA Kit (Omega Bio-tek, Norcross, GA, U.S.) according to the manufacturer’s protocols. The concentrations and purity of the resultant DNA were determined using a NanoDrop (NanoDrop ND-2000, USA) and stored at –80 °C for further analysis.

The 16S rRNA gene was amplified by PCR with primers 16S-F (5′-AGAGTTTGATYMTGGCTCAG-3′) and 16S-R (5′-TGCTGCCTCCCG TAGGAGT-3′) targeting the hypervariable V4–V5 region of the bacterial 16S rRNA gene. PCR reactions were performed in triplicate with Phusion^®^ High-Fidelity PCR Master Mix (New England Biolabs) using a 10-ng template DNA. PCR products were purified using the AxyPrep DNA Gel Extraction Kit (Axygen Biosciences, Union City, CA, U.S.) according to the manufacturer’s instructions and quantified using QuantiFluor™-ST (Promega, U.S.). The PCR products of different samples were mixed equally and were used to construct an Illumina Pair-End library using a Next^®^ Ultra™ DNA Library Prep Kit for Illumina (Nebraska, USA). Then, the amplicon library was paired-end sequenced (2 × 250) on an Illumina HiSeq 2500 platform (Illumina, San Diego, USA) according to the standard protocols.

### Processing of 16S rRNA gene sequences

Raw FASTQ files were demultiplexed using the barcode sequence with the exact barcode matching parameters and quality-filtered using Trimmomatic (version 0.36)^28^ with the following criteria: (i) bases off the start and end of a read below a threshold quality (score < 3) were removed; and (ii) the reads were truncated at any site receiving an average quality score < 5 over a 4 bp sliding window, discarding the truncated reads that were shorter than 100 bp. Paired-reads were merged using USEARCH FASTQ_merge pairs command (version 9.2.64, http://drive5.com/uparse/)^29^ with the default parameters. The operational units (OTUs) were clustered with a 97% similarity cutoff using USEARCH UPARSE^30^. The chimeric sequences were removed in the UPARSE pipeline. The phylogenetic affiliation of each 16S rRNA gene sequence was analyzed by using a USEARCH SINTAX algorithm^31^ against the RDP training set (version v16) 16S rRNA database using a confidence threshold of 0.8. The OTUs identified as mitochondrial or chloroplast rRNA sequences were discarded. The rarefaction analysis based on USEARCH α_diversity^32^ was conducted to reveal the diversity indices, including the richness, chao1, Simpson, and Shannon diversity indices. The β diversity analysis was performed using UniFrac metrics^33^ in the QIIME (version 1.9.1)^34^ pipeline.

### Sample preparation and UPLC-MS analysis for metabolomics

Plasma metabolite analyses were performed using an untargeted metabolomics UPLC-HDMS. The metabolomic procedures, including sample preparation, metabolite separation and detection, data preprocessing, and statistical analysis for metabolite identification, were performed following our previous protocols with minor modifications^35–37^.

### Statistics analysis

The statistical analyses were performed using R 2.15.0 and GraphPad Prism software v 5.0 (GraphPad Software, San Diego, CA, USA). PCA and OPLS-DA were performed using SIMCA-P software to cluster the sample plots across groups. Differential abundances of genera and metabolites were tested by one-way analysis of variance (ANOVA) and non-parametric tests, including the Wilcoxon rank-sum test and Mann–Whitney *U* test. Plasma metabolite intensities were then tested for their association with 16S levels using Spearman rank correlation. *P* values were corrected for multiple comparisons using the Benjamini–Hochberg false discovery rate (FDR). A corrected *P* value < 0.05 was considered statistically significant.

## Results

### Gut microbial structure in UUO rats

**Fig. 1:**
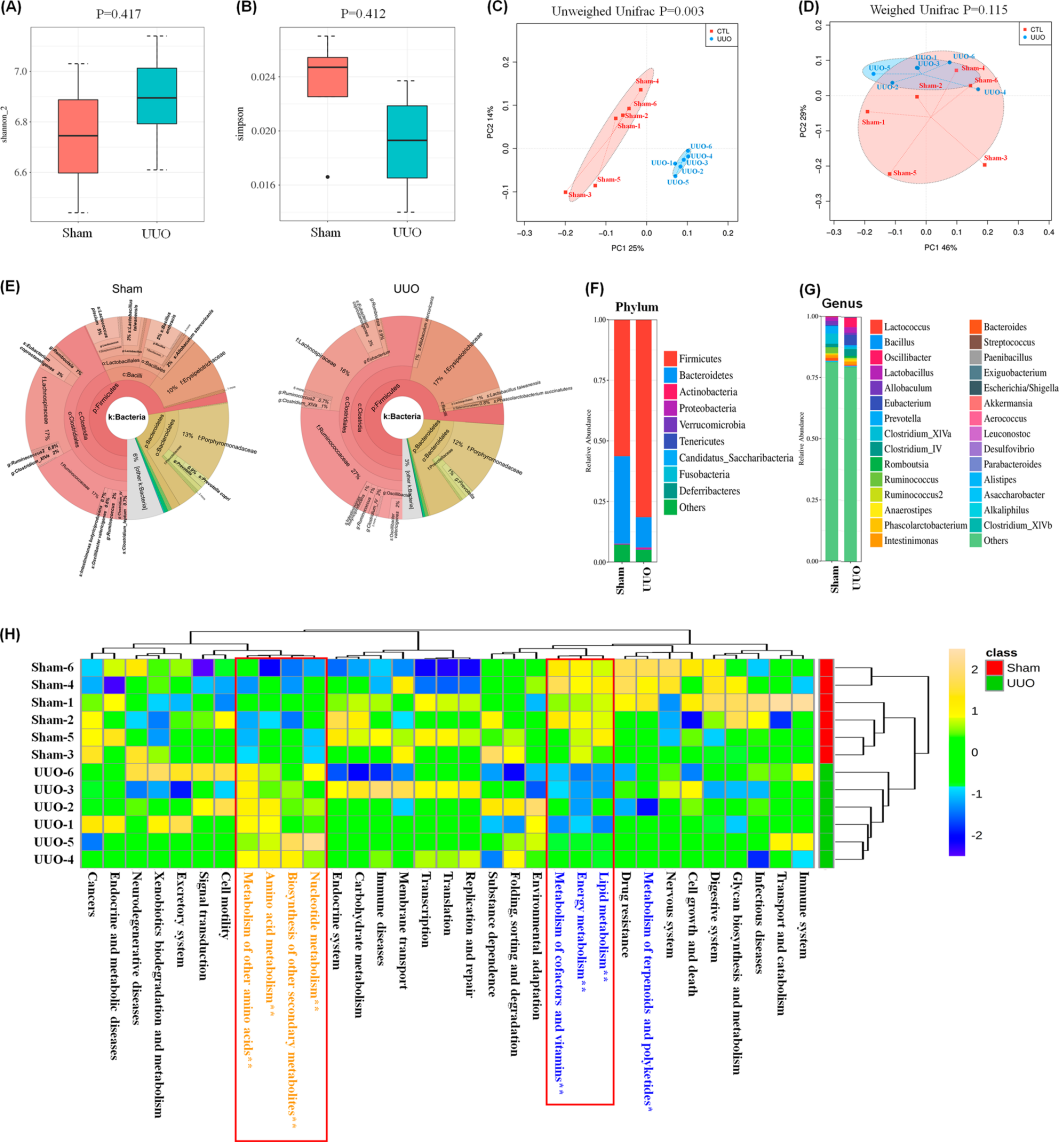
Altered gut microbiome and functional metabolic pathways in UUO rats. **a**, **b** Shannon_2 and Simpson of α-diversity index of colonic luminal content 16S rDNA sequencing data from UUO and sham rats after two weeks. The boxplots indicate the smallest and largest values, 25 and 75% quartiles, medians, and outliers. **c**, **d** Principal coordinates analysis of unweighted and weighted UniFrac distance based on 16S rDNA profiling (OTUs level) of colonic luminal content from sham and UUO rats. *P* values indicated differential clustering when assessed by ADONIS test. **e** Cladogram showing different abundant taxa between samples from sham and UUO rats using phylum to genus-level data. **f**, **g** Taxonomic distributions of bacteria from colonic luminal content 16S rDNA sequencing data at the phylum (top 10) and genus (top 30) levels between the UUO and sham groups. Wilcoxon rank-sum test was used to determine significance in α-diversity. **h** Modifications of the gut microbiota affect predicted functional metabolic pathways. Heatmap of metabolic pathways of the UUO and sham groups obtained from PICRUSt analysis of 16S rRNA sequencing data. Yellow and blue text highlights indicate upregulation and downregulation in the UUO rats versus the sham rats. Asterisks denote statistical significance between the sham and UUO groups (*n* = 6), ^*^P *<* 0.05, ^**^P *<* 0.01

16S rDNA sequence analysis was performed to determine the composition of microbiota contained in the colonic lumen of the study animals. After quality control, we obtained a total of 574,365 sequence reads, with an average number of 40,678 reads per sample (range 31,836–48,991). A total of 817 OTUs, with an average of 563 observed OTUs per sample, were identified from UUO and sham rats. The α-diversity indices were used to describe the ecological diversity of the gut microbiome. The Shannon_2 index values, reflecting both the species richness and evenness, were 6.53 ± 0.44 and 6.48 ± 0.39 in sham and UUO groups, respectively (Fig. 1a). The Simpson index reflecting community evenness was 0.034 ± 0.019 and 0.36 ± 0.018 in sham and UUO groups, respectively (Fig. 1b). According to the Wilcoxon rank-sum test, the α-diversity indices, including richness, Chao1, Shannon_2, Simpson, Dominance, and Equitability, were not significantly different between the UUO and sham groups (Table S1).

A significant difference was observed in β-diversity based on the unweighted (ADONIS) analysis (*P* = 0.003) but not the weighted (ADONIS) UniFrac analysis (*P* = 0.1155) between the UUO and sham groups (Fig. 1c, d). The PCoA plots of the unweighted UniFrac showed that the gut microbiota of UUO rats was clearly different from those of the sham group (Fig. 1c). The sham rats were spread more widely on the PCoA plot, indicating a diverse gut microbiota. In contrast, the data on UUO rats were clustered closely together. Therefore, these results indicated that UUO could significantly influence the structure and composition of the gut microbiome.

### Alteration of taxa and potential metabolic pathways in UUO rats

UUO altered the composition of the gut microbiome (Table S2). The different abundant taxa between samples from sham and UUO rats are presented in Fig. 1e using phylum to genus-level data. At the phyla level, all the sequences could be assigned to 12 phyla (Fig. 1f). Firmicutes and Bacteroidetes dominated the gut microbiome of both groups. Firmicutes were the most dominant gut microbiota, accounting for an average of 72.5% and 76.1% of the sequences in the sham and UUO rats, respectively. Bacteroidetes were the second dominant gut microbiota, accounting for an average of 20.3% and 19.7% sequences in the sham and UUO rats, respectively. Other phyla were detected at low levels (less than 3%). No significant difference was found in all phyla between the two groups. At the class level, the relative abundance showed that Clostridia (p_Firmicutes), Bacteroidia (p_Bacteroidetes) Erysipelotrichia, (p_Firmicutes), and Bacilli (p_Firmicutes) were predominant in both groups (Fig. 1e). Their relative abundances were not significantly different between the two groups except for Bacilli, which was significantly decreased in the UUO group. At the order level, only the relative abundance of Lactobacillales was significantly decreased in the UUO rats compared to the sham rats (Fig. 1e). At the genus level, an average of 66.7% and 72.3% of the sequences per sample could not be assigned to specific genera in the sham and UUO groups, respectively (Fig. 1g), which is consistent with the result of a previous study^38^. The rest of the sequences were assigned to 82 genera, in which *Lactobacillus, Eubacterium, Clostridium XlVa*, *Allobaculum*, and *Prevotella* were abundant in both groups (Fig. 1g).

### Effects of microbial modifications on the functional pathways

To understand the potential gene functions of the different gut microbiota, the functional composition of the bacterial metagenome using 16S rRNA data were predicted by using a PICRUSt analysis. KEGG orthology analysis identified 48 significantly altered pathways in the UUO rats (Fig. S1; Table S3). The significantly altered metabolic pathways included: sphingolipid metabolism, tryptophan, tyrosine, phenylalanine, valine and leucine biosynthesis and degradation, bile acid biosynthesis and secretion, fatty acids biosynthesis and degradation, purine metabolism, and the MAPK signaling pathway (Fig. S2). These metabolic pathways are associated with 273 enzymes, such as indoleamine 2,3-dioxygenase, tryptophan 2-monooxygenase, tryptophan synthase, kynurenine 3-monooxygenase, kynureninase, tryptophanase, tyrosine decarboxylase, butyrate kinase, glutamate synthase, isocitrate lyase, urease, tryptophan—tRNA ligase, and trimethylamine-N-oxide reductase (Figs. S3 and S4). Interestingly, 95 significantly altered enzymes in the UUO rats were associated with amino acid biosynthesis and metabolism (Fig. S3). Taken together, the altered microbial metabolic pathways and related enzymes involved 33 metabolic pathways catalogs (Fig. 1h). Interestingly, amino acid metabolism, nucleotide metabolism and biosynthesis of other secondary metabolites were significantly upregulated, whereas lipid metabolism, cofactor and vitamin metabolism, and energy metabolism were significantly downregulated in the UUO rats. Thus, changes in bacterial communities caused by UUO can potentially alter multiple microbial metabolic pathways.

**Fig. 2:**
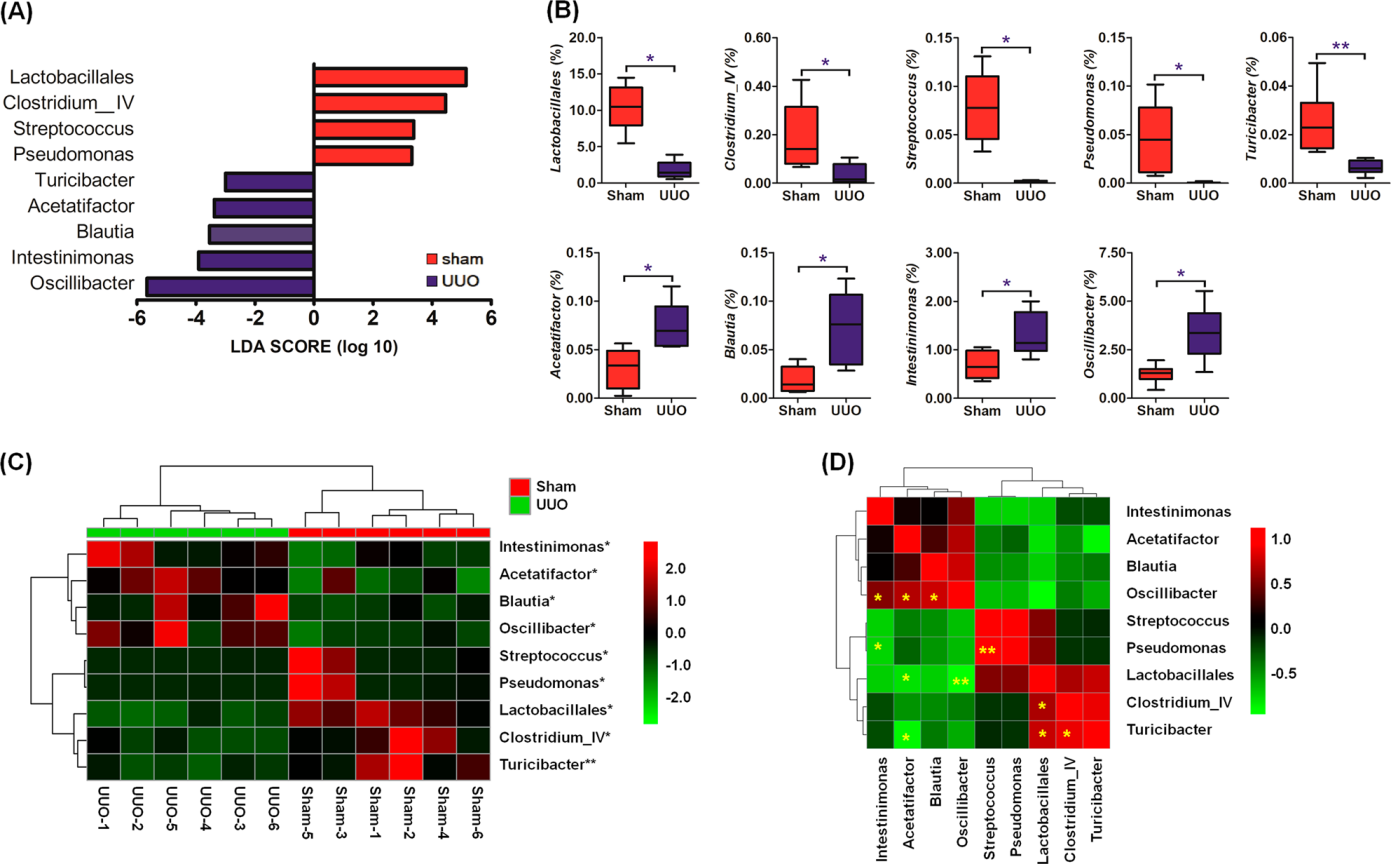
Significantly altered bacterial taxa in UUO rats. **a** LDA score of the significantly discriminant genera between the two groups (LDA score > 2.0, Wilcoxon rank-sum tests, *P* < 0.05). **b** Boxplots showing differences in the relative abundance of nine significantly discriminant taxa driving gut microbiome differences between the UUO and sham rats. Boxplots labeled with different asterisks indicated taxa abundances are significantly different between the UUO and sham rats. Single and double asterisks indicated *P* < 0.05 and *P* < 0.01 obtained by using Wilcoxon rank-sum tests, respectively. **c** Heatmap of nine significantly discriminant taxa in the sham and UUO rats, where rows represent bacterial taxa indicated. Columns represent colonic luminal content samples collected from the UUO and sham rats. Asterisks denote statistical significance between the sham and UUO groups (*n* = 6), ^*^P *<* 0.05, ^**^P *<* 0.01. **d** Correlations of nine significantly discriminant taxa in the UUO and sham rats by using Spearman correlation analysis; Asterisks denote statistical significance between bacterial taxa, ^*^P *<* 0.05, ^**^P *<* 0.01

Further, multivariate statistical analysis was applied to select significant bacterial taxa. Linear discriminant analysis (LDA) effect size (LEfSe) allowed us to identify taxa driving these differences and causing dysfunctions and altering metabolic pathways. As shown in Fig. 2a, b, UUO rats were mainly characterized by a higher abundance of *Oscillibacter*, *Intestinimonas*, *Blautia*, and *Acetatifactor* affiliated with Firmicutes (Wilcoxon rank-sum test), which are commonly found in other kidney diseases^39–41^, and whose genomes contain several fatty acid and amino acid modules. In contrast, the sham rats contained a higher abundance of genera affiliated with the Firmicutes, including *Clostridium IV* (*Eubacterium siraeum*), *Streptococcus* (*Streptococcus salivarius subsp salivarius*), and *Turicibacter* as well as Proteobacteria *Pseudomonas*, reflecting normal renal function. Overall, we identified nine taxa with significantly different abundances between the two groups (Fig. 2a). Heatmap analysis displayed that these taxa could significantly separate the UUO rats from the sham rats (Fig. 2c). Further Pearson correlation analysis indicated that several taxa were strongly correlated in the UUO rats (Fig. 2d). *Oscillibacter* was positively correlated with *Intestinimonas*, *Acetatifactor* and *Blautia* in UUO rats. Strong positive correlations were found between *Pseudomonas* with *Streptococcus*, *Clostridium IV* with *Lactobacillales*, and *Turicibacter* with *Lactobacillales* and *Clostridium IV* in UUO rats (Fig. 2d). In contrast, *Acetatifactor* showed significant negative correlations with *Lactobacillales* and *Turicibacter* in UUO rats. In addition, a strong negative correlation was found between *Pseudomonas* with *Intestinimonas* and *Oscillibacter* with *Lactobacillales* (Fig. 2d).

### Impact of the gut microbial modification on plasma metabolites

**Fig. 3:**
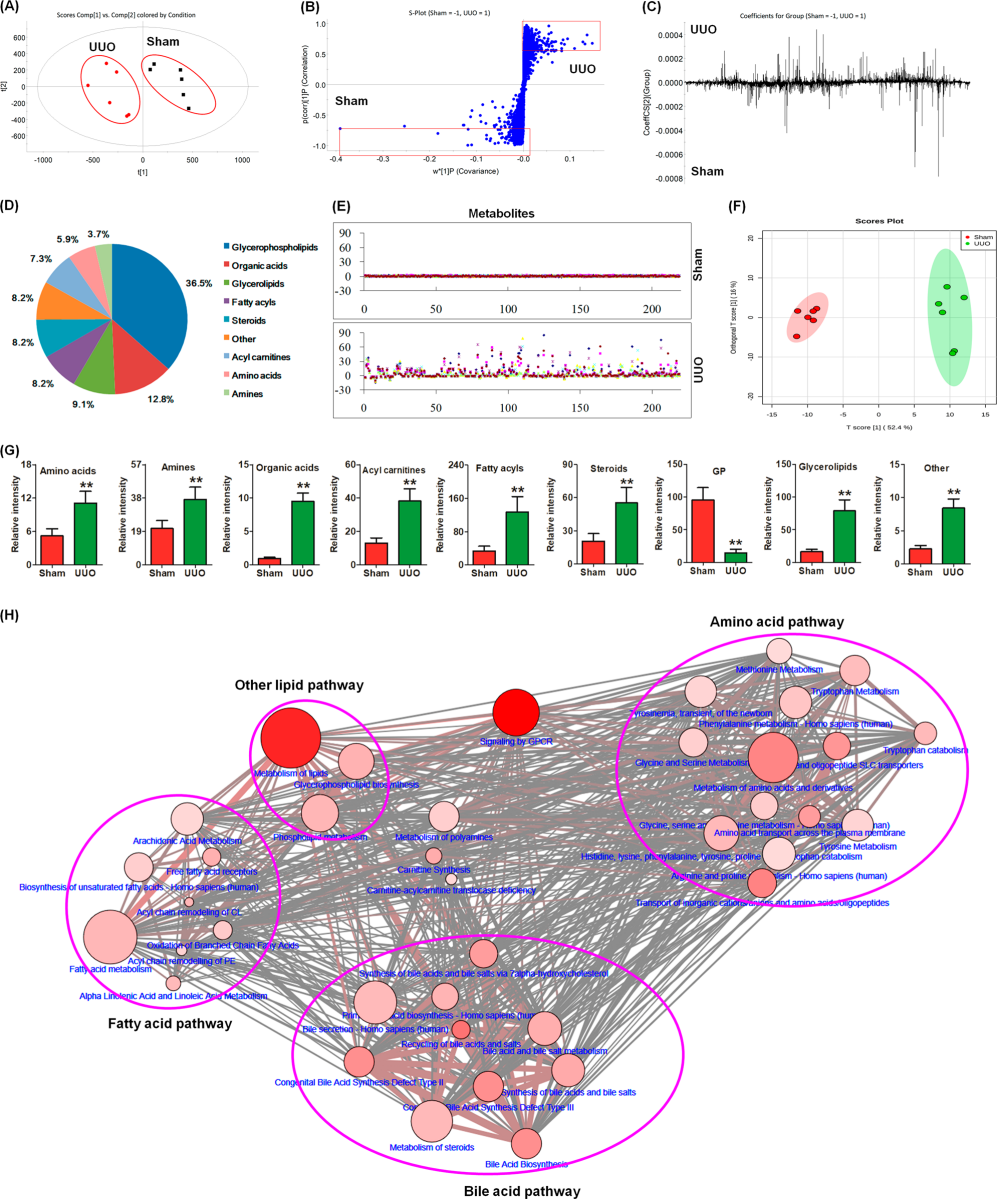
Metabolic profile, multivariate analysis and metabolic pathway of identified metabolites in the UUO rats. **a** PCA plots with the scores of the first two principal components from the UUO and sham rats. **b** S-plots of the OPLS-DA model from the UUO and sham rats. **c** Loading plots of OPLS-DA model from the UUO and sham rats. **d** Pie chart presents the distribution of different classifications of 219 identified metabolites in the UUO and sham rats. **e** Sham-based z-score plot of 219 metabolite alterations in the sham and UUO rats. Each point represents an individual metabolite in one sample. Z-score plots for the data are normalized to the mean of the sham samples. **f** OPLS-DA plots with the scores of the first two principal components based on 219 metabolites from the UUO and sham rats. **g** Relative intensity profiles of total amino acid, amine, organic acid, acyl carnitine, fatty acyl, steroid, glycerophospholipid, glycerolipid, and other metabolic classes in the sham and UUO rats. **h** Metabolite pathway analysis of 219 metabolites by MetScape software running on Cytoscape based on KEGG, Reactome, and SMPDB database

To assess the effects of the changes in the gut microbiota on metabolic pathways, we performed an untargeted plasma metabolomic analysis using novel ultra-performance liquid chromatography coupled with the high-definition mass spectrometry (UPLC-HDMS) technique. Both positive and negative ion modes in HDMS were used to evaluate the plasma samples. The positive ion mode showed higher noise and matrix effects, resulting in higher baseline values. Therefore, the positive ion mode was used for the final analysis and 7408 reproducible peaks were obtained. To evaluate the systemic changes of the metabolome in UUO rats and find important metabolites, a two-predictive component OPLS-DA model (R2X(cum) = 0.9627, Q2(cum) = 0.8160) was generated. Principal component analysis (PCA) showed a clear separation between the UUO and sham groups (Fig. 3a). S-plots and loading plots indicated the influence and contribution of each peak on renal injury in the UUO rats (Fig. 3b, c). A total of 1754 variables were selected based on the S-Plots (Fig. 3b), one-way ANOVA, and the adjusted FDR based on a one-way ANOVA (*P* < 0.05); 219 metabolites were identified based on the criteria established in our previously reported methods (Table S4)^42–44^.

The metabolic classes were mainly grouped into lipids (69.3%), including glycerophospholipids (36.5%), glycerolipids (9.1%) and fatty acyls (8.2%), sterols (8.2%), and acyl carnitines (7.3%) (Fig. 3d). Other metabolites included organic acids (12.8%), amino acids (5.0%), and amines (4.6%) (Fig. 3d). The z-score showed that the plasma metabolites were significantly altered in the UUO rats (Fig. 3e). Orthogonal partial least squares-discriminant analysis (OPLS-DA) plots showed that these metabolites could differentiate the UUO rats from the sham rats (Fig. 3f). Compared to sham rats, UUO rats had different proportions of amino acids, amines, organic acids, acyl carnitines, fatty acyls, steroids, glycerophospholipids, glycerolipids, and metabolites from other classes (Fig. 3g). Figure 3h reveals that alterations of the 219 metabolites were mainly involved in lipid or fatty acid metabolism (branched-chain fatty acid oxidation, α-linoleic acid metabolism, and unsaturated fatty biosynthesis), amino acid metabolism (tryptophan, tyrosine, histidine, lysine, phenylalanine, proline, etc.), and bile acid metabolism (bile secretion, primary bile acid biosynthesis, bile acid, and bile salt metabolism) in UUO rats, which is in line with previous metabolomic findings in CKD^45–47^. Most of the altered metabolic pathways were associated with the gut microbiota metabolism, which indicated the dysbiosis of the gut microbiota in the UUO rats.

### Association of the microbial dysbiosis and dysregulation of metabolites with TIF

**Fig. 4:**
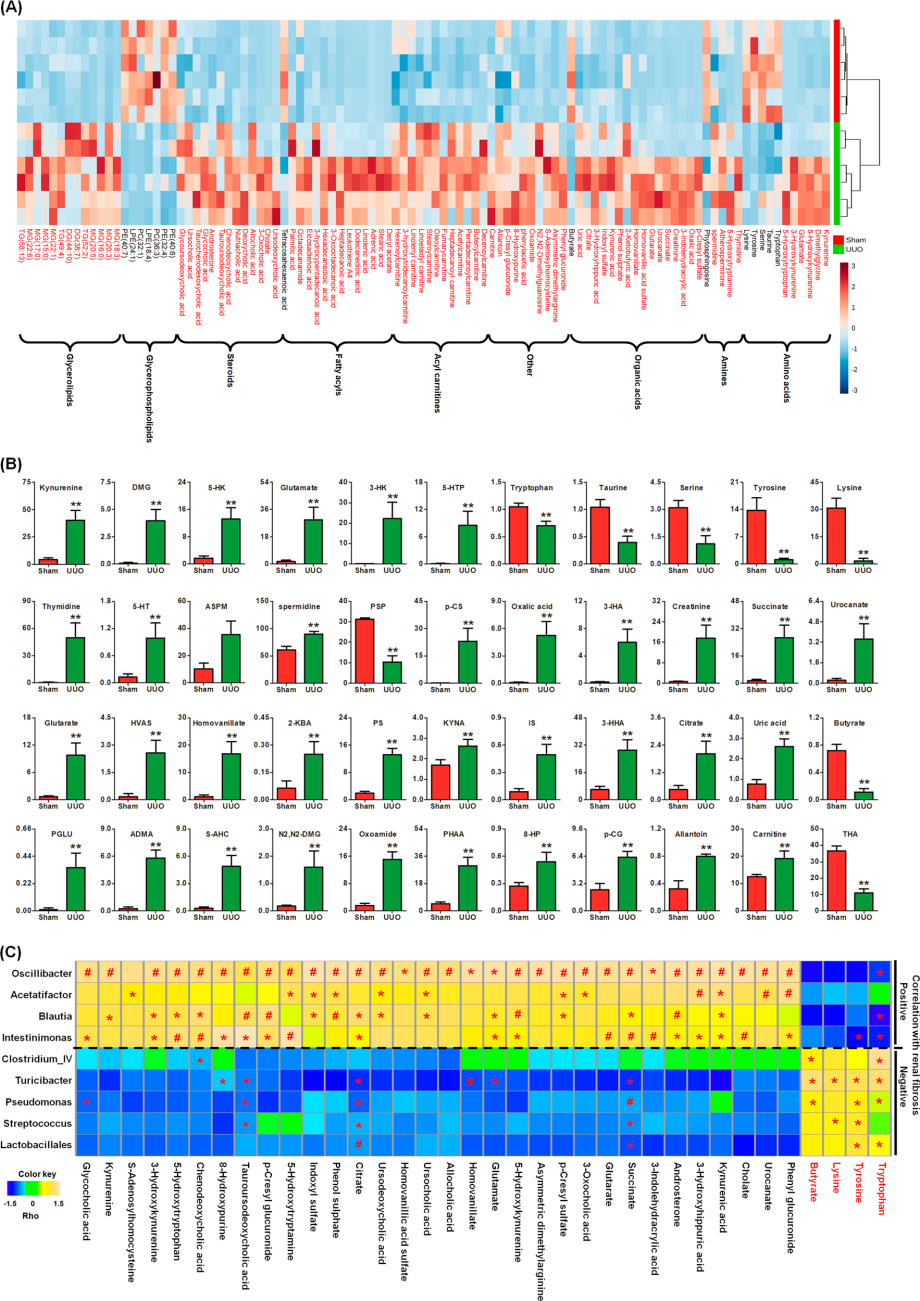
Significantly altered plasma metabolites in UUO rats. **a** Heatmap analysis of 102 metabolites from the UUO and sham rats. Red and black text highlights indicate increased and decreased metabolites in the UUO rats compared to the sham rats. **b** Relative intensities of individual metabolites of amino acids, amines, organic acids, and other class metabolites, as well as THA, in the UUO and sham rats. The vertical axis is the relative intensity of individual metabolites. DMG dimethylglycine, ASPM atherosperminine, PSP phytosphingosine, 3-IHA 3-indolehydracrylic acid, HVAS homovanillic acid sulfate, 2-KBA 2-ketobutyric acid, PS phenol sulfate, PPLA prolylphenylalanine, 3-HHA 3-hydroxyhippuric acid, ADMA asymmetric dimethylarginine, S-ADHC S-adenosylhomocysteine, N2,N2-DMG N2,N2-dimethylguanosine, PHAA phenylacetic acid, 8-HP 8-hydroxypurine, p-CG p-cresyl glucuronide. ^*^P *<* 0.05, ^**^P *<* 0.01 versus sham rats (*n* = 6). **c** Spearman’s rank correlation between the nine most differential genera selected from the LEfSe and 36 differential metabolites (Only the metabolites that were correlated with at least one genus with adjusted *P* < 0.05 are shown). The results are presented as a heatmap using Ward clustering analysis. The scale ranges from + 1.5 (yellow) to −1.5 (blue). Heatmap showing microbial taxa that correlate with metabolites linked positively or negatively to TIF caused by unilateral ureteral obstruction. Rho in the color key represents the Spearman rank correlation coefficient. ^*^P *<* 0.05, ^#^*P* *<* 0.01 denote statistical significance between bacterial taxa and metabolites

To assess the potential role of the altered metabolites in the pathogenesis of TIF, we performed a heatmap analysis of relative intensities of plasma metabolites in UUO and sham rats. One hundred and two metabolites were further selected based on fold changes (UUO/sham > 2.0 or UUO/sham < 0.50) and Mann–Whitney *U* tests (Table S5). Plasma metabolites, including tryptophan, taurine, serine, tyrosine, lysine, phytosphingosine, butyrate, tetracosahexaenoic acid (THA), and glycerophospholipids showed strong negative correlations with TIF (Fig. 4a). The other 87 metabolites were all significantly increased and showed positive correlations with TIF in the UUO rats (Fig. 4a). Most of the increased metabolites belonged to uremic toxins, such as kynurenine, 5-hydroxykynurenine (5-HK), ursocholic acid, 5-hydroxytryptamine (5-HT), p-CS, phenol sulfate, prolylphenylalanine, IS, 3-hydroxyhippuric acid, uric acid, phenyl glucuronide, and p-cresyl glucuronide (Fig. 4b). Significantly increased 5-hydroxytryptophan (5-HTP), dimethylglycine and glutamate, and significantly decreased tryptophan, taurine, serine, tyrosine, and lysine levels, were observed in the UUO rats compared to the sham rats (Fig. 4b). Interestingly, the levels of polyamines, including spermine, spermidine, and atherosperminine, were significantly increased in the UUO rats, which is consistent with the previously reported findings in CKD^48^. In addition, the butyrate and THA levels were significantly decreased in the UUO rats (Fig. 4b).

To further investigate the potential role of the altered gut microbiota and metabolites in the pathogenesis of TIF, we performed a Spearman correlation analysis of altered gut microbiota and plasma metabolite levels with the TIF scores. As shown in Fig. 4c, citrate, succinate, and tauroursodeoxycholic acid (TUDCA) showed a positive correlation with *Oscillibacter*, *Blautia*, and *Intestinimonas* and a negative correlation with *Turicibacter*, *Pseudomonas*, *Streptococcus,* and *Lactobacillales*. Tryptophan, tyrosine, and lysine were significantly decreased in the UUO rats and showed a positive correlation with *Clostridium IV*, *Turicibacter*, *Pseudomonas*, *Streptococcus*, and *Lactobacillales* that harbored genes encoding enzymes involved in amino acid metabolism. The rest of the metabolites, such as kynurenine, kynurenic acid, p-CS, 5-HK, 5-HT, p-cresyl glucuronide, and 5-HTP, showed a positive correlation with *Oscillibacter*, *Acetatifactor*, *Blautia*, and *Intestinimonas*.

**Fig. 5:**
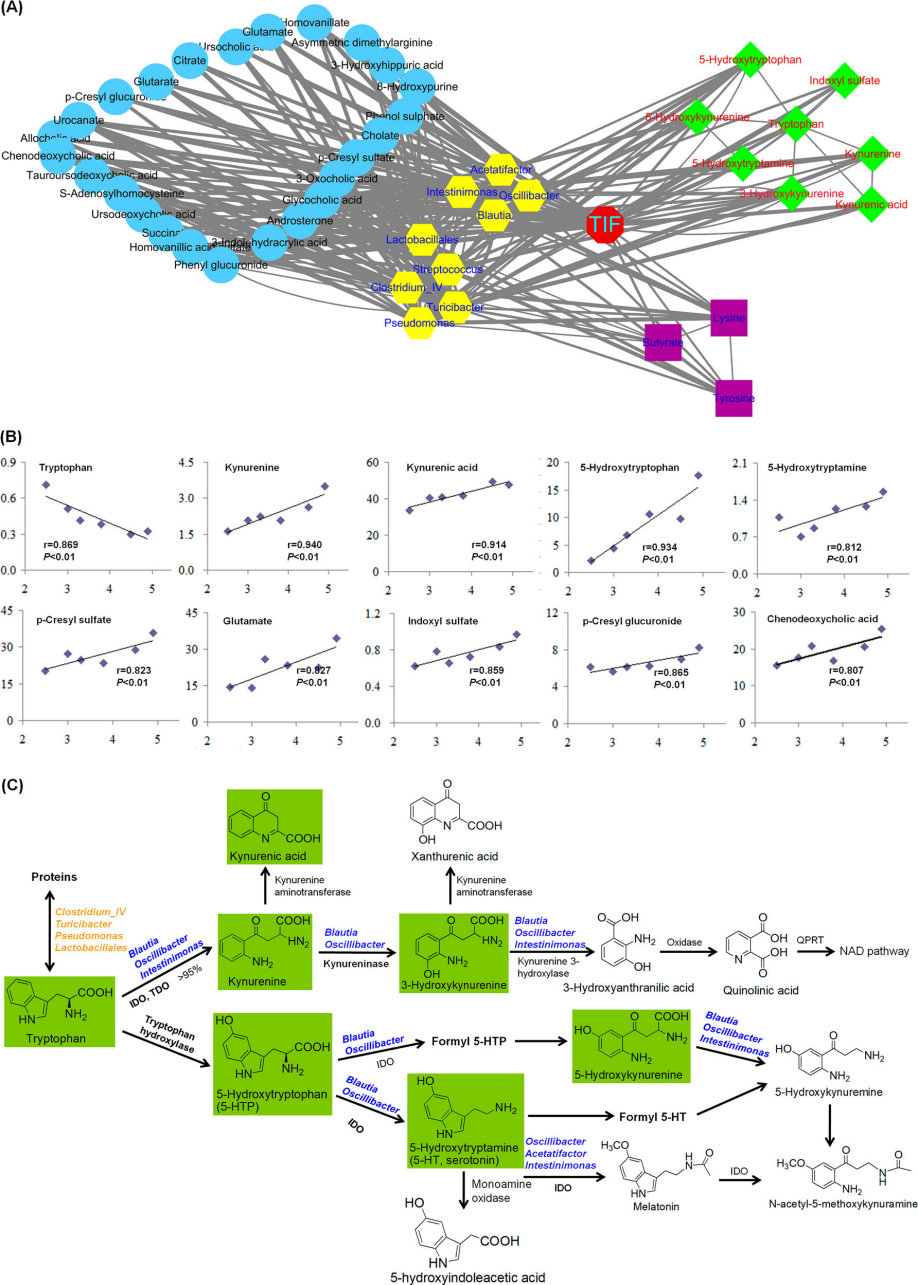
Correlation analysis of the gut microbiome, serum metabolites and tubulointerstitial damage score. **a** Correlation network among 9 genus-level bacterial taxa, TIF, and 36 metabolites by Cytoscape software. The nodes of the network represent the genera (yellow hexagon), TIF (red octagon), and tryptophan metabolites (green diamond), lysine, butyrate and tyrosine (purple rectangle), and other metabolites (blue circle) where the edges correspond to a significant (*P* < 0.05) and positive (Spearman rho < −0.3) or negative (Spearman rho > 0.3) correlation between the nodes. **b** The associations between the tubulointerstitial damage score and nine significantly taxa-associated metabolites in the UUO rats. *P* value was calculated by Spearman rank correlation. **c** A summary of the various pathways of tryptophan metabolism. Highlight tryptophan, kynurenine, 5-HTP, 5-HT, 3-HK, and 5-HK were identified in our current study. NAD nicotinamide, QPRT quinolinic-acid phosphoribosyl transferase

To further explore the association among clinical indices, bacterial genus and metabolites, a network analysis based on 9 genus-level bacterial taxa, TIF, and 36 metabolites were used to highlight the associations of the gut microbiome with pathological indices and plasma metabolites in renal fibrosis by Cytoscape software. As shown in Fig. 5a, TIF directly linked dysbiosis of the gut microbiota and metabolites, which indicated that urinary tract obstruction was associated with altered microbial composition and tryptophan metabolism, events that contributed to renal fibrosis. Gut microbiota was linked to tryptophan metabolism. Interestingly, nine metabolites, including kynurenine, kynurenic acid, 5-HTP, 5-HT, p-cresyl sulfate, glutamate, p-CS, IS, p-cresyl glucuronide, and chenodeoxycholic acid (CDCA), were positively correlated with tubulointerstitial damage scores, whereas tryptophan was negatively correlated with tubulointerstitial damage scores (*r* > 0.80, *P* < 0.01; Fig. 5b). Thus, these metabolites that were linked with TIF were correlated with specific gut microbiota. We further found that kynurenine, kynurenic acid, 5-HTP, 5-HT, 5-HK, and IS are different metabolites of tryptophan. In addition, 3-hydroxykynurenine (3-HK) was also involved in tryptophan metabolism. A summary of the various pathways of tryptophan metabolism is presented in Fig. 5c.

*Clostridium IV*, *Turicibacter*, *Pseudomonas*, and *Lactobacillales*, which may possess the gene encoding K16187, and tryptophan synthase, required to produce tryptophan (Table S6), were positively correlated with plasma tryptophan level. Metabolic pathway analysis showed that enzymes involved in the synthesis and metabolism of tryptophan, including tryptophan synthase, indoleamine 2,3-dioxygenase (IDO), tryptophan 2,3-dioxygenase (TDO), tryptophan 2-monooxygenase, kynurenine 3-monooxygenase, kynureninase, and tryptophanase, were involved in TIF in the UUO rats (Fig. S3; Table S7). Furthermore, plasma tryptophan was inversely correlated with the abundance of *Oscillibacter*, *Blautia*, and *Intestinimonas* (Fig. 5a), which possess the genes encoding IDO (K00463) and TDO (K00453), as well as their corresponding enzymes (EC:1.13.11.52 and EC:1.13.11.11) that convert tryptophan to kynurenine (Fig. 5a; Tables S6, S7). These results suggest that the enrichment of *Oscillibacter*, *Blautia*, and *Intestinimonas* may contribute to the lower levels of plasma tryptophan in the UUO rats. In contrast, TIF-enriched microbial genera may contribute to the higher plasma levels of kynurenine, kynurenic acid, 5-HTP, 5-HT, 3-HK, and 5-HK in the UUO rats (Fig. 5c)

### Effects of ergone (ERG) and PU on gut microbiome, plasma metabolites, and TIF in UUO rats

**Fig. 6:**
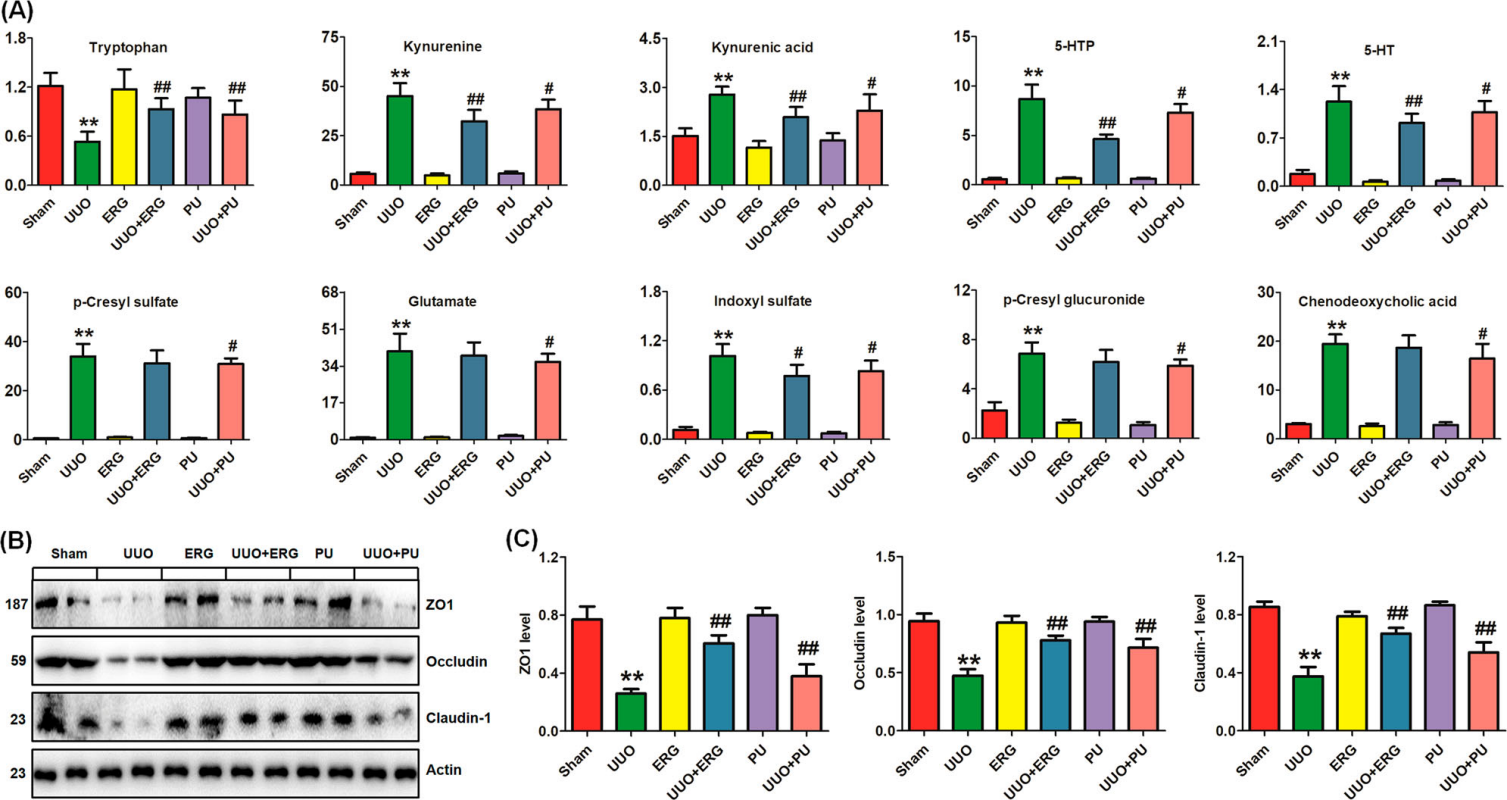
Effects of ergone and PU on plasma metabolites and intestinal barrier in UUO rats. **a** Relative intensity of individual metabolites in the plasma of the different rats. The vertical axis is the relative intensity of individual metabolites. **b** The expression levels of the tight junction protein ZO1, occludin and claudin-1 in the colon of the different rats. Tissue lysates were immunoblotted with specific antibodies against ZO1, occludin, and claudin-1. **c** Graphic representations of ZO1, occludin, and claudin-1 expression in different groups, as indicated. ERG ergone, PU Polyporus umbellatus. ^*^P *<* 0.05; ^**^P *<* 0.01 versus sham rats (*n* = 6); ^#^*P* *<* 0.05, ^##^*P* *<* 0.01 versus UUO rats (*n* = 6)

A potential therapeutic strategy that is being actively studied is the use of probiotics, prebiotics, and antibiotics. PU, known as one of the most widely used and precious medicinal fungi, has been commonly used in medicine for a wide range of ailments related to the edema, scanty urine, vaginal discharge, and urinary dysfunction, as well as jaundice and diarrhea^49^. Our previous studies demonstrated that ergone, as one of the main PU bioactive compounds, possessed diuretic, and renoprotective effects^8,50,51^. Our further metabolomic studies indicated that ergone could improve lipid, amino acid, and purine metabolism in adenine-induced chronic renal failure rats^35,52,53^. Moreover, ergone could improve fecal bile acid metabolism^54^. Ergone treatment restored plasma tryptophan levels and reduced plasma levels of kynurenine, kynurenic acid, 5-HTP, 5-HT, and IS in UUO rats, but it had no effect on p-CS, glutamate, p-cresyl glucuronide, or CDCA levels (Fig. 6a). Although similar results were observed in PU-treated UUO rats, PU treatment restored plasma tryptophan levels and reduced plasma levels of all nine metabolites. Interestingly, ergone showed a stronger inhibitory effect on tryptophan metabolites than PU (Fig. 6a), indicating that ergone treatment had an important effect on the tryptophan metabolism in the intestine. It is possible that the dysregulation of plasma metabolites affected the expression of tight junction proteins. It has been reported that tryptophan enhances expression of tight junction proteins^55^.

### Impact of UUO on the colonic epithelial barrier

CKD results in disruption of colonic epithelial tight junctions. Disruption of the intestinal epithelial barrier has been shown to contribute to increased serum cytokines levels and renal impairment in CKD^10^. Colon epithelial tight junctions maintain the intestinal barrier, wherein the proteins ZO1, occludin, and claudin-1 are the key components of intestinal tight junctions. The expression of all three proteins was lower in the UUO rats compared to the sham rats. Both ergone and PU treatment restored the expression levels of these three proteins in the UUO rats (Fig. 6b, c).

**Fig. 7:**
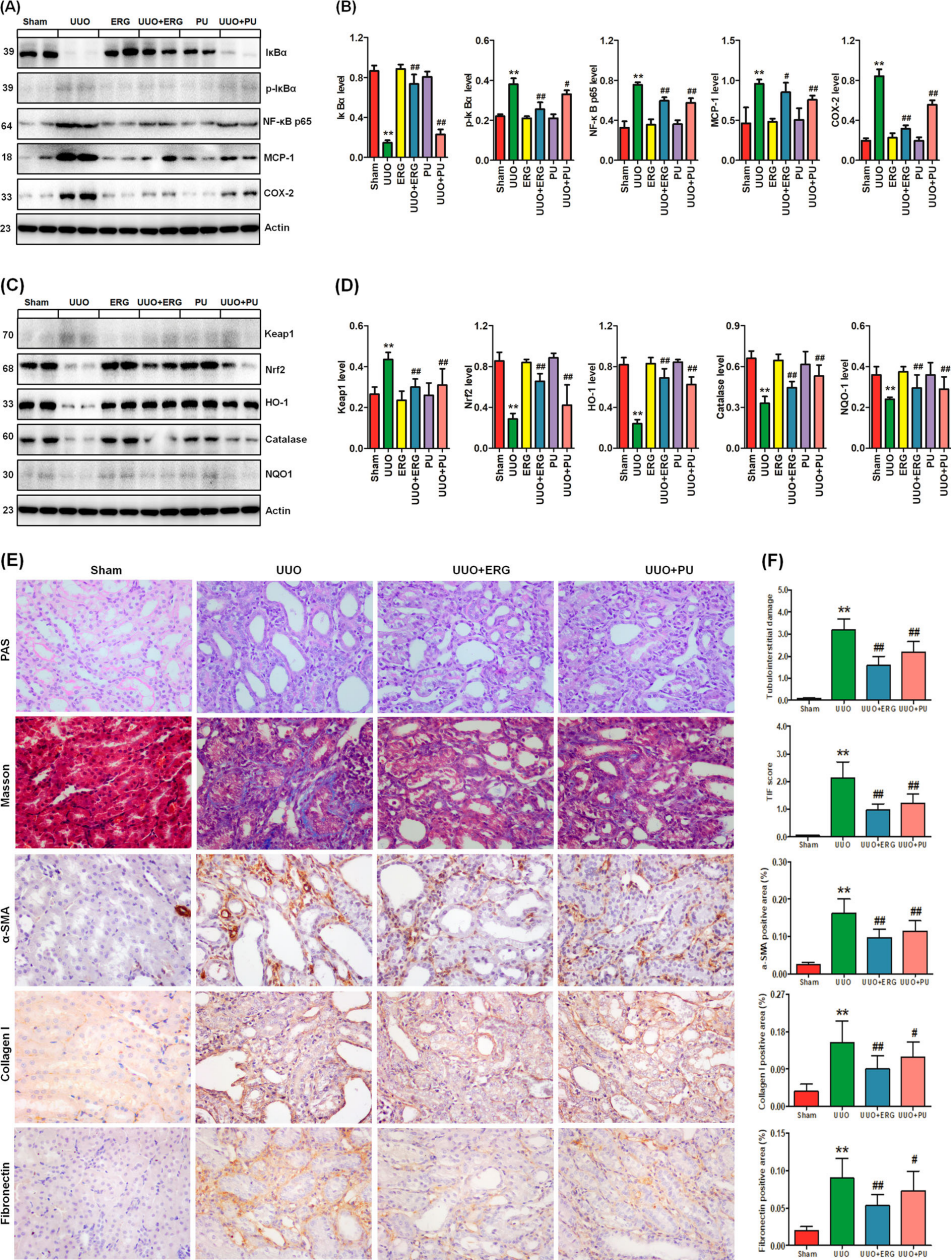
Effects of ergone and PU on inflammation and TIF in UUO rats. **a** Western blot depicting IκBα, p-IκBα, the nuclear content of p65, the active subunit of NF-κB and expression of COX-2 and MCP-1 in kidney tissues of the different rats. Tissue lysates were immunoblotted with specific antibodies against IκBα, p-IκBα, NF-κB, COX-2, and MCP-1. **b** Graphic representations of IκBα, p-IκBα, NF-κB, COX-2, and MCP-1 expression in different groups, as indicated. **c** Western blot depicting nuclear translocation of Nrf2 and protein abundances of its repressor, Keap1, and expression of its downstream gene products, catalase, HO-1 and NQO-1, in kidney tissues of the different rats. Tissue lysates were immunoblotted with specific antibodies against Keap1, Nrf2, catalase, HO-1, and NQO-1. **d** Graphic representations of Keap1, Nrf2, catalase, HO-1, and NQO-1 expression in the different groups, as indicated. **e** Images of PAS staining, Masson’s Trichrome staining and immunohistochemical staining of α-SMA, collagen I, and fibronectin expression in the different rats. **f** Graphic representations of PAS staining, Masson’s Trichrome staining and immunohistochemical staining in the different groups, as indicated. ERG ergone, PU, *Polyporus umbellatus*. ^*^P *<* 0.05; ^**^P *<* 0.01 versus sham rats (*n* = 6); ^#^*P* *<* 0.05, ^##^*P* *<* 0.01 *versus* UUO rats (*n* = 6)

To reveal whether increased intestinal permeability contributed to elevated plasma metabolites and inflammation, the IκB/NF-κB and Keap1/Nrf2 pathways were investigated. As shown in Fig. 7a–d, the UUO rats showed a marked upregulation and nuclear translocation of p65 protein expression, indicating activation of NF-κB signaling. Activation of IκBα/NF-κB was accompanied by significant upregulation of inflammatory genes, including MCP-1 and COX-2, and downregulation of the antioxidant system, including Nrf2 and its downstream gene products (including HO-1, catalase, and NQO1). Both ergone and PU treatment could reverse the dysregulation of the IκB/NF-κB and Keap1/Nrf2 pathways. Interestingly, ergone showed a stronger effect on these protein expression levels than PU (Fig. 7a–d).

Oxidative stress and inflammation play a central role in the pathogenesis and TIF^56^. Compared with the sham rats, the obstructed kidneys of the UUO rats exhibited interstitial inflammatory cell infiltration, tubular dilatation, tubular atrophy, and TIF (Fig. 7e, f). PAS and Masson’s Trichrome staining showed that both ergone and PU treatment significantly ameliorated renal injury and TIF in UUO rats (Fig. 7e, f). Immunohistochemistry staining showed that both ergone and PU treatments inhibited the upregulation of profibrotic factors α-SMA, collagen I, and fibronectin (Fig. 7e, f). Interestingly, ergone showed a stronger antifibrotic effect than PU. Taken together, these results suggested that the restoration of a disrupted intestinal barrier by improving plasma metabolite dysregulation could retard TIF by inhibiting tryptophan-derived metabolite-induced inflammation and oxidative stress by ergone and PU treatment.

## Discussion

TIF is a major histological feature of CKDs of diverse etiologies. Urinary tract obstruction results in TIF by inducing hydronephrosis, ischemia, and inflammation. CKD adversely affects the intestinal environment^15,57^ by a variety of factors such as malnutrition, intestinal ischemia, metabolite dysregulation (especially retained uremic toxins/metabolites), decreased intestinal fluid secretion, and prolonged intestinal transit time, events that lead to the dysbiosis of the gut microbiota^58^. The gut microbial dysbiosis and the resulting dysregulation of metabolites and bacterial translocation contribute to the pathogenesis of TIF^58^.

In this study, we sought to explore the effect of UUO on the gut microbiota and the dysregulation of metabolites and their role in the pathogenesis of TIF. The changes in the gut microbiota and dysregulation of plasma metabolites in this study pointed to an association of the gut microbial dysbiosis with altered metabolisms of glycerophospholipids, glycerolipids, fatty acyls, sterols, acyl carnitines, organic acids, amino acids, and amines. Several metabolites, including kynurenic acid, allantoin, and kynurenine have been identified and reported in UUO rats, which was consistent with our current study^59^. The plasma levels of these metabolites reflect the balance between the rates of their generation and elimination. Some studies indicated that most of the microbial-derived metabolites are protein-bound compounds^60^. Hence, their elimination would depend on the tubular transporter system. Increasing evidence indicates that the gut microbiota largely contributes to the production of various gut-derived uremic toxins, such as IS from indole derivatives generated from the fermentation of tryptophan, p-cresyl glucuronide, and p-cresyl sulfate from p-cresol conjugates generated from the fermentation of tyrosine^10^. Uremic toxins are implicated in TIF through exacerbation of oxidative stress and inflammation^61,62^. Therefore, gut microbial dysbiosis might be associated with TIF.

The gut microbiome of the UUO rats was clearly different from that of the sham rats. Rats with TIF had more opportunistic pathogens, such as *Oscillibacter*, *Intestinimonas*, *Blautia*, and *Acetatifactor*, and fewer commensal or beneficial genera, including *Turicibacter*, *Clostridium IV*, *Streptococcus*, *Pseudomonas*, and *Lactobacillales*. At the genus level, we detected a depletion of *Turicibacter*, *Clostridium IV*, *Streptococcus*, and *Pseudomonas* in the UUO rats. The *Turicibacter* genus only includes one species, *Turicibacter sanguinis*, which is a strictly anaerobic and gram-positive bacteria first isolated from the blood of febrile patients with acute appendicitis^63,64^. Subsequently, *T. sanguinis* was identified in a healthy human fecal sample^65^. *T. sanguinis* ferments maltose and 5-ketogluconate as the only carbohydrates and produces only lactate and minimal amounts of acetate^63^. *Turicibacter* spp. has anti-inflammatory properties^66^ and its decreased abundance has been reported in BALB/C mice with adriamycin-induced nephropathy^40^ and in 5/6 nephrectomized rats^39^, which is consistent with our current study. Dysregulation of *Turicibacter* was found in adenine-induced CKD rats and was reversed with high-fiber diets^67^. *Streptococcus* is composed of three species and one species was assigned to *Streptococcus salivarius subsp. salivarius*. The depletion of *Streptococcus* has been observed in 5/6 nephrectomized rats compared to sham rats^39^. In CKD patients, the depletion of *Streptococcus* in feces was positively associated with eGFR decline^41^. In addition, the lower abundance of *Streptococcus* in the saliva also correlated with lower eGFR^68^.

The *Eubacterium siraeum* species from the *Clostridium IV* genus of Ruminococcaceae was significantly decreased in our UUO rats. *E. siraeum* has been isolated from human feces and its relative abundance is 0.011% of all sequences and 0.217% of the sequences in its division^69,70^. It has been reported that *Clostridium IV* is significantly decreased in the stool samples from patients with chronic hepatitis B compared to healthy controls^71^. A depletion of *Clostridium* but not *Clostridium IV* has been found in the feces of ESRD patients^72^. The *Pseudomonas* genus was significantly decreased in our UUO rats, which was consistent with previous studies in ESRD patients^72,73^. However, another study showed increased *Pseudomonas* in blood samples of ESRD patients^74^. We suppose that such discrepancies result from the differences between mammalian species. The bacterial genera that contribute to the renal fibrosis in rats may differ from those of human ESRD. One species, *Pseudomonas psychrophile*, which is assigned to the *Pseudomonas* genus, was significantly decreased in our UUO rats. In addition, the depletion of the *Lactobacillales* order was observed in our UUO rats, which was reported to be positively correlated with eGFR decline in CKD patients^41^. The five taxa showed positive correlations with the levels of plasma tryptophan, tyrosine and lysine and negative correlations with the levels of plasma citrate, succinate and TUDCA in our UUO rats. Interestingly, tryptophan and its metabolites kynurenine, 5-HTP, 5-HT, and 5-HK, which were linked with TIF, were correlated with nine specific gut microbiota components. These taxa possess the genes encoding IDO and TDO and their corresponding enzymes and could release tryptophan from proteins (Fig. 5c). The depletion of commensal taxa, including *Turicibacter*, *Clostridium IV*, *Streptococcus*, *Pseudomonas*, and *Lactobacillales*, could have contributed to the decreased plasma levels of beneficial anti-TIF metabolites, such as butyrate, tryptophan, THA, and taurine, in the UUO rats^75,76^.

We detected an enrichment of the *Blautia*, *Oscillibacter*, *Intestinimonas*, and *Acetatifactor* genera in our UUO rats. These taxa genera were also positively correlated with the severity of TIF. Of the four genera that were increased in UUO rats, *Oscillibacter* was in higher abundance in the UUO rats. In our study, *Oscillibacter* was composed of eight species, of which, four species were assigned to *Oscillibacter valericigenes*. *Oscillibacter* spp. is a strictly anaerobic bacteria and *O*. *valericigenes* could produce valerate as its major product^77^. The increased abundance of the *Oscillibacter* genus has been reported in adriamycin-induced BALB/C mice^40^ and patients with stroke and transient ischemic attack^78^. Although the *Blautia* genus was increased significantly in our UUO rats, its relative abundance was low in both groups (0.019% and 0.074% in the sham and UUO rats, respectively). This genus is composed of 5 OTUs, of which, two were assigned to the *B. faecis* and *B. wexlerae* species and the rest were assigned to *Blautia* spp. Zeng et al. reported that *Blautia* was significantly augmented in 5/6 nephrectomized rats^39^. In ESRD patients, *Blautia* was significantly decreased at day 7 and returned to baseline or above at day 28 after vancomycin administration, accompanied by the decline and subsequent rebound of plasma IS and p-CS levels^79^. This result indicated that specific tryptophan or tyrosine suppression could lower the microbiota by an increase in competing taxa in the vancomycin-treatment gut. However, the levels of recovered uremic toxins at day 28 indicated the resilience of the taxa responsible for its generation in patients with ESRD^79^. In addition, *Blautia* was associated with an early decline of renal function (eGFR decline)^80^. Although most of the direct functional evidence points to a role of the pathogenic *Blautia* and *Oscillibacter* in dysregulation of the host’s inflammatory response, the commensal *Blautia* and *Oscillibacter* may represent the major proportion of the gut microbiome. We also detected an enrichment of *Intestinimonas* and *Acetatifactor* (both belonging to Firmicutes phylum) in the UUO rats. The two genera were both composed of only one OTU assigned to *Intestinimonas butyriciproducens* and *Acetatifactor muris*, respectively. So far, a few studies have reported the presence of *Intestinimonas* and *Acetatifactor*, such as *I. butyriciproducens* in the mouse intestine^81^ and *A. muris* in the intestine of an obese mouse^82^. The present study was the first to detect *I. butyriciproducens* and *A. muris* in the colonic lumen of a rat model of renal injury. Except for tryptophan, these taxa positively correlated with most of the plasma metabolites, such as kynurenine, kynurenic acid, 5-HTP, 5-HT, 3-HK, 5-HK, IS, p-CS, CDCA, and TUDCA, that exacerbated TIF in the UUO rats, confirming the results of our previous findings in patients with CKD^61,62,83^. Our study further demonstrated that both ergone and PU treatment could improve these metabolites by mitigating disruption of the intestinal barrier via upregulating expression of intestinal tight junction proteins including ZO1, occludin, and claudin-1. Both ergone and PU could alleviate α-SMA, collagen I, and fibronectin expression by inhibiting abnormal expression of plasma metabolite-induced pro-inflammatory and pro-oxidant proteins p-IκBα, NF-κB p65, MCP-1, and COX-2, n as well as retaining the antioxidant system, including Nrf2, HO-1, catalase, and NQO1 expression.

We presumed that metabolites produced by the gut microbiota contribute to the gut barrier dysfunction and inflammation. The metabolic profiles identified by PCA were clearly distinct between the UUO and the sham rats. Overall, this observation supported the relationship between metabolites and intestinal permeability. Basically, two types of microbial fermentation occur in the colon: saccharolytic fermentation and proteolytic fermentation^84^. The former is commonly considered to be beneficial to the host, and the latter is supposed to be harmful and might be involved in the etiology of various diseases. Our findings demonstrated that the depletion of beneficial genera, including *Turicibacter*, *Clostridium IV*, *Streptococcus*, *Pseudomonas*, and *Lactobacillales*, resulted in decreased plasma tryptophan levels in the UUO rats. SCFA butyrate, the main byproduct of carbohydrate fermentation, was significantly decreased in the UUO rats and was related to intestinal permeability. Metabolite differences between the UUO and sham rats were mainly the result of protein fermentation that led to the formation of indole-derivative toxic metabolites (i.e., phenolic and sulfur-containing compounds) and branched-chain fatty acids. The production of phenolic metabolites in the gut depends on microbial composition or metabolic activities^85^. Tyrosine-derived phenol conjugates were largely increased in the UUO rats. The demonstrated toxicities of phenol conjugates on intestinal epithelial cells suggest that phenol conjugates are a potential driver of gut barrier alterations. This effect could be related to the observed expansion of *Blautia*, *Oscillibacter*, *Intestinimonas*, and *Acetatifactor* in the UUO rats because these taxa have been shown to produce indole, p-cresol, and tryptophan derivatives^79,80^. Bacterial metabolism of tryptophan produces a large variety of indole derivatives^86^. The higher enrichment of these taxa resulted in increased plasma levels of kynurenine, kynurenic acid, 5-HTP, 5-HT, and 5-HK, which exacerbated TIF in the UUO rats. Taken together, these observations indicated the detrimental role of bacteria producing toxic metabolites in TIF aggravation.

In summary, UUO results in distinctive changes in colonic microbiota composition and plasma metabolites that can contribute to TIF. Ten plasma metabolites linked with TIF were correlated with nine specific genera. Interestingly, plasma tryptophan levels were positively correlated with *Clostridium IV*, *Turicibacter*, *Pseudomonas*, and *Lactobacillales*, and negatively correlated with *Oscillibacter*, *Blautia*, and *Intestinimonas*, which possess genes encoding tryptophan synthase, IDO, and TDO and their corresponding enzymes that exacerbate TIF. The restoration of the disrupted intestinal barrier by improving plasma metabolite dysregulation could retard TIF by inhibiting tryptophan-derived metabolite-induced inflammation and oxidative stress by ergone and PU treatment. A better understanding of the role of the gut microbiome and plasma metabolites in kidney function may shed new light on the pathogenesis of renal diseases and help in the designing of novel interventions aimed at restoring symbiosis to treat or prevent renal fibrosis.

## Supplementary information

Supplementary Figures

Supplementary Tables
