# Supplementary material for: Unilateral ureteral obstruction causes gut microbial dysbiosis and metabolome disorders contributing to tubulointerstitial fibrosis
Source: Exp Mol Med. 2019 Mar 27;51(3):38. doi: 10.1038/s12276-019-0234-2 (PMC6437207; doi:10.1038/s12276-019-0234-2)
Supplement: Supplementary file 1 — Supplementary Figures [file 12276_2019_234_MOESM1_ESM.docx]

**Supplementary Material**


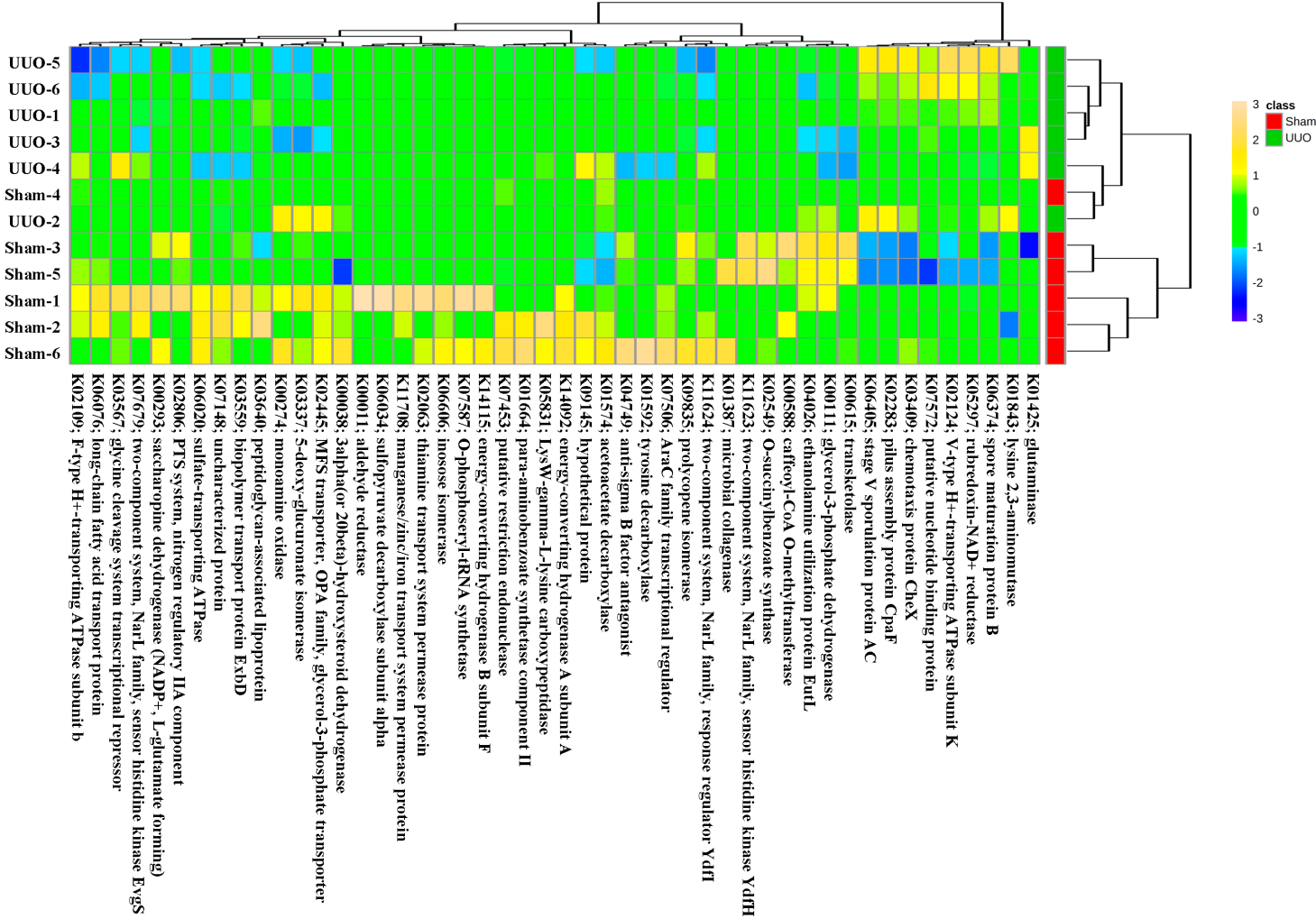


**Figure S1.** Heatmap of KEGG Orthology analysis obtained from PICRUSt analysis of 16S rRNA sequencing data from UUO rats *versus* sham rats.


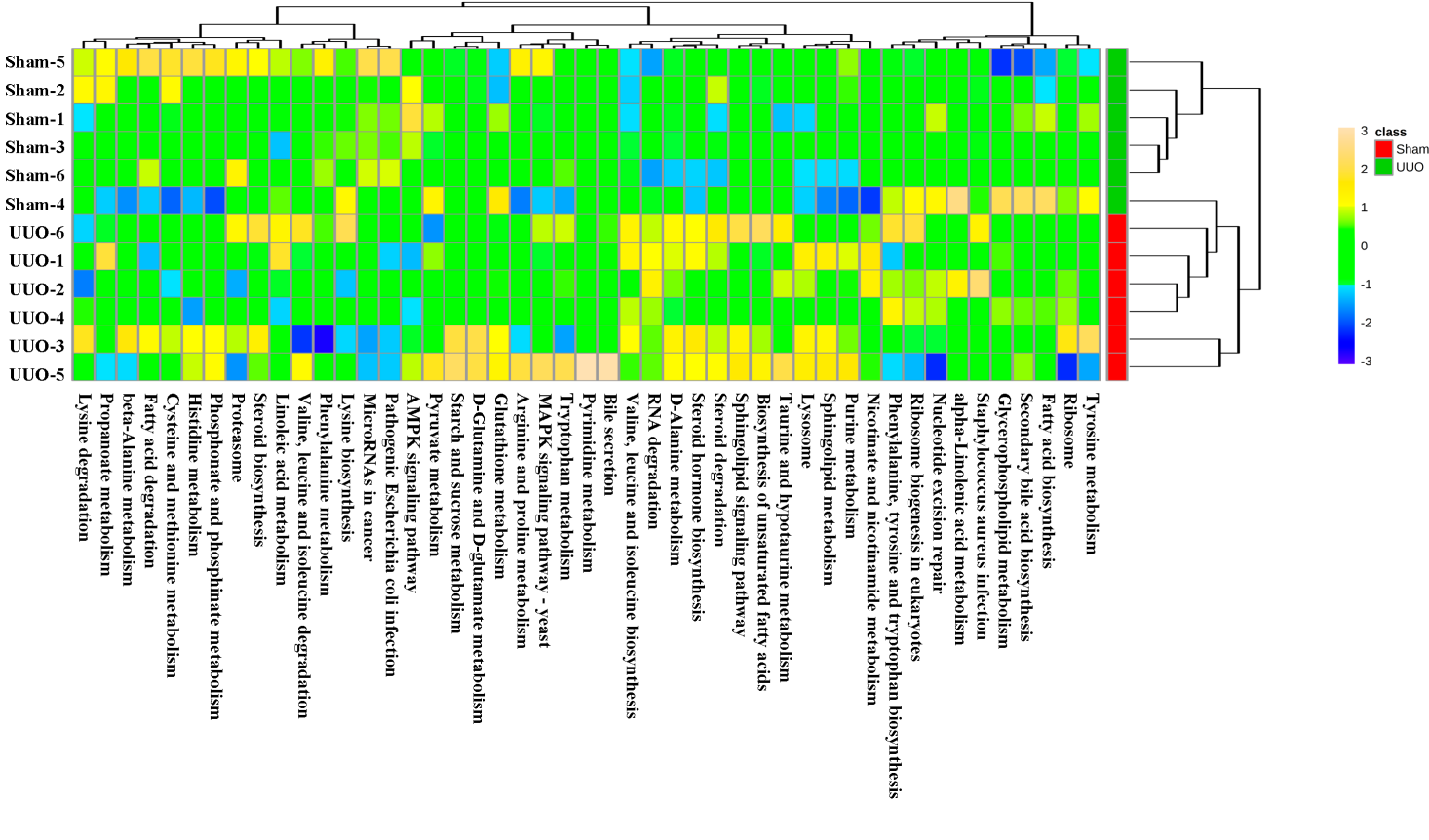


**Figure S2.** Heatmap of significantly altered metabolic pathways obtained from PICRUSt analysis of 16S rRNA sequencing data from UUO rats *versus* sham rats.


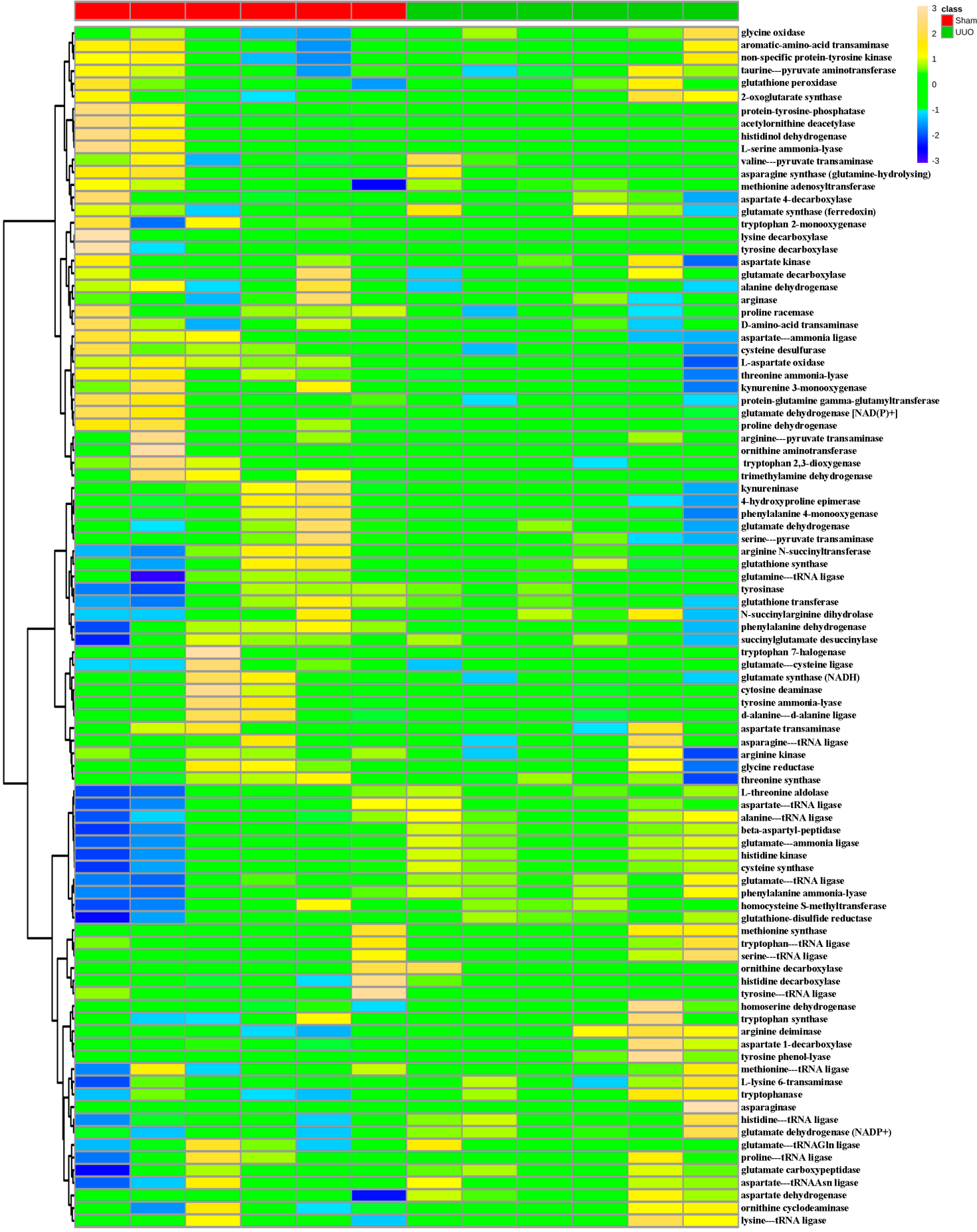


**Figure S3.** Heatmap of significantly altered amino acid biosynthesis and metabolism-associated enzymes obtained from PICRUSt analysis of 16S rRNA sequencing data from UUO rats *versus* sham rats.


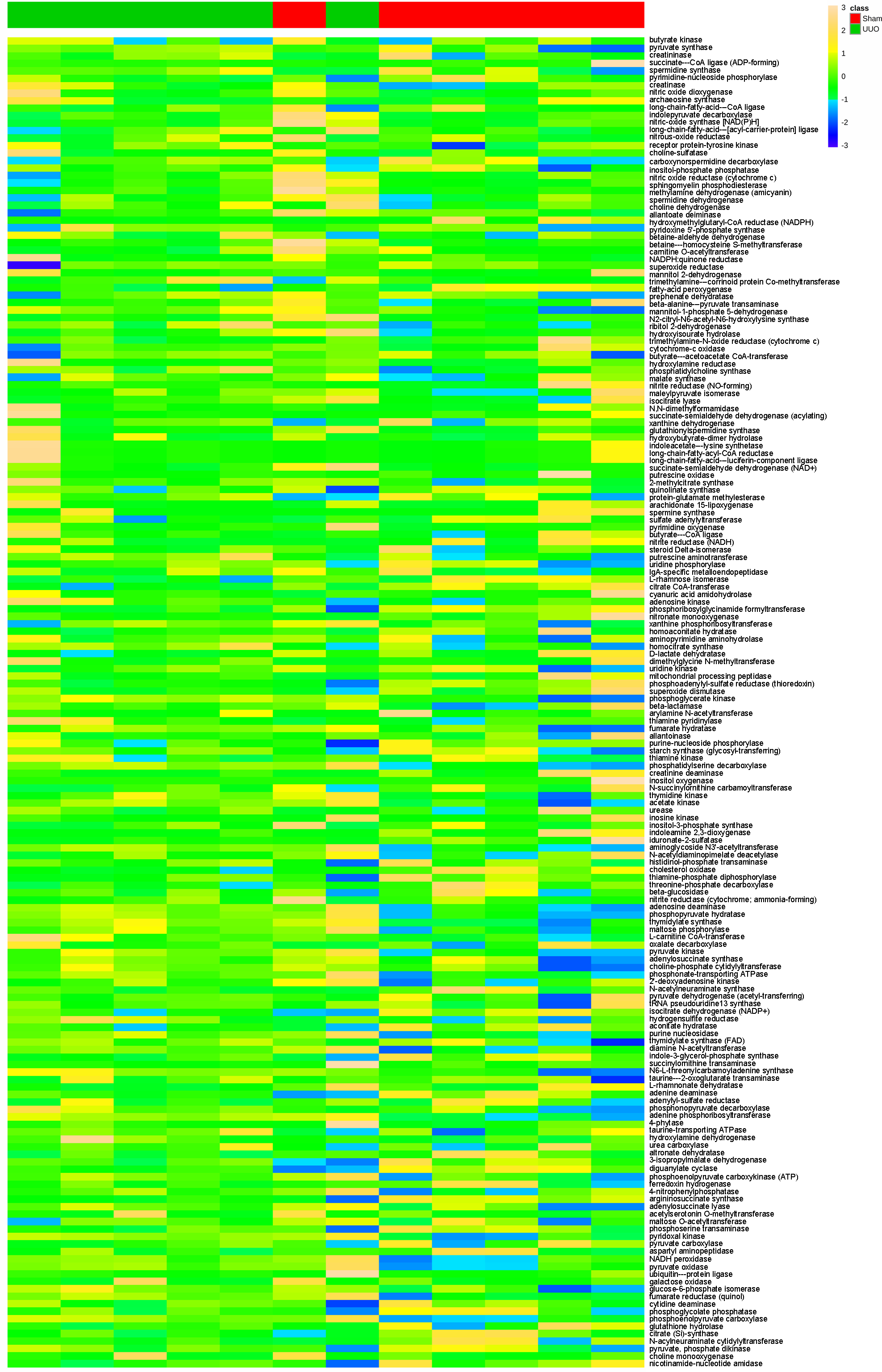


**Figure S4.** Heatmap of altered enzymes (not amino acid biosynthesis and metabolism-associated enzymes) in metabolic pathways obtained from PICRUSt analysis of 16S rRNA sequencing data from UUO rats *versus* sham rats.
