# Supplementary material for: Unilateral ureteral obstruction causes gut microbial dysbiosis and metabolome disorders contributing to tubulointerstitial fibrosis
Source: Exp Mol Med. 2019 Mar 27;51(3):38. doi: 10.1038/s12276-019-0234-2 (PMC6437207; doi:10.1038/s12276-019-0234-2)
Supplement: Supplementary file 2 — Supplementary Tables [file 12276_2019_234_MOESM2_ESM.docx]

**Supplementary Material**

Table S1. The indices including Richness, Chao1, Shannon_2, Simpson, Dominance and Equitability of gut microbiota in sham and UUO groups

|  | Richness | Chao1 | Shannon_2 | Simpson | Dominance | Equitability |
| --- | --- | --- | --- | --- | --- | --- |
| Sham | 568.8±20.6 | 570.6±20.3 | 6.53±0.44 | 0.034±0.019 | 0.967±0.019 | 0.713±0.046 |
| UUO | 558.7±15.5 | 559.9±15.5 | 6.48±0.39 | 0.036±0.018 | 0.964±0.018 | 0.710±0.042 |
| *P* | 0.178 | 0.164 | 0.417 | 0.412 | 0.411 | 0.444 |

Table S2. Colonic lumenal content 16S rDNA Sequencing from UUO vs. sham rats, Related to Figure 1

| #OTU ID | Sham-1 | Sham-2 | Sham-3 | Sham-4 | Sham-5 | Sham-6 | UUO-1 | UUO-2 | UUO-3 | UUO-4 | UUO-5 | UUO-6 | Taxonomy |
| --- | --- | --- | --- | --- | --- | --- | --- | --- | --- | --- | --- | --- | --- |
| OTU_1 | 1903 | 3635 | 409 | 3681 | 41 | 4130 | 5150 | 3468 | 5761 | 10790 | 1568 | 7579 | k:Bacteria,p:Firmicutes,c:Erysipelotrichia,o:Erysipelotrichales,f:Erysipelotrichaceae |
| OTU_2 | 5 | 13 | 8114 | 55 | 4949 | 792 | 66 | 58 | 30 | 54 | 1 | 57 | k:Bacteria,p:Firmicutes,c:Bacilli,o:Bacillales,f:Bacillaceae_1,g:Bacillus,s:Bacillus_anthracis |
| OTU_3 | 4 | 3 | 8509 | 54 | 4973 | 916 | 69 | 39 | 17 | 43 | 0 | 45 | k:Bacteria,p:Firmicutes,c:Bacilli,o:Lactobacillales,f:Streptococcaceae,g:Lactococcus,s:Lactococcus_piscium |
| OTU_4 | 961 | 590 | 309 | 1348 | 170 | 410 | 1561 | 2652 | 1345 | 151 | 212 | 1052 | k:Bacteria,p:Bacteroidetes,c:Bacteroidia,o:Bacteroidales |
| OTU_5 | 427 | 1733 | 37 | 594 | 3521 | 964 | 59 | 164 | 457 | 1824 | 210 | 1112 | k:Bacteria,p:Firmicutes,c:Bacilli,o:Lactobacillales,f:Lactobacillaceae,g:Lactobacillus,s:Lactobacillus_taiwanensis |
| OTU_6 | 130 | 194 | 2623 | 2092 | 973 | 355 | 1432 | 1413 | 1460 | 448 | 1380 | 2030 | k:Bacteria,p:Firmicutes,c:Clostridia,o:Clostridiales,f:Eubacteriaceae,g:Eubacterium,s:Eubacterium_coprostanoligenes |
| OTU_7 | 244 | 451 | 169 | 1609 | 47 | 822 | 583 | 518 | 885 | 1351 | 258 | 820 | k:Bacteria,p:Firmicutes,c:Erysipelotrichia,o:Erysipelotrichales,f:Erysipelotrichaceae |
| OTU_8 | 230 | 134 | 557 | 44 | 39 | 787 | 447 | 1776 | 696 | 1020 | 986 | 765 | k:Bacteria,p:Firmicutes |
| OTU_9 | 131 | 11 | 216 | 87 | 1 | 43 | 43 | 38 | 25 | 5267 | 12 | 112 | k:Bacteria,p:Firmicutes,c:Clostridia,o:Clostridiales,f:Lachnospiraceae |
| OTU_10 | 3 | 35 | 14 | 105 | 50 | 1 | 986 | 501 | 621 | 340 | 2447 | 191 | k:Bacteria,p:Bacteroidetes,c:Bacteroidia,o:Bacteroidales,f:Porphyromonadaceae |
| OTU_11 | 423 | 697 | 98 | 2046 | 931 | 1497 | 1352 | 1112 | 1125 | 786 | 1901 | 2478 | k:Bacteria,p:Firmicutes,c:Clostridia,o:Clostridiales,f:Ruminococcaceae |
| OTU_12 | 405 | 160 | 84 | 322 | 23 | 119 | 133 | 65 | 243 | 1991 | 91 | 233 | k:Bacteria,p:Firmicutes,c:Erysipelotrichia,o:Erysipelotrichales,f:Erysipelotrichaceae,g:Allobaculum,s:Allobaculum_stercoricanis |
| OTU_13 | 129 | 121 | 861 | 275 | 219 | 354 | 108 | 1168 | 168 | 23 | 83 | 283 | k:Bacteria,p:Firmicutes,c:Clostridia,o:Clostridiales,f:Lachnospiraceae,g:Ruminococcus2 |
| OTU_14 | 781 | 115 | 55 | 916 | 267 | 221 | 947 | 547 | 19 | 16 | 113 | 25 | k:Bacteria,p:Firmicutes,c:Clostridia,o:Clostridiales,f:Lachnospiraceae,g:Clostridium_XlVa |
| OTU_15 | 56 | 110 | 550 | 1050 | 533 | 1166 | 458 | 533 | 295 | 411 | 226 | 351 | k:Bacteria,p:Firmicutes,c:Clostridia,o:Clostridiales,f:Peptostreptococcaceae,g:Romboutsia |
| OTU_16 | 396 | 238 | 410 | 226 | 756 | 133 | 298 | 418 | 303 | 443 | 285 | 350 | k:Bacteria,p:Bacteroidetes,c:Bacteroidia,o:Bacteroidales,f:Porphyromonadaceae |
| OTU_17 | 297 | 114 | 209 | 430 | 854 | 188 | 413 | 1347 | 745 | 110 | 875 | 131 | k:Bacteria,p:Bacteroidetes,c:Bacteroidia,o:Bacteroidales,f:Porphyromonadaceae |
| OTU_18 | 340 | 191 | 383 | 260 | 124 | 1852 | 777 | 474 | 736 | 839 | 966 | 826 | k:Bacteria,p:Firmicutes,c:Clostridia,o:Clostridiales,f:Ruminococcaceae |
| OTU_19 | 66 | 36 | 15 | 9 | 2716 | 18 | 17 | 2 | 5 | 7 | 3 | 26 | k:Bacteria,p:Bacteroidetes,c:Bacteroidia,o:Bacteroidales,f:Porphyromonadaceae |
| OTU_20 | 53 | 86 | 11 | 2037 | 8 | 265 | 24 | 11 | 65 | 158 | 32 | 53 | k:Bacteria,p:Firmicutes,c:Erysipelotrichia,o:Erysipelotrichales,f:Erysipelotrichaceae,g:Allobaculum,s:Allobaculum_stercoricanis |
| OTU_21 | 393 | 146 | 46 | 119 | 9 | 21 | 1017 | 443 | 479 | 10 | 421 | 284 | k:Bacteria,p:Bacteroidetes,c:Bacteroidia,o:Bacteroidales,f:Prevotellaceae,g:Prevotella |
| OTU_22 | 139 | 368 | 57 | 21 | 1 | 25 | 205 | 293 | 200 | 187 | 782 | 264 | k:Bacteria,p:Firmicutes,c:Negativicutes,o:Selenomonadales,f:Acidaminococcaceae,g:Phascolarctobacterium,s:Phascolarctobacterium_succinatutens |
| OTU_23 | 209 | 1137 | 173 | 42 | 93 | 532 | 52 | 0 | 765 | 275 | 107 | 212 | k:Bacteria |
| OTU_24 | 80 | 72 | 120 | 613 | 250 | 505 | 681 | 866 | 464 | 517 | 278 | 619 | k:Bacteria,p:Firmicutes,c:Clostridia,o:Clostridiales,f:Ruminococcaceae |
| OTU_25 | 77 | 411 | 36 | 12 | 130 | 6 | 215 | 1090 | 74 | 35 | 120 | 16 | k:Bacteria,p:Bacteroidetes,c:Bacteroidia,o:Bacteroidales,f:Prevotellaceae |
| OTU_26 | 4 | 8 | 2 | 3 | 2 | 1970 | 15 | 27 | 31 | 109 | 16 | 262 | k:Bacteria,p:Firmicutes,c:Clostridia,o:Clostridiales,f:Ruminococcaceae |
| OTU_27 | 2 | 67 | 165 | 1299 | 306 | 526 | 16 | 24 | 14 | 23 | 1 | 0 | k:Bacteria,p:Firmicutes,c:Clostridia,o:Clostridiales,f:Lachnospiraceae |
| OTU_28 | 334 | 1390 | 88 | 0 | 6 | 5 | 33 | 16 | 21 | 5 | 3 | 58 | k:Bacteria,p:Bacteroidetes,c:Bacteroidia,o:Bacteroidales,f:Prevotellaceae,g:Prevotella,s:Prevotella_copri |
| OTU_29 | 4 | 26 | 274 | 16 | 252 | 3 | 878 | 23 | 48 | 3 | 870 | 1 | k:Bacteria,p:Firmicutes,c:Clostridia,o:Clostridiales,f:Lachnospiraceae |
| OTU_30 | 23 | 55 | 25 | 278 | 77 | 576 | 131 | 155 | 340 | 186 | 170 | 458 | k:Bacteria,p:Firmicutes,c:Clostridia,o:Clostridiales,f:Lachnospiraceae |
| OTU_31 | 239 | 219 | 1005 | 939 | 253 | 34 | 418 | 1069 | 93 | 756 | 117 | 152 | k:Bacteria,p:Firmicutes,c:Clostridia,o:Clostridiales,f:Lachnospiraceae |
| OTU_32 | 4 | 79 | 275 | 0 | 1 | 28 | 202 | 196 | 235 | 397 | 124 | 693 | k:Bacteria,p:Firmicutes,c:Clostridia,o:Clostridiales,f:Ruminococcaceae,g:Clostridium_IV |
| OTU_33 | 133 | 229 | 23 | 407 | 48 | 508 | 111 | 32 | 135 | 87 | 36 | 243 | k:Bacteria,p:Firmicutes,c:Erysipelotrichia,o:Erysipelotrichales,f:Erysipelotrichaceae |
| OTU_34 | 32 | 3 | 20 | 66 | 87 | 342 | 225 | 156 | 173 | 361 | 214 | 148 | k:Bacteria,p:Firmicutes,c:Clostridia,o:Clostridiales |
| OTU_35 | 793 | 155 | 84 | 74 | 221 | 54 | 33 | 61 | 18 | 130 | 24 | 32 | k:Bacteria |
| OTU_36 | 7 | 247 | 259 | 15 | 3 | 42 | 1 | 101 | 1 | 1143 | 2 | 51 | k:Bacteria,p:Firmicutes,c:Clostridia,o:Clostridiales,f:Lachnospiraceae,g:Anaerostipes |
| OTU_37 | 365 | 200 | 15 | 324 | 105 | 375 | 15 | 67 | 44 | 120 | 108 | 21 | k:Bacteria,p:Bacteroidetes,c:Bacteroidia,o:Bacteroidales,f:Porphyromonadaceae |
| OTU_38 | 288 | 159 | 195 | 123 | 284 | 85 | 76 | 258 | 170 | 156 | 317 | 194 | k:Bacteria,p:Bacteroidetes,c:Bacteroidia,o:Bacteroidales,f:Porphyromonadaceae |
| OTU_39 | 163 | 117 | 43 | 85 | 549 | 34 | 131 | 64 | 218 | 381 | 190 | 150 | k:Bacteria,p:Bacteroidetes,c:Bacteroidia,o:Bacteroidales,f:Porphyromonadaceae |
| OTU_40 | 30 | 12 | 1 | 64 | 196 | 14 | 67 | 18 | 146 | 37 | 714 | 349 | k:Bacteria,p:Bacteroidetes,c:Bacteroidia,o:Bacteroidales |
| OTU_41 | 688 | 256 | 369 | 162 | 531 | 152 | 159 | 335 | 153 | 21 | 180 | 302 | k:Bacteria,p:Bacteroidetes,c:Bacteroidia,o:Bacteroidales,f:Porphyromonadaceae |
| OTU_42 | 542 | 178 | 290 | 49 | 438 | 131 | 52 | 115 | 59 | 90 | 85 | 231 | k:Bacteria,p:Bacteroidetes,c:Bacteroidia,o:Bacteroidales,f:Porphyromonadaceae |
| OTU_43 | 197 | 112 | 49 | 118 | 218 | 71 | 111 | 423 | 254 | 303 | 167 | 103 | k:Bacteria,p:Bacteroidetes,c:Bacteroidia,o:Bacteroidales |
| OTU_44 | 506 | 414 | 104 | 498 | 72 | 211 | 0 | 0 | 0 | 8 | 0 | 0 | k:Bacteria,p:Firmicutes,c:Clostridia,o:Clostridiales,f:Ruminococcaceae,g:Ruminococcus |
| OTU_45 | 7 | 94 | 9 | 37 | 16 | 324 | 235 | 0 | 10 | 1252 | 5 | 0 | k:Bacteria,p:Firmicutes,c:Clostridia,o:Clostridiales,f:Ruminococcaceae,g:Ruminococcus |
| OTU_46 | 62 | 254 | 5 | 243 | 17 | 37 | 5 | 3 | 9 | 472 | 642 | 411 | k:Bacteria,p:Firmicutes,c:Clostridia,o:Clostridiales |
| OTU_47 | 283 | 366 | 84 | 788 | 540 | 749 | 944 | 1150 | 952 | 696 | 1186 | 940 | k:Bacteria,p:Firmicutes,c:Clostridia,o:Clostridiales,f:Ruminococcaceae |
| OTU_48 | 22 | 34 | 437 | 89 | 84 | 122 | 235 | 240 | 286 | 220 | 174 | 8 | k:Bacteria,p:Firmicutes,c:Clostridia,o:Clostridiales,f:Lachnospiraceae |
| OTU_49 | 9 | 5 | 100 | 93 | 91 | 25 | 278 | 602 | 72 | 0 | 319 | 4 | k:Bacteria,p:Firmicutes,c:Clostridia,o:Clostridiales,f:Lachnospiraceae |
| OTU_50 | 336 | 310 | 165 | 246 | 200 | 266 | 776 | 777 | 343 | 391 | 297 | 581 | k:Bacteria,p:Firmicutes,c:Clostridia,o:Clostridiales,f:Ruminococcaceae,g:Intestinimonas,s:Intestinimonas_butyriciproducens |
| OTU_51 | 42 | 189 | 94 | 336 | 236 | 83 | 541 | 416 | 623 | 265 | 1170 | 785 | k:Bacteria,p:Firmicutes,c:Clostridia,o:Clostridiales,f:Ruminococcaceae,g:Oscillibacter |
| OTU_52 | 34 | 44 | 48 | 8 | 26 | 1503 | 14 | 67 | 2 | 5 | 13 | 1 | k:Bacteria |
| OTU_53 | 393 | 95 | 1170 | 21 | 63 | 52 | 112 | 668 | 39 | 47 | 33 | 111 | k:Bacteria,p:Firmicutes,c:Clostridia,o:Clostridiales,f:Lachnospiraceae |
| OTU_54 | 315 | 485 | 13 | 1056 | 158 | 69 | 25 | 108 | 21 | 298 | 19 | 507 | k:Bacteria,p:Firmicutes,c:Clostridia,o:Clostridiales,f:Lachnospiraceae |
| OTU_55 | 106 | 431 | 374 | 10 | 12 | 744 | 331 | 363 | 379 | 810 | 1034 | 700 | k:Bacteria,p:Firmicutes,c:Clostridia,o:Clostridiales,f:Ruminococcaceae |
| OTU_56 | 37 | 10 | 17 | 1 | 2 | 35 | 312 | 815 | 77 | 28 | 21 | 114 | k:Bacteria,p:Firmicutes,c:Clostridia,o:Clostridiales,f:Ruminococcaceae,g:Ruminococcus |
| OTU_57 | 94 | 211 | 67 | 637 | 114 | 220 | 309 | 252 | 325 | 388 | 359 | 1339 | k:Bacteria,p:Firmicutes,c:Clostridia,o:Clostridiales,f:Ruminococcaceae |
| OTU_58 | 3 | 2 | 1116 | 11 | 615 | 106 | 7 | 4 | 5 | 11 | 0 | 6 | k:Bacteria,p:Firmicutes,c:Bacilli,o:Lactobacillales |
| OTU_59 | 202 | 527 | 294 | 158 | 123 | 195 | 157 | 38 | 91 | 330 | 72 | 475 | k:Bacteria,p:Firmicutes,c:Clostridia,o:Clostridiales,f:Ruminococcaceae,g:Clostridium_IV,s:Clostridium_leptum |
| OTU_60 | 106 | 96 | 54 | 147 | 69 | 93 | 106 | 383 | 75 | 162 | 87 | 162 | k:Bacteria,p:Bacteroidetes,c:Bacteroidia,o:Bacteroidales |
| OTU_61 | 18 | 91 | 10 | 3 | 57 | 86 | 505 | 295 | 52 | 77 | 56 | 241 | k:Bacteria,p:Firmicutes,c:Clostridia,o:Clostridiales |
| OTU_62 | 16 | 56 | 19 | 197 | 284 | 116 | 378 | 153 | 273 | 123 | 514 | 388 | k:Bacteria,p:Firmicutes,c:Clostridia,o:Clostridiales,f:Ruminococcaceae |
| OTU_63 | 127 | 22 | 29 | 40 | 716 | 69 | 82 | 85 | 22 | 29 | 6 | 17 | k:Bacteria,p:Bacteroidetes,c:Bacteroidia,o:Bacteroidales,f:Prevotellaceae |
| OTU_64 | 102 | 44 | 58 | 258 | 247 | 107 | 84 | 86 | 265 | 164 | 378 | 141 | k:Bacteria,p:Bacteroidetes,c:Bacteroidia,o:Bacteroidales,f:Porphyromonadaceae |
| OTU_65 | 52 | 131 | 56 | 18 | 62 | 221 | 76 | 306 | 38 | 285 | 66 | 121 | k:Bacteria,p:Firmicutes |
| OTU_66 | 81 | 50 | 65 | 190 | 91 | 252 | 73 | 160 | 70 | 56 | 7 | 72 | k:Bacteria,p:Firmicutes,c:Erysipelotrichia,o:Erysipelotrichales,f:Erysipelotrichaceae |
| OTU_67 | 33 | 10 | 19 | 73 | 141 | 23 | 214 | 411 | 346 | 264 | 804 | 461 | k:Bacteria,p:Bacteroidetes,c:Bacteroidia,o:Bacteroidales,f:Porphyromonadaceae |
| OTU_68 | 44 | 56 | 67 | 102 | 148 | 9 | 60 | 92 | 67 | 178 | 157 | 152 | k:Bacteria,p:Bacteroidetes,c:Bacteroidia,o:Bacteroidales,f:Porphyromonadaceae |
| OTU_69 | 1541 | 633 | 138 | 55 | 452 | 32 | 21 | 19 | 100 | 45 | 233 | 178 | k:Bacteria,p:Bacteroidetes |
| OTU_70 | 1849 | 6 | 18 | 29 | 10 | 385 | 84 | 11 | 19 | 3 | 0 | 105 | k:Bacteria,p:Firmicutes,c:Clostridia,o:Clostridiales,f:Lachnospiraceae |
| OTU_71 | 76 | 85 | 19 | 369 | 261 | 133 | 653 | 271 | 631 | 199 | 1587 | 1018 | k:Bacteria,p:Firmicutes,c:Clostridia,o:Clostridiales,f:Ruminococcaceae,g:Oscillibacter,s:Oscillibacter_valericigenes |
| OTU_72 | 10 | 74 | 1 | 41 | 89 | 60 | 256 | 179 | 136 | 131 | 17 | 798 | k:Bacteria,p:Firmicutes,c:Clostridia,o:Clostridiales |
| OTU_73 | 328 | 223 | 105 | 21 | 62 | 18 | 86 | 193 | 77 | 37 | 30 | 79 | k:Bacteria,p:Bacteroidetes,c:Bacteroidia,o:Bacteroidales,f:Porphyromonadaceae |
| OTU_74 | 0 | 1 | 593 | 2 | 326 | 57 | 3 | 4 | 1 | 3 | 0 | 1 | k:Bacteria,p:Firmicutes,c:Bacilli,o:Lactobacillales,f:Streptococcaceae,g:Lactococcus,s:Lactococcus_taiwanensis |
| OTU_75 | 339 | 63 | 442 | 55 | 62 | 234 | 110 | 123 | 60 | 143 | 85 | 365 | k:Bacteria,p:Firmicutes,c:Clostridia,o:Clostridiales |
| OTU_76 | 484 | 773 | 179 | 15 | 185 | 14 | 11 | 22 | 17 | 22 | 34 | 35 | k:Bacteria,p:Bacteroidetes,c:Bacteroidia,o:Bacteroidales,f:Porphyromonadaceae |
| OTU_77 | 18 | 68 | 7 | 259 | 2 | 336 | 69 | 28 | 51 | 24 | 1 | 9 | k:Bacteria,p:Actinobacteria,c:Actinobacteria,o:Coriobacteriales,f:Coriobacteriaceae |
| OTU_78 | 77 | 3 | 11 | 180 | 78 | 299 | 95 | 19 | 82 | 0 | 153 | 12 | k:Bacteria,p:Firmicutes,c:Clostridia,o:Clostridiales,f:Lachnospiraceae |
| OTU_79 | 144 | 94 | 29 | 99 | 346 | 111 | 72 | 207 | 149 | 68 | 219 | 219 | k:Bacteria,p:Bacteroidetes,c:Bacteroidia,o:Bacteroidales,f:Porphyromonadaceae |
| OTU_80 | 175 | 523 | 39 | 81 | 76 | 117 | 3 | 0 | 164 | 0 | 3 | 6 | k:Bacteria |
| OTU_81 | 15 | 10 | 2 | 58 | 63 | 44 | 170 | 116 | 199 | 148 | 257 | 192 | k:Bacteria,p:Firmicutes,c:Clostridia,o:Clostridiales,f:Ruminococcaceae |
| OTU_82 | 110 | 80 | 62 | 206 | 71 | 170 | 138 | 228 | 155 | 49 | 112 | 144 | k:Bacteria,p:Firmicutes,c:Clostridia,o:Clostridiales,f:Ruminococcaceae |
| OTU_83 | 6 | 31 | 71 | 1 | 0 | 3 | 0 | 795 | 0 | 0 | 10 | 2 | k:Bacteria,p:Firmicutes,c:Clostridia,o:Clostridiales,f:Lachnospiraceae |
| OTU_84 | 24 | 0 | 1 | 22 | 0 | 3 | 1 | 42 | 2 | 27 | 331 | 385 | k:Bacteria,p:Firmicutes,c:Clostridia,o:Clostridiales,f:Lachnospiraceae |
| OTU_85 | 7 | 77 | 14 | 39 | 669 | 15 | 47 | 87 | 35 | 0 | 37 | 11 | k:Bacteria,p:Bacteroidetes,c:Bacteroidia,o:Bacteroidales,f:Porphyromonadaceae |
| OTU_86 | 273 | 17 | 73 | 2 | 1 | 240 | 20 | 24 | 35 | 4 | 67 | 43 | k:Bacteria |
| OTU_87 | 43 | 17 | 4 | 158 | 7 | 40 | 56 | 62 | 75 | 289 | 24 | 156 | k:Bacteria,p:Firmicutes,c:Erysipelotrichia,o:Erysipelotrichales,f:Erysipelotrichaceae,g:Allobaculum,s:Allobaculum_stercoricanis |
| OTU_88 | 125 | 62 | 56 | 101 | 324 | 25 | 612 | 402 | 313 | 15 | 172 | 51 | k:Bacteria,p:Bacteroidetes,c:Bacteroidia,o:Bacteroidales,f:Porphyromonadaceae |
| OTU_89 | 55 | 10 | 22 | 65 | 338 | 69 | 41 | 132 | 77 | 59 | 145 | 100 | k:Bacteria,p:Bacteroidetes,c:Bacteroidia,o:Bacteroidales,f:Porphyromonadaceae |
| OTU_90 | 2 | 94 | 39 | 99 | 303 | 33 | 33 | 32 | 23 | 35 | 76 | 10 | k:Bacteria,p:Firmicutes,c:Negativicutes,o:Selenomonadales,f:Veillonellaceae |
| OTU_91 | 248 | 157 | 8 | 27 | 557 | 5 | 210 | 192 | 11 | 19 | 38 | 8 | k:Bacteria,p:Bacteroidetes,c:Bacteroidia,o:Bacteroidales,f:Porphyromonadaceae |
| OTU_92 | 12 | 4 | 15 | 106 | 80 | 57 | 135 | 133 | 141 | 43 | 84 | 68 | k:Bacteria,p:Firmicutes |
| OTU_93 | 234 | 38 | 6 | 29 | 299 | 7 | 51 | 62 | 65 | 14 | 58 | 59 | k:Bacteria,p:Bacteroidetes,c:Bacteroidia,o:Bacteroidales,f:Porphyromonadaceae |
| OTU_94 | 15 | 25 | 1 | 392 | 31 | 260 | 2 | 5 | 9 | 15 | 16 | 27 | k:Bacteria,p:Firmicutes,c:Clostridia,o:Clostridiales,f:Ruminococcaceae,g:Clostridium_IV |
| OTU_95 | 1 | 1 | 0 | 10 | 4 | 44 | 134 | 2 | 1 | 1 | 1 | 474 | k:Bacteria |
| OTU_96 | 671 | 141 | 40 | 462 | 122 | 332 | 286 | 126 | 181 | 255 | 106 | 319 | k:Bacteria,p:Firmicutes,c:Clostridia,o:Clostridiales,f:Lachnospiraceae |
| OTU_97 | 1 | 1 | 390 | 6 | 234 | 34 | 5 | 2 | 1 | 6 | 0 | 1 | k:Bacteria,p:Firmicutes,c:Bacilli,o:Bacillales,f:Bacillales_Incertae_Sedis_XII,g:Exiguobacterium,s:Exiguobacterium_mexicanum |
| OTU_98 | 0 | 7 | 20 | 1 | 9 | 4 | 238 | 269 | 137 | 0 | 0 | 23 | k:Bacteria,p:Firmicutes,c:Clostridia,o:Clostridiales,f:Ruminococcaceae,g:Ruminococcus |
| OTU_99 | 246 | 113 | 144 | 18 | 158 | 55 | 12 | 74 | 7 | 37 | 18 | 13 | k:Bacteria,p:Bacteroidetes,c:Bacteroidia,o:Bacteroidales,f:Porphyromonadaceae |
| OTU_100 | 49 | 36 | 23 | 13 | 307 | 8 | 25 | 58 | 23 | 17 | 44 | 19 | k:Bacteria,p:Bacteroidetes,c:Bacteroidia,o:Bacteroidales,f:Porphyromonadaceae |
| OTU_101 | 9 | 37 | 24 | 219 | 57 | 43 | 34 | 49 | 56 | 81 | 19 | 43 | k:Bacteria,p:Proteobacteria,c:Deltaproteobacteria,o:Desulfovibrionales,f:Desulfovibrionaceae,g:Desulfovibrio |
| OTU_102 | 116 | 15 | 31 | 35 | 65 | 27 | 32 | 105 | 53 | 38 | 80 | 80 | k:Bacteria,p:Bacteroidetes,c:Bacteroidia,o:Bacteroidales |
| OTU_103 | 242 | 27 | 232 | 6 | 83 | 6 | 5 | 31 | 3 | 449 | 0 | 6 | k:Bacteria,p:Firmicutes,c:Clostridia,o:Clostridiales,f:Ruminococcaceae |
| OTU_104 | 189 | 135 | 42 | 17 | 32 | 17 | 54 | 7 | 41 | 27 | 39 | 36 | k:Bacteria,p:Firmicutes,c:Clostridia,o:Clostridiales,f:Ruminococcaceae |
| OTU_105 | 0 | 1 | 426 | 1 | 254 | 45 | 3 | 1 | 2 | 3 | 0 | 0 | k:Bacteria,p:Firmicutes,c:Bacilli,o:Bacillales,f:Paenibacillaceae_1,g:Paenibacillus |
| OTU_106 | 220 | 14 | 2 | 26 | 185 | 17 | 28 | 172 | 27 | 15 | 4 | 25 | k:Bacteria,p:Bacteroidetes,c:Bacteroidia,o:Bacteroidales,f:Porphyromonadaceae |
| OTU_107 | 70 | 28 | 37 | 18 | 109 | 37 | 33 | 65 | 65 | 68 | 35 | 75 | k:Bacteria,p:Bacteroidetes,c:Bacteroidia,o:Bacteroidales,f:Porphyromonadaceae |
| OTU_108 | 32 | 37 | 28 | 116 | 53 | 77 | 143 | 55 | 58 | 14 | 25 | 135 | k:Bacteria,p:Firmicutes,c:Clostridia,o:Clostridiales |
| OTU_109 | 3 | 9 | 0 | 33 | 59 | 145 | 35 | 63 | 64 | 36 | 278 | 43 | k:Bacteria,p:Firmicutes,c:Clostridia,o:Clostridiales |
| OTU_110 | 66 | 93 | 191 | 22 | 9 | 433 | 23 | 68 | 19 | 8 | 33 | 59 | k:Bacteria,p:Firmicutes,c:Clostridia,o:Clostridiales |
| OTU_111 | 119 | 16 | 14 | 4 | 240 | 2 | 40 | 24 | 32 | 5 | 88 | 73 | k:Bacteria,p:Bacteroidetes,c:Bacteroidia,o:Bacteroidales,f:Bacteroidaceae,g:Bacteroides |
| OTU_112 | 3 | 5 | 1 | 23 | 17 | 0 | 251 | 244 | 0 | 59 | 0 | 23 | k:Bacteria,p:Firmicutes,c:Clostridia,o:Clostridiales |
| OTU_113 | 2 | 1 | 389 | 1 | 195 | 45 | 5 | 1 | 1 | 2 | 0 | 5 | k:Bacteria,p:Firmicutes,c:Bacilli,o:Lactobacillales,f:Streptococcaceae,g:Streptococcus |
| OTU_114 | 0 | 1 | 0 | 4 | 0 | 7 | 51 | 0 | 43 | 0 | 439 | 0 | k:Bacteria,p:Bacteroidetes,c:Bacteroidia,o:Bacteroidales |
| OTU_115 | 5 | 32 | 31 | 35 | 434 | 40 | 26 | 42 | 21 | 9 | 58 | 84 | k:Bacteria,p:Firmicutes,c:Clostridia,o:Clostridiales,f:Lachnospiraceae |
| OTU_116 | 122 | 137 | 42 | 62 | 6 | 498 | 5 | 3 | 23 | 13 | 291 | 53 | k:Bacteria,p:Firmicutes,c:Clostridia,o:Clostridiales |
| OTU_117 | 15 | 56 | 1 | 118 | 31 | 174 | 45 | 39 | 27 | 44 | 17 | 59 | k:Bacteria,p:Actinobacteria,c:Actinobacteria,o:Coriobacteriales,f:Coriobacteriaceae,g:Asaccharobacter,s:Asaccharobacter_celatus |
| OTU_118 | 44 | 57 | 38 | 39 | 9 | 46 | 164 | 138 | 45 | 298 | 88 | 188 | k:Bacteria,p:Firmicutes,c:Clostridia,o:Clostridiales,f:Lachnospiraceae |
| OTU_119 | 18 | 13 | 43 | 15 | 8 | 144 | 22 | 42 | 23 | 83 | 56 | 76 | k:Bacteria,p:Firmicutes |
| OTU_120 | 181 | 66 | 55 | 11 | 50 | 4 | 24 | 75 | 29 | 10 | 22 | 8 | k:Bacteria,p:Bacteroidetes,c:Bacteroidia,o:Bacteroidales,f:Porphyromonadaceae |
| OTU_121 | 26 | 34 | 62 | 58 | 49 | 59 | 49 | 53 | 33 | 77 | 49 | 98 | k:Bacteria,p:Firmicutes,c:Clostridia,o:Clostridiales,f:Ruminococcaceae |
| OTU_122 | 45 | 14 | 6 | 0 | 3 | 104 | 41 | 23 | 47 | 78 | 59 | 144 | k:Bacteria,p:Firmicutes,c:Clostridia,o:Clostridiales,f:Ruminococcaceae |
| OTU_123 | 24 | 8 | 5 | 8 | 1 | 202 | 42 | 30 | 113 | 103 | 19 | 624 | k:Bacteria,p:Firmicutes,c:Clostridia,o:Clostridiales |
| OTU_124 | 2 | 0 | 40 | 266 | 11 | 69 | 11 | 3 | 2 | 36 | 4 | 39 | k:Bacteria,p:Verrucomicrobia,c:Verrucomicrobiae,o:Verrucomicrobiales,f:Verrucomicrobiaceae,g:Akkermansia,s:Akkermansia_muciniphila |
| OTU_125 | 97 | 172 | 157 | 6 | 17 | 98 | 133 | 44 | 95 | 136 | 137 | 32 | k:Bacteria,p:Firmicutes,c:Clostridia,o:Clostridiales |
| OTU_126 | 44 | 85 | 0 | 65 | 82 | 219 | 0 | 0 | 1 | 0 | 27 | 9 | k:Bacteria,p:Firmicutes,c:Clostridia,o:Clostridiales |
| OTU_127 | 0 | 0 | 314 | 4 | 156 | 46 | 2 | 4 | 0 | 8 | 0 | 2 | k:Bacteria,p:Firmicutes,c:Bacilli,o:Lactobacillales,f:Aerococcaceae,g:Aerococcus,s:Aerococcus_urinaeequi |
| OTU_128 | 0 | 6 | 612 | 1 | 0 | 0 | 2 | 10 | 1 | 0 | 1 | 0 | k:Bacteria,p:Firmicutes,c:Clostridia,o:Clostridiales,f:Lachnospiraceae |
| OTU_129 | 43 | 69 | 471 | 0 | 3 | 3 | 109 | 11 | 14 | 3 | 0 | 1 | k:Bacteria,p:Firmicutes,c:Clostridia,o:Clostridiales,f:Lachnospiraceae |
| OTU_130 | 11 | 245 | 12 | 27 | 8 | 121 | 22 | 193 | 3 | 0 | 8 | 14 | k:Bacteria |
| OTU_131 | 103 | 152 | 170 | 9 | 107 | 123 | 135 | 43 | 9 | 58 | 200 | 75 | k:Bacteria,p:Firmicutes,c:Clostridia,o:Clostridiales |
| OTU_132 | 14 | 13 | 11 | 59 | 1 | 28 | 138 | 265 | 25 | 22 | 7 | 71 | k:Bacteria,p:Firmicutes,c:Clostridia,o:Clostridiales,f:Lachnospiraceae,g:Clostridium_XlVa,s:Clostridium_scindens |
| OTU_133 | 13 | 4 | 1 | 33 | 225 | 18 | 28 | 15 | 61 | 63 | 227 | 60 | k:Bacteria,p:Bacteroidetes,c:Bacteroidia,o:Bacteroidales |
| OTU_134 | 22 | 11 | 45 | 25 | 9 | 37 | 12 | 23 | 119 | 173 | 9 | 165 | k:Bacteria,p:Firmicutes,c:Clostridia,o:Clostridiales,f:Lachnospiraceae |
| OTU_135 | 18 | 201 | 143 | 108 | 15 | 40 | 12 | 159 | 1 | 89 | 1 | 3 | k:Bacteria,p:Firmicutes,c:Clostridia,o:Clostridiales,f:Lachnospiraceae |
| OTU_136 | 0 | 0 | 262 | 4 | 132 | 22 | 5 | 3 | 0 | 5 | 0 | 4 | k:Bacteria,p:Firmicutes,c:Bacilli,o:Bacillales,f:Bacillaceae_1,g:Bacillus,s:Bacillus_safensis |
| OTU_137 | 41 | 23 | 36 | 49 | 38 | 67 | 154 | 92 | 101 | 222 | 110 | 132 | k:Bacteria,p:Firmicutes,c:Clostridia,o:Clostridiales,f:Ruminococcaceae |
| OTU_138 | 41 | 11 | 14 | 64 | 113 | 28 | 86 | 45 | 41 | 49 | 92 | 92 | k:Bacteria,p:Firmicutes,c:Clostridia,o:Clostridiales,f:Lachnospiraceae,g:Clostridium_XlVb,s:Clostridium_lactatifermentans |
| OTU_139 | 12 | 120 | 14 | 435 | 3 | 28 | 109 | 69 | 29 | 154 | 68 | 9 | k:Bacteria,p:Firmicutes,c:Clostridia,o:Clostridiales,f:Lachnospiraceae |
| OTU_140 | 37 | 23 | 326 | 11 | 35 | 70 | 21 | 0 | 2 | 27 | 2 | 672 | k:Bacteria |
| OTU_141 | 0 | 0 | 266 | 1 | 117 | 29 | 3 | 0 | 1 | 3 | 0 | 0 | k:Bacteria,p:Firmicutes,c:Bacilli,o:Bacillales,f:Bacillaceae_2 |
| OTU_142 | 2 | 0 | 3 | 22 | 2 | 11 | 2 | 3 | 4 | 392 | 10 | 22 | k:Bacteria,p:Proteobacteria,c:Gammaproteobacteria,o:Enterobacteriales,f:Enterobacteriaceae,g:Escherichia/Shigella,s:Escherichia_fergusonii |
| OTU_143 | 31 | 48 | 36 | 12 | 57 | 20 | 19 | 46 | 54 | 30 | 45 | 16 | k:Bacteria,p:Bacteroidetes,c:Bacteroidia,o:Bacteroidales |
| OTU_144 | 260 | 140 | 87 | 25 | 177 | 25 | 4 | 26 | 11 | 5 | 18 | 18 | k:Bacteria,p:Bacteroidetes,c:Bacteroidia,o:Bacteroidales,f:Porphyromonadaceae |
| OTU_145 | 0 | 0 | 294 | 4 | 151 | 22 | 2 | 1 | 0 | 1 | 0 | 2 | k:Bacteria,p:Firmicutes,c:Bacilli,o:Lactobacillales,f:Leuconostocaceae,g:Leuconostoc |
| OTU_146 | 140 | 47 | 5 | 1 | 54 | 7 | 75 | 2 | 26 | 5 | 64 | 33 | k:Bacteria,p:Bacteroidetes,c:Bacteroidia,o:Bacteroidales,f:Porphyromonadaceae |
| OTU_147 | 16 | 7 | 10 | 232 | 17 | 14 | 59 | 108 | 2 | 50 | 14 | 29 | k:Bacteria,p:Firmicutes,c:Clostridia,o:Clostridiales,f:Lachnospiraceae |
| OTU_148 | 143 | 38 | 19 | 3 | 24 | 2 | 25 | 163 | 24 | 2 | 58 | 61 | k:Bacteria,p:Bacteroidetes,c:Bacteroidia,o:Bacteroidales,f:Porphyromonadaceae |
| OTU_149 | 403 | 1 | 4 | 0 | 7 | 57 | 0 | 0 | 0 | 0 | 68 | 0 | k:Bacteria,p:Firmicutes,c:Clostridia,o:Clostridiales,f:Lachnospiraceae,g:Clostridium_XlVa |
| OTU_150 | 1 | 357 | 15 | 1 | 25 | 0 | 0 | 0 | 0 | 0 | 0 | 0 | k:Bacteria,p:Firmicutes,c:Bacilli,o:Lactobacillales,f:Lactobacillaceae,g:Lactobacillus |
| OTU_151 | 17 | 57 | 25 | 4 | 9 | 36 | 41 | 144 | 17 | 11 | 22 | 3 | k:Bacteria |
| OTU_152 | 69 | 59 | 16 | 16 | 124 | 39 | 36 | 20 | 19 | 2 | 94 | 7 | k:Bacteria,p:Bacteroidetes,c:Bacteroidia,o:Bacteroidales,f:Porphyromonadaceae |
| OTU_153 | 16 | 18 | 12 | 118 | 42 | 59 | 144 | 98 | 28 | 29 | 5 | 6 | k:Bacteria |
| OTU_154 | 1 | 2 | 6 | 12 | 5 | 10 | 66 | 231 | 26 | 18 | 10 | 24 | k:Bacteria,p:Firmicutes,c:Clostridia,o:Clostridiales,f:Lachnospiraceae |
| OTU_155 | 14 | 29 | 12 | 37 | 10 | 20 | 42 | 57 | 41 | 34 | 39 | 53 | k:Bacteria,p:Firmicutes,c:Clostridia,o:Clostridiales,f:Ruminococcaceae |
| OTU_156 | 0 | 1 | 51 | 0 | 0 | 2 | 111 | 115 | 33 | 87 | 0 | 31 | k:Bacteria |
| OTU_157 | 190 | 605 | 395 | 206 | 58 | 40 | 67 | 70 | 38 | 266 | 11 | 38 | k:Bacteria,p:Firmicutes,c:Clostridia,o:Clostridiales,f:Lachnospiraceae |
| OTU_158 | 4 | 15 | 2 | 19 | 3 | 153 | 34 | 0 | 108 | 1 | 6 | 106 | k:Bacteria,p:Firmicutes,c:Clostridia,o:Clostridiales |
| OTU_159 | 41 | 28 | 30 | 242 | 22 | 37 | 68 | 82 | 39 | 93 | 38 | 178 | k:Bacteria,p:Firmicutes,c:Clostridia,o:Clostridiales,f:Ruminococcaceae |
| OTU_160 | 12 | 0 | 0 | 13 | 127 | 128 | 20 | 0 | 0 | 0 | 0 | 55 | k:Bacteria,p:Proteobacteria,c:Alphaproteobacteria |
| OTU_161 | 35 | 48 | 8 | 94 | 137 | 33 | 7 | 7 | 15 | 56 | 27 | 103 | k:Bacteria,p:Bacteroidetes,c:Bacteroidia,o:Bacteroidales,f:Porphyromonadaceae |
| OTU_162 | 23 | 94 | 74 | 93 | 45 | 61 | 156 | 237 | 55 | 25 | 22 | 101 | k:Bacteria,p:Firmicutes,c:Clostridia,o:Clostridiales,f:Lachnospiraceae |
| OTU_163 | 49 | 79 | 69 | 78 | 48 | 147 | 203 | 118 | 67 | 100 | 44 | 90 | k:Bacteria,p:Firmicutes,c:Clostridia,o:Clostridiales,f:Lachnospiraceae |
| OTU_164 | 5 | 18 | 54 | 23 | 39 | 96 | 36 | 21 | 26 | 48 | 20 | 63 | k:Bacteria,p:Firmicutes,c:Clostridia,o:Clostridiales,f:Clostridiaceae_1,g:Clostridium_sensu_stricto |
| OTU_165 | 85 | 148 | 143 | 0 | 2 | 0 | 7 | 1 | 28 | 0 | 22 | 36 | k:Bacteria,p:Bacteroidetes,c:Bacteroidia,o:Bacteroidales,f:Prevotellaceae,g:Prevotella |
| OTU_166 | 62 | 17 | 23 | 1 | 110 | 4 | 20 | 10 | 30 | 3 | 31 | 11 | k:Bacteria,p:Bacteroidetes,c:Bacteroidia,o:Bacteroidales,f:Porphyromonadaceae |
| OTU_167 | 16 | 13 | 23 | 39 | 19 | 54 | 97 | 108 | 63 | 99 | 168 | 93 | k:Bacteria,p:Firmicutes,c:Clostridia,o:Clostridiales |
| OTU_168 | 34 | 18 | 26 | 45 | 27 | 44 | 26 | 5 | 15 | 75 | 32 | 45 | k:Bacteria,p:Firmicutes,c:Clostridia,o:Clostridiales |
| OTU_169 | 24 | 114 | 6 | 60 | 8 | 59 | 59 | 147 | 100 | 61 | 42 | 187 | k:Bacteria,p:Firmicutes,c:Clostridia,o:Clostridiales,f:Ruminococcaceae |
| OTU_170 | 38 | 462 | 71 | 0 | 4 | 4 | 0 | 21 | 3 | 10 | 0 | 5 | k:Bacteria,p:Firmicutes,c:Bacilli,o:Lactobacillales,f:Lactobacillaceae,g:Lactobacillus |
| OTU_171 | 0 | 1 | 1 | 0 | 0 | 0 | 0 | 296 | 0 | 0 | 0 | 0 | k:Bacteria |
| OTU_172 | 1 | 3 | 6 | 25 | 49 | 9 | 63 | 66 | 60 | 13 | 21 | 5 | k:Bacteria,p:Proteobacteria,c:Deltaproteobacteria,o:Desulfovibrionales |
| OTU_173 | 32 | 7 | 15 | 19 | 117 | 8 | 7 | 53 | 28 | 4 | 19 | 20 | k:Bacteria,p:Bacteroidetes,c:Bacteroidia,o:Bacteroidales,f:Rikenellaceae,g:Alistipes |
| OTU_174 | 2 | 13 | 6 | 80 | 37 | 29 | 33 | 68 | 39 | 23 | 15 | 19 | k:Bacteria,p:Firmicutes,c:Clostridia,o:Clostridiales,f:Ruminococcaceae,g:Oscillibacter,s:Oscillibacter_valericigenes |
| OTU_175 | 70 | 0 | 2 | 0 | 2 | 217 | 0 | 0 | 0 | 0 | 0 | 0 | k:Bacteria,p:Firmicutes,c:Clostridia,o:Clostridiales,f:Ruminococcaceae |
| OTU_176 | 11 | 8 | 41 | 8 | 19 | 25 | 23 | 50 | 12 | 58 | 21 | 32 | k:Bacteria,p:Firmicutes |
| OTU_177 | 32 | 38 | 2 | 143 | 79 | 32 | 127 | 43 | 32 | 71 | 28 | 57 | k:Bacteria,p:Firmicutes,c:Clostridia,o:Clostridiales,f:Lachnospiraceae |
| OTU_178 | 33 | 56 | 50 | 158 | 95 | 42 | 47 | 34 | 47 | 21 | 61 | 53 | k:Bacteria,p:Firmicutes,c:Clostridia,o:Clostridiales,f:Lachnospiraceae |
| OTU_179 | 52 | 44 | 129 | 99 | 40 | 69 | 64 | 56 | 14 | 18 | 50 | 108 | k:Bacteria,p:Firmicutes,c:Clostridia,o:Clostridiales,f:Lachnospiraceae |
| OTU_180 | 21 | 8 | 17 | 42 | 11 | 24 | 51 | 58 | 18 | 15 | 6 | 26 | k:Bacteria,p:Firmicutes,c:Clostridia,o:Clostridiales,f:Lachnospiraceae |
| OTU_181 | 5 | 4 | 1 | 2 | 115 | 2 | 58 | 9 | 41 | 3 | 13 | 38 | k:Bacteria,p:Bacteroidetes,c:Bacteroidia,o:Bacteroidales,f:Porphyromonadaceae |
| OTU_182 | 111 | 53 | 0 | 72 | 18 | 18 | 0 | 1 | 0 | 1 | 7 | 2 | k:Bacteria |
| OTU_183 | 4 | 6 | 20 | 26 | 12 | 33 | 89 | 68 | 11 | 31 | 29 | 27 | k:Bacteria,p:Firmicutes,c:Clostridia,o:Clostridiales,f:Ruminococcaceae |
| OTU_184 | 24 | 3 | 2 | 14 | 231 | 4 | 0 | 9 | 0 | 0 | 2 | 1 | k:Bacteria,p:Bacteroidetes,c:Bacteroidia,o:Bacteroidales,f:Porphyromonadaceae |
| OTU_185 | 14 | 20 | 176 | 37 | 6 | 36 | 36 | 0 | 13 | 171 | 26 | 2 | k:Bacteria,p:Firmicutes,c:Clostridia,o:Clostridiales,f:Eubacteriaceae,g:Eubacterium,s:Eubacterium_coprostanoligenes |
| OTU_186 | 81 | 161 | 54 | 3 | 3 | 8 | 3 | 14 | 7 | 2 | 12 | 125 | k:Bacteria,p:Firmicutes,c:Clostridia,o:Clostridiales,f:Lachnospiraceae |
| OTU_187 | 0 | 0 | 166 | 1 | 67 | 15 | 1 | 0 | 1 | 1 | 0 | 0 | k:Bacteria,p:Firmicutes,c:Clostridia,o:Clostridiales,f:Clostridiaceae_2,g:Alkaliphilus,s:Alkaliphilus_oremlandii |
| OTU_188 | 7 | 4 | 0 | 57 | 0 | 71 | 0 | 8 | 25 | 1 | 45 | 25 | k:Bacteria,p:Proteobacteria,c:Gammaproteobacteria,o:Pasteurellales,f:Pasteurellaceae |
| OTU_189 | 4 | 43 | 97 | 1 | 173 | 120 | 0 | 0 | 0 | 63 | 33 | 0 | k:Bacteria |
| OTU_190 | 0 | 0 | 0 | 0 | 0 | 0 | 0 | 0 | 0 | 0 | 242 | 0 | k:Bacteria,p:Firmicutes,c:Clostridia,o:Clostridiales |
| OTU_191 | 142 | 99 | 15 | 5 | 76 | 8 | 18 | 18 | 9 | 22 | 46 | 4 | k:Bacteria,p:Bacteroidetes,c:Bacteroidia,o:Bacteroidales,f:Porphyromonadaceae |
| OTU_192 | 140 | 24 | 11 | 2 | 87 | 5 | 11 | 6 | 20 | 0 | 4 | 31 | k:Bacteria,p:Bacteroidetes,c:Bacteroidia,o:Bacteroidales,f:Bacteroidaceae,g:Bacteroides,s:Bacteroides_uniformis |
| OTU_193 | 13 | 21 | 16 | 24 | 15 | 25 | 52 | 46 | 29 | 32 | 18 | 30 | k:Bacteria,p:Firmicutes |
| OTU_194 | 25 | 117 | 115 | 5 | 59 | 35 | 116 | 31 | 42 | 46 | 40 | 36 | k:Bacteria,p:Firmicutes,c:Clostridia |
| OTU_195 | 13 | 4 | 8 | 0 | 0 | 0 | 55 | 49 | 3 | 2 | 106 | 6 | k:Bacteria,p:Bacteroidetes,c:Bacteroidia,o:Bacteroidales,f:Prevotellaceae,g:Prevotella |
| OTU_196 | 41 | 138 | 106 | 2 | 75 | 36 | 54 | 160 | 21 | 12 | 1 | 63 | k:Bacteria,p:Firmicutes,c:Clostridia,o:Clostridiales |
| OTU_197 | 31 | 111 | 11 | 6 | 131 | 75 | 15 | 1 | 1 | 5 | 15 | 0 | k:Bacteria |
| OTU_198 | 13 | 3 | 0 | 67 | 8 | 12 | 44 | 27 | 22 | 8 | 12 | 60 | k:Bacteria,p:Firmicutes,c:Clostridia,o:Clostridiales,f:Ruminococcaceae |
| OTU_199 | 82 | 89 | 0 | 2 | 53 | 62 | 15 | 105 | 1 | 0 | 3 | 27 | k:Bacteria,p:Firmicutes,c:Clostridia,o:Clostridiales |
| OTU_200 | 51 | 39 | 1 | 1 | 86 | 3 | 4 | 7 | 2 | 0 | 2 | 0 | k:Bacteria,p:Bacteroidetes,c:Bacteroidia,o:Bacteroidales,f:Prevotellaceae,g:Prevotella |
| OTU_201 | 5 | 27 | 7 | 122 | 55 | 93 | 75 | 47 | 13 | 19 | 21 | 51 | k:Bacteria,p:Firmicutes,c:Clostridia,o:Clostridiales |
| OTU_202 | 23 | 5 | 10 | 8 | 2 | 5 | 27 | 9 | 12 | 78 | 1 | 50 | k:Bacteria,p:Firmicutes,c:Clostridia,o:Clostridiales,f:Lachnospiraceae |
| OTU_203 | 6 | 24 | 206 | 0 | 8 | 112 | 0 | 2 | 23 | 6 | 27 | 8 | k:Bacteria,p:Firmicutes,c:Clostridia,o:Clostridiales |
| OTU_204 | 0 | 0 | 0 | 0 | 0 | 1 | 1 | 139 | 4 | 1 | 1 | 79 | k:Bacteria,p:Firmicutes,c:Clostridia,o:Clostridiales,f:Lachnospiraceae,g:Anaerostipes |
| OTU_205 | 13 | 7 | 11 | 7 | 11 | 36 | 6 | 16 | 12 | 62 | 14 | 10 | k:Bacteria,p:Firmicutes,c:Clostridia,o:Clostridiales |
| OTU_206 | 0 | 1 | 0 | 0 | 0 | 55 | 10 | 3 | 58 | 44 | 35 | 49 | k:Bacteria,p:Firmicutes,c:Clostridia,o:Clostridiales,f:Ruminococcaceae |
| OTU_207 | 58 | 138 | 46 | 71 | 20 | 7 | 41 | 9 | 2 | 2 | 26 | 5 | k:Bacteria,p:Firmicutes,c:Clostridia,o:Clostridiales,f:Ruminococcaceae,g:Clostridium_IV,s:Eubacterium_siraeum |
| OTU_208 | 26 | 4 | 1 | 8 | 9 | 1 | 20 | 70 | 20 | 10 | 11 | 8 | k:Bacteria,p:Bacteroidetes,c:Bacteroidia,o:Bacteroidales |
| OTU_209 | 4 | 22 | 21 | 22 | 3 | 17 | 21 | 29 | 12 | 12 | 1 | 27 | k:Bacteria,p:Firmicutes,c:Erysipelotrichia,o:Erysipelotrichales,f:Erysipelotrichaceae |
| OTU_210 | 21 | 17 | 20 | 42 | 22 | 16 | 50 | 38 | 22 | 11 | 8 | 28 | k:Bacteria,p:Firmicutes,c:Clostridia,o:Clostridiales,f:Lachnospiraceae |
| OTU_211 | 5 | 111 | 6 | 2 | 11 | 13 | 0 | 0 | 0 | 61 | 0 | 0 | k:Bacteria |
| OTU_212 | 1 | 1 | 3 | 22 | 4 | 16 | 35 | 20 | 24 | 48 | 50 | 40 | k:Bacteria,p:Firmicutes,c:Clostridia,o:Clostridiales,f:Lachnospiraceae |
| OTU_213 | 64 | 33 | 23 | 119 | 48 | 93 | 64 | 59 | 72 | 79 | 89 | 122 | k:Bacteria,p:Firmicutes,c:Clostridia,o:Clostridiales,f:Lachnospiraceae,g:Clostridium_XlVa,s:Clostridium_scindens |
| OTU_214 | 1 | 2 | 200 | 0 | 0 | 0 | 0 | 1 | 0 | 0 | 0 | 0 | k:Bacteria,p:Firmicutes,c:Clostridia,o:Clostridiales,f:Lachnospiraceae |
| OTU_215 | 60 | 85 | 49 | 75 | 71 | 54 | 55 | 158 | 72 | 21 | 75 | 133 | k:Bacteria,p:Firmicutes,c:Clostridia,o:Clostridiales |
| OTU_216 | 13 | 4 | 3 | 0 | 204 | 1 | 7 | 15 | 3 | 4 | 6 | 18 | k:Bacteria,p:Bacteroidetes,c:Bacteroidia,o:Bacteroidales,f:Porphyromonadaceae,g:Parabacteroides,s:Parabacteroides_merdae |
| OTU_217 | 9 | 3 | 7 | 1 | 1 | 30 | 10 | 121 | 27 | 16 | 3 | 1 | k:Bacteria,p:Firmicutes,c:Clostridia,o:Clostridiales,f:Lachnospiraceae |
| OTU_218 | 85 | 12 | 41 | 0 | 90 | 4 | 20 | 10 | 16 | 6 | 51 | 10 | k:Bacteria,p:Bacteroidetes,c:Bacteroidia,o:Bacteroidales,f:Porphyromonadaceae |
| OTU_219 | 0 | 0 | 1 | 1 | 0 | 1 | 36 | 109 | 10 | 33 | 1 | 5 | k:Bacteria,p:Firmicutes,c:Clostridia,o:Clostridiales,f:Lachnospiraceae |
| OTU_220 | 6 | 2 | 3 | 6 | 27 | 52 | 0 | 38 | 3 | 12 | 35 | 21 | k:Bacteria,p:Firmicutes,c:Clostridia,o:Clostridiales,f:Ruminococcaceae |
| OTU_221 | 1 | 10 | 33 | 0 | 16 | 5 | 51 | 6 | 4 | 33 | 27 | 1 | k:Bacteria |
| OTU_222 | 13 | 8 | 18 | 50 | 21 | 44 | 20 | 18 | 6 | 16 | 14 | 33 | k:Bacteria,p:Firmicutes,c:Clostridia,o:Clostridiales |
| OTU_223 | 82 | 9 | 35 | 12 | 3 | 2 | 7 | 10 | 24 | 7 | 11 | 3 | k:Bacteria,p:Firmicutes,c:Clostridia,o:Clostridiales,f:Lachnospiraceae |
| OTU_224 | 17 | 17 | 1 | 18 | 47 | 1 | 23 | 19 | 20 | 7 | 15 | 4 | k:Bacteria,p:Bacteroidetes,c:Bacteroidia,o:Bacteroidales,f:Porphyromonadaceae |
| OTU_225 | 4 | 14 | 17 | 14 | 6 | 10 | 30 | 8 | 30 | 4 | 19 | 46 | k:Bacteria,p:Firmicutes,c:Clostridia,o:Clostridiales,f:Lachnospiraceae,g:Clostridium_XlVa |
| OTU_226 | 12 | 73 | 12 | 18 | 41 | 38 | 60 | 11 | 5 | 2 | 12 | 4 | k:Bacteria |
| OTU_227 | 82 | 39 | 65 | 65 | 34 | 17 | 98 | 74 | 55 | 22 | 25 | 68 | k:Bacteria,p:Firmicutes,c:Clostridia,o:Clostridiales,f:Lachnospiraceae |
| OTU_228 | 14 | 6 | 67 | 5 | 0 | 3 | 47 | 43 | 10 | 115 | 24 | 2 | k:Bacteria,p:Firmicutes,c:Clostridia,o:Clostridiales,f:Lachnospiraceae |
| OTU_229 | 45 | 112 | 25 | 18 | 57 | 155 | 74 | 125 | 83 | 10 | 14 | 90 | k:Bacteria |
| OTU_230 | 7 | 40 | 45 | 29 | 31 | 29 | 5 | 8 | 23 | 19 | 24 | 27 | k:Bacteria,p:Firmicutes,c:Clostridia,o:Clostridiales,f:Lachnospiraceae |
| OTU_231 | 55 | 74 | 152 | 0 | 2 | 10 | 6 | 12 | 0 | 1 | 3 | 5 | k:Bacteria,p:Firmicutes,c:Clostridia,o:Clostridiales,f:Lachnospiraceae |
| OTU_232 | 40 | 59 | 39 | 8 | 1 | 4 | 18 | 172 | 35 | 106 | 10 | 34 | k:Bacteria,p:Firmicutes,c:Clostridia,o:Clostridiales,f:Ruminococcaceae |
| OTU_233 | 20 | 39 | 15 | 18 | 8 | 19 | 27 | 99 | 12 | 32 | 102 | 25 | k:Bacteria,p:Firmicutes,c:Clostridia,o:Clostridiales,f:Ruminococcaceae |
| OTU_234 | 9 | 30 | 11 | 67 | 81 | 46 | 90 | 71 | 73 | 41 | 162 | 71 | k:Bacteria,p:Firmicutes,c:Clostridia,o:Clostridiales,f:Ruminococcaceae,g:Oscillibacter,s:Oscillibacter_valericigenes |
| OTU_235 | 2 | 5 | 4 | 3 | 5 | 23 | 19 | 22 | 5 | 15 | 5 | 25 | k:Bacteria,p:Firmicutes,c:Erysipelotrichia,o:Erysipelotrichales,f:Erysipelotrichaceae,g:Clostridium_XVIII |
| OTU_236 | 50 | 82 | 17 | 95 | 35 | 34 | 67 | 41 | 29 | 15 | 38 | 130 | k:Bacteria,p:Firmicutes,c:Clostridia,o:Clostridiales,f:Ruminococcaceae |
| OTU_237 | 5 | 5 | 0 | 38 | 3 | 17 | 18 | 39 | 3 | 14 | 0 | 2 | k:Bacteria,p:Firmicutes,c:Clostridia,o:Clostridiales,f:Ruminococcaceae |
| OTU_238 | 54 | 61 | 24 | 7 | 2 | 0 | 5 | 34 | 1 | 0 | 6 | 3 | k:Bacteria,p:Firmicutes,c:Clostridia,o:Clostridiales,f:Lachnospiraceae |
| OTU_239 | 18 | 2 | 1 | 12 | 15 | 6 | 14 | 5 | 26 | 19 | 23 | 14 | k:Bacteria,p:Bacteroidetes,c:Bacteroidia,o:Bacteroidales,f:Porphyromonadaceae |
| OTU_240 | 35 | 18 | 1 | 4 | 4 | 5 | 13 | 12 | 1 | 19 | 10 | 56 | k:Bacteria,p:Firmicutes,c:Clostridia,o:Clostridiales,f:Lachnospiraceae |
| OTU_241 | 102 | 9 | 1 | 17 | 6 | 9 | 0 | 0 | 0 | 0 | 0 | 1 | k:Bacteria |
| OTU_242 | 0 | 3 | 1 | 8 | 3 | 14 | 33 | 10 | 12 | 30 | 26 | 38 | k:Bacteria,p:Firmicutes,c:Clostridia,o:Clostridiales |
| OTU_243 | 24 | 16 | 31 | 29 | 32 | 41 | 69 | 17 | 35 | 85 | 32 | 81 | k:Bacteria,p:Firmicutes,c:Clostridia,o:Clostridiales,f:Clostridiales_Incertae_Sedis_XIII |
| OTU_244 | 2 | 1 | 1 | 16 | 14 | 2 | 3 | 19 | 12 | 20 | 4 | 52 | k:Bacteria,p:Firmicutes,c:Clostridia,o:Clostridiales,f:Ruminococcaceae |
| OTU_245 | 20 | 22 | 118 | 0 | 72 | 73 | 4 | 0 | 1 | 0 | 18 | 0 | k:Bacteria,p:Firmicutes,c:Clostridia,o:Clostridiales |
| OTU_246 | 64 | 15 | 8 | 24 | 1 | 0 | 0 | 1 | 0 | 0 | 0 | 9 | k:Bacteria,p:Firmicutes,c:Clostridia,o:Clostridiales,f:Lachnospiraceae |
| OTU_247 | 2 | 3 | 0 | 0 | 41 | 1 | 28 | 19 | 15 | 5 | 34 | 4 | k:Bacteria,p:Bacteroidetes,c:Bacteroidia,o:Bacteroidales,f:Porphyromonadaceae |
| OTU_248 | 21 | 4 | 24 | 19 | 22 | 29 | 19 | 10 | 10 | 20 | 12 | 23 | k:Bacteria,p:Firmicutes |
| OTU_249 | 3 | 0 | 0 | 1 | 1 | 3 | 16 | 27 | 21 | 2 | 18 | 32 | k:Bacteria,p:Firmicutes,c:Clostridia,o:Clostridiales,f:Ruminococcaceae |
| OTU_250 | 81 | 82 | 22 | 88 | 36 | 47 | 38 | 123 | 16 | 44 | 41 | 76 | k:Bacteria |
| OTU_251 | 46 | 43 | 61 | 2 | 5 | 2 | 13 | 109 | 9 | 0 | 4 | 0 | k:Bacteria,p:Bacteroidetes,c:Bacteroidia,o:Bacteroidales,f:Porphyromonadaceae |
| OTU_252 | 22 | 86 | 20 | 7 | 15 | 3 | 5 | 0 | 0 | 1 | 4 | 0 | k:Bacteria,p:Firmicutes,c:Clostridia,o:Clostridiales,f:Lachnospiraceae |
| OTU_253 | 24 | 1 | 3 | 5 | 53 | 2 | 13 | 8 | 28 | 0 | 25 | 8 | k:Bacteria,p:Bacteroidetes,c:Bacteroidia,o:Bacteroidales,f:Porphyromonadaceae |
| OTU_254 | 7 | 6 | 106 | 1 | 20 | 5 | 11 | 6 | 3 | 1 | 0 | 1 | k:Bacteria |
| OTU_255 | 16 | 17 | 23 | 15 | 5 | 31 | 34 | 35 | 16 | 26 | 25 | 120 | k:Bacteria,p:Firmicutes,c:Clostridia,o:Clostridiales,f:Ruminococcaceae |
| OTU_256 | 19 | 56 | 3 | 10 | 35 | 7 | 20 | 0 | 1 | 5 | 16 | 11 | k:Bacteria,p:Firmicutes,c:Clostridia,o:Clostridiales,f:Ruminococcaceae |
| OTU_257 | 2 | 0 | 3 | 0 | 0 | 14 | 0 | 0 | 0 | 0 | 0 | 104 | k:Bacteria |
| OTU_258 | 21 | 49 | 19 | 16 | 9 | 20 | 16 | 40 | 12 | 37 | 8 | 27 | k:Bacteria,p:Firmicutes,c:Clostridia,o:Clostridiales,f:Ruminococcaceae |
| OTU_259 | 33 | 37 | 104 | 54 | 39 | 23 | 21 | 61 | 15 | 49 | 16 | 49 | k:Bacteria,p:Firmicutes,c:Clostridia,o:Clostridiales |
| OTU_260 | 26 | 19 | 43 | 23 | 4 | 17 | 14 | 20 | 12 | 26 | 13 | 42 | k:Bacteria,p:Firmicutes,c:Clostridia,o:Clostridiales |
| OTU_261 | 6 | 1 | 0 | 37 | 8 | 2 | 7 | 10 | 3 | 3 | 8 | 59 | k:Bacteria,p:Firmicutes,c:Clostridia,o:Clostridiales,f:Ruminococcaceae |
| OTU_262 | 6 | 3 | 9 | 34 | 3 | 25 | 4 | 10 | 1 | 4 | 2 | 3 | k:Bacteria |
| OTU_263 | 4 | 30 | 1 | 9 | 23 | 2 | 4 | 1 | 63 | 0 | 5 | 0 | k:Bacteria,p:Firmicutes,c:Clostridia,o:Clostridiales,f:Lachnospiraceae |
| OTU_264 | 0 | 21 | 1 | 2 | 0 | 25 | 2 | 99 | 3 | 3 | 5 | 0 | k:Bacteria |
| OTU_265 | 0 | 0 | 71 | 3 | 50 | 5 | 0 | 0 | 0 | 1 | 0 | 0 | k:Bacteria,p:Proteobacteria,c:Gammaproteobacteria,o:Pseudomonadales,f:Pseudomonadaceae,g:Pseudomonas,s:Pseudomonas_psychrophila |
| OTU_266 | 0 | 0 | 0 | 21 | 0 | 1 | 22 | 23 | 11 | 28 | 5 | 21 | k:Bacteria,p:Proteobacteria,c:Deltaproteobacteria,o:Desulfovibrionales,f:Desulfovibrionaceae,g:Desulfovibrio |
| OTU_267 | 8 | 3 | 1 | 21 | 3 | 12 | 19 | 25 | 15 | 12 | 41 | 23 | k:Bacteria,p:Firmicutes,c:Clostridia,o:Clostridiales,f:Ruminococcaceae |
| OTU_268 | 3 | 5 | 1 | 15 | 2 | 37 | 21 | 16 | 14 | 19 | 5 | 9 | k:Bacteria,p:Actinobacteria,c:Actinobacteria,o:Coriobacteriales,f:Coriobacteriaceae |
| OTU_269 | 5 | 4 | 0 | 4 | 1 | 14 | 9 | 7 | 21 | 19 | 4 | 24 | k:Bacteria,p:Firmicutes,c:Clostridia,o:Clostridiales |
| OTU_270 | 0 | 0 | 0 | 0 | 0 | 0 | 24 | 0 | 0 | 0 | 0 | 59 | k:Bacteria |
| OTU_271 | 19 | 21 | 17 | 15 | 11 | 7 | 15 | 29 | 11 | 18 | 8 | 13 | k:Bacteria,p:Firmicutes,c:Clostridia,o:Clostridiales,f:Lachnospiraceae,g:Clostridium_XlVa |
| OTU_272 | 0 | 2 | 1 | 9 | 7 | 15 | 10 | 10 | 5 | 9 | 7 | 18 | k:Bacteria,p:Actinobacteria,c:Actinobacteria,o:Coriobacteriales,f:Coriobacteriaceae |
| OTU_273 | 4 | 8 | 2 | 9 | 1 | 3 | 3 | 0 | 31 | 67 | 1 | 14 | k:Bacteria,p:Firmicutes,c:Clostridia,o:Clostridiales,f:Lachnospiraceae |
| OTU_274 | 64 | 21 | 6 | 6 | 15 | 4 | 3 | 5 | 1 | 4 | 52 | 22 | k:Bacteria,p:Firmicutes,c:Clostridia,o:Clostridiales,f:Ruminococcaceae |
| OTU_275 | 1 | 7 | 3 | 0 | 0 | 14 | 11 | 10 | 0 | 0 | 20 | 61 | k:Bacteria |
| OTU_276 | 31 | 19 | 3 | 5 | 6 | 1 | 1 | 5 | 3 | 15 | 7 | 34 | k:Bacteria,p:Bacteroidetes,c:Bacteroidia,o:Bacteroidales,f:Porphyromonadaceae |
| OTU_277 | 24 | 3 | 3 | 4 | 0 | 1 | 2 | 9 | 2 | 58 | 51 | 3 | k:Bacteria,p:Firmicutes,c:Clostridia,o:Clostridiales,f:Lachnospiraceae |
| OTU_278 | 143 | 19 | 83 | 84 | 28 | 14 | 2 | 3 | 2 | 3 | 32 | 20 | k:Bacteria,p:Firmicutes,c:Clostridia,o:Clostridiales,f:Lachnospiraceae |
| OTU_279 | 0 | 0 | 3 | 0 | 4 | 18 | 8 | 16 | 1 | 7 | 21 | 14 | k:Bacteria |
| OTU_280 | 0 | 0 | 48 | 0 | 32 | 7 | 0 | 1 | 0 | 0 | 0 | 0 | k:Bacteria,p:Firmicutes,c:Bacilli,o:Bacillales,f:Bacillaceae_1,g:Geobacillus,s:Geobacillus_stearothermophilus |
| OTU_281 | 2 | 1 | 1 | 0 | 2 | 8 | 4 | 23 | 14 | 50 | 7 | 7 | k:Bacteria,p:Firmicutes,c:Clostridia,o:Clostridiales |
| OTU_282 | 0 | 1 | 0 | 8 | 12 | 3 | 27 | 20 | 21 | 6 | 89 | 12 | k:Bacteria,p:Firmicutes,c:Clostridia,o:Clostridiales,f:Ruminococcaceae,g:Oscillibacter |
| OTU_283 | 9 | 5 | 11 | 21 | 29 | 7 | 254 | 262 | 10 | 59 | 7 | 43 | k:Bacteria,p:Firmicutes,c:Clostridia,o:Clostridiales,f:Lachnospiraceae |
| OTU_284 | 11 | 1 | 1 | 5 | 0 | 6 | 0 | 0 | 4 | 1 | 0 | 49 | k:Bacteria,p:Proteobacteria,c:Epsilonproteobacteria,o:Campylobacterales,f:Helicobacteraceae,g:Helicobacter,s:Helicobacter_typhlonius |
| OTU_285 | 6 | 6 | 3 | 23 | 20 | 20 | 4 | 7 | 20 | 3 | 32 | 14 | k:Bacteria,p:Firmicutes,c:Clostridia,o:Clostridiales,f:Ruminococcaceae,g:Flavonifractor,s:Flavonifractor_plautii |
| OTU_286 | 6 | 2 | 11 | 7 | 1 | 1 | 20 | 38 | 10 | 0 | 8 | 0 | k:Bacteria,p:Firmicutes,c:Clostridia,o:Clostridiales |
| OTU_287 | 1 | 0 | 1 | 5 | 1 | 18 | 12 | 31 | 3 | 11 | 7 | 8 | k:Bacteria,p:Firmicutes,c:Clostridia,o:Clostridiales |
| OTU_288 | 71 | 25 | 27 | 4 | 0 | 1 | 16 | 4 | 15 | 8 | 10 | 1 | k:Bacteria,p:Firmicutes,c:Clostridia,o:Clostridiales,f:Ruminococcaceae |
| OTU_289 | 22 | 40 | 8 | 13 | 7 | 26 | 2 | 1 | 17 | 2 | 11 | 0 | k:Bacteria,p:Firmicutes,c:Clostridia,o:Clostridiales |
| OTU_290 | 6 | 1 | 0 | 16 | 48 | 5 | 0 | 8 | 0 | 0 | 0 | 0 | k:Bacteria,p:Bacteroidetes,c:Bacteroidia,o:Bacteroidales,f:Porphyromonadaceae |
| OTU_291 | 3 | 1 | 0 | 3 | 1 | 6 | 4 | 7 | 5 | 11 | 39 | 7 | k:Bacteria,p:Firmicutes,c:Clostridia,o:Clostridiales,f:Ruminococcaceae |
| OTU_292 | 3 | 6 | 6 | 9 | 1 | 11 | 12 | 10 | 3 | 4 | 5 | 6 | k:Bacteria,p:Actinobacteria,c:Actinobacteria,o:Coriobacteriales,f:Coriobacteriaceae |
| OTU_293 | 34 | 37 | 16 | 135 | 22 | 26 | 23 | 37 | 26 | 9 | 11 | 4 | k:Bacteria |
| OTU_294 | 4 | 2 | 2 | 17 | 7 | 8 | 31 | 11 | 13 | 20 | 13 | 31 | k:Bacteria,p:Firmicutes,c:Clostridia,o:Clostridiales,f:Lachnospiraceae |
| OTU_295 | 1 | 2 | 7 | 8 | 9 | 4 | 26 | 19 | 8 | 5 | 24 | 6 | k:Bacteria,p:Firmicutes,c:Clostridia,o:Clostridiales,f:Ruminococcaceae |
| OTU_296 | 120 | 100 | 35 | 164 | 92 | 158 | 104 | 173 | 323 | 220 | 152 | 229 | k:Bacteria,p:Firmicutes,c:Clostridia,o:Clostridiales,f:Ruminococcaceae |
| OTU_297 | 41 | 30 | 14 | 9 | 30 | 54 | 39 | 7 | 4 | 9 | 20 | 433 | k:Bacteria |
| OTU_298 | 8 | 12 | 22 | 9 | 7 | 12 | 14 | 28 | 19 | 17 | 13 | 15 | k:Bacteria,p:Firmicutes,c:Clostridia,o:Clostridiales,f:Ruminococcaceae |
| OTU_299 | 0 | 0 | 0 | 4 | 0 | 3 | 0 | 0 | 0 | 71 | 2 | 3 | k:Bacteria,p:Firmicutes,c:Clostridia,o:Clostridiales,f:Lachnospiraceae |
| OTU_300 | 3 | 1 | 0 | 5 | 4 | 6 | 24 | 15 | 6 | 13 | 22 | 20 | k:Bacteria,p:Firmicutes,c:Clostridia,o:Clostridiales |
| OTU_301 | 31 | 42 | 95 | 26 | 79 | 132 | 28 | 70 | 36 | 13 | 23 | 18 | k:Bacteria,p:Firmicutes |
| OTU_302 | 0 | 0 | 1 | 1 | 1 | 5 | 8 | 11 | 21 | 0 | 33 | 2 | k:Bacteria,p:Firmicutes,c:Clostridia,o:Clostridiales |
| OTU_303 | 2 | 45 | 6 | 47 | 2 | 3 | 0 | 0 | 0 | 0 | 0 | 0 | k:Bacteria,p:Firmicutes,c:Clostridia,o:Clostridiales,f:Lachnospiraceae |
| OTU_304 | 10 | 5 | 0 | 0 | 1 | 0 | 6 | 6 | 23 | 27 | 16 | 1 | k:Bacteria,p:Bacteroidetes,c:Bacteroidia,o:Bacteroidales,f:Porphyromonadaceae |
| OTU_305 | 44 | 16 | 12 | 77 | 20 | 35 | 32 | 61 | 20 | 33 | 23 | 87 | k:Bacteria,p:Firmicutes,c:Clostridia,o:Clostridiales,f:Lachnospiraceae |
| OTU_306 | 10 | 3 | 4 | 30 | 46 | 1 | 21 | 10 | 31 | 0 | 12 | 5 | k:Bacteria,p:Bacteroidetes,c:Bacteroidia,o:Bacteroidales,f:Porphyromonadaceae |
| OTU_307 | 53 | 61 | 7 | 1 | 8 | 1 | 1 | 0 | 0 | 0 | 0 | 0 | k:Bacteria,p:Bacteroidetes,c:Bacteroidia,o:Bacteroidales,f:Prevotellaceae,g:Prevotella |
| OTU_308 | 61 | 32 | 41 | 77 | 106 | 11 | 18 | 90 | 57 | 30 | 15 | 20 | k:Bacteria,p:Firmicutes,c:Clostridia,o:Clostridiales,f:Lachnospiraceae |
| OTU_309 | 1 | 0 | 0 | 1 | 2 | 2 | 0 | 3 | 13 | 9 | 36 | 29 | k:Bacteria,p:Firmicutes,c:Clostridia,o:Clostridiales,f:Lachnospiraceae,g:Blautia |
| OTU_310 | 4 | 65 | 13 | 0 | 0 | 0 | 0 | 0 | 0 | 0 | 0 | 0 | k:Bacteria,p:Firmicutes,c:Clostridia,o:Clostridiales |
| OTU_311 | 3 | 55 | 1 | 14 | 7 | 3 | 0 | 0 | 0 | 0 | 0 | 0 | k:Bacteria,p:Firmicutes,c:Clostridia,o:Clostridiales |
| OTU_312 | 5 | 1 | 3 | 11 | 21 | 2 | 21 | 70 | 11 | 58 | 8 | 28 | k:Bacteria,p:Bacteroidetes,c:Bacteroidia,o:Bacteroidales,f:Porphyromonadaceae |
| OTU_313 | 1 | 3 | 5 | 17 | 9 | 7 | 6 | 10 | 13 | 6 | 28 | 46 | k:Bacteria,p:Firmicutes,c:Clostridia,o:Clostridiales,f:Ruminococcaceae |
| OTU_314 | 1 | 0 | 2 | 0 | 1 | 0 | 33 | 100 | 9 | 1 | 12 | 10 | k:Bacteria,p:Bacteroidetes,c:Bacteroidia,o:Bacteroidales,f:Porphyromonadaceae |
| OTU_315 | 2 | 4 | 0 | 15 | 2 | 4 | 7 | 9 | 7 | 1 | 7 | 17 | k:Bacteria,p:Firmicutes,c:Clostridia,o:Clostridiales,f:Ruminococcaceae |
| OTU_316 | 23 | 2 | 0 | 0 | 25 | 0 | 4 | 0 | 1 | 0 | 26 | 8 | k:Bacteria,p:Bacteroidetes,c:Bacteroidia,o:Bacteroidales,f:Prevotellaceae,g:Prevotella |
| OTU_317 | 0 | 1 | 114 | 1 | 60 | 13 | 0 | 0 | 1 | 0 | 0 | 1 | k:Bacteria,p:Firmicutes,c:Bacilli,o:Lactobacillales,f:Streptococcaceae,g:Streptococcus,s:Streptococcus_salivarius_subsp._salivarius |
| OTU_318 | 0 | 2 | 17 | 9 | 2 | 0 | 0 | 0 | 4 | 0 | 0 | 53 | k:Bacteria,p:Firmicutes,c:Clostridia,o:Clostridiales,f:Lachnospiraceae,g:Clostridium_XlVa |
| OTU_319 | 25 | 5 | 4 | 0 | 33 | 1 | 5 | 7 | 3 | 0 | 6 | 2 | k:Bacteria,p:Bacteroidetes,c:Bacteroidia,o:Bacteroidales,f:Rikenellaceae,g:Alistipes |
| OTU_320 | 1 | 1 | 5 | 13 | 0 | 3 | 43 | 10 | 15 | 7 | 29 | 6 | k:Bacteria,p:Firmicutes,c:Clostridia,o:Clostridiales,f:Lachnospiraceae |
| OTU_321 | 4 | 2 | 4 | 16 | 7 | 16 | 17 | 11 | 14 | 12 | 3 | 18 | k:Bacteria,p:Firmicutes,c:Clostridia,o:Clostridiales,f:Eubacteriaceae,g:Eubacterium,s:Eubacterium_desmolans |
| OTU_322 | 28 | 5 | 29 | 1 | 6 | 10 | 2 | 2 | 7 | 18 | 4 | 0 | k:Bacteria,p:Firmicutes,c:Clostridia,o:Clostridiales,f:Lachnospiraceae |
| OTU_323 | 1 | 1 | 21 | 4 | 0 | 0 | 1 | 3 | 13 | 14 | 7 | 218 | k:Bacteria,p:Firmicutes,c:Clostridia,o:Clostridiales,f:Lachnospiraceae |
| OTU_324 | 4 | 4 | 1 | 45 | 12 | 8 | 30 | 14 | 6 | 19 | 13 | 16 | k:Bacteria,p:Firmicutes,c:Clostridia,o:Clostridiales,f:Lachnospiraceae |
| OTU_325 | 3 | 11 | 1 | 0 | 0 | 10 | 0 | 0 | 0 | 1 | 51 | 0 | k:Bacteria,p:Firmicutes,c:Clostridia,o:Clostridiales |
| OTU_326 | 4 | 4 | 18 | 29 | 10 | 2 | 42 | 11 | 29 | 15 | 11 | 10 | k:Bacteria,p:Firmicutes,c:Clostridia,o:Clostridiales,f:Lachnospiraceae |
| OTU_327 | 8 | 18 | 10 | 3 | 9 | 8 | 31 | 12 | 0 | 2 | 12 | 19 | k:Bacteria,p:Firmicutes,c:Clostridia,o:Clostridiales |
| OTU_328 | 2 | 1 | 21 | 6 | 0 | 0 | 0 | 0 | 0 | 20 | 10 | 12 | k:Bacteria |
| OTU_329 | 3 | 7 | 4 | 6 | 5 | 19 | 20 | 21 | 12 | 25 | 18 | 55 | k:Bacteria,p:Firmicutes |
| OTU_330 | 1 | 5 | 1 | 48 | 4 | 7 | 8 | 1 | 2 | 1 | 14 | 1 | k:Bacteria,p:Firmicutes,c:Clostridia,o:Clostridiales,f:Lachnospiraceae |
| OTU_331 | 2 | 1 | 5 | 1 | 1 | 5 | 2 | 3 | 8 | 2 | 32 | 10 | k:Bacteria,p:Firmicutes |
| OTU_332 | 1 | 1 | 0 | 17 | 2 | 21 | 7 | 2 | 7 | 1 | 2 | 3 | k:Bacteria,p:Actinobacteria,c:Actinobacteria,o:Coriobacteriales,f:Coriobacteriaceae |
| OTU_333 | 11 | 0 | 1 | 37 | 19 | 20 | 0 | 0 | 0 | 0 | 0 | 0 | k:Bacteria,p:Firmicutes,c:Clostridia,o:Clostridiales,f:Ruminococcaceae,g:Clostridium_IV,s:Eubacterium_siraeum |
| OTU_334 | 5 | 9 | 9 | 7 | 2 | 4 | 7 | 14 | 2 | 9 | 3 | 7 | k:Bacteria,p:Firmicutes,c:Clostridia,o:Clostridiales,f:Ruminococcaceae |
| OTU_335 | 1 | 2 | 0 | 6 | 2 | 8 | 18 | 7 | 2 | 6 | 4 | 10 | k:Bacteria,p:Firmicutes,c:Clostridia,o:Clostridiales |
| OTU_336 | 1 | 34 | 6 | 2 | 1 | 1 | 5 | 6 | 4 | 0 | 7 | 3 | k:Bacteria,p:Firmicutes,c:Clostridia,o:Clostridiales,f:Lachnospiraceae |
| OTU_337 | 0 | 0 | 0 | 0 | 0 | 4 | 0 | 0 | 49 | 0 | 10 | 0 | k:Bacteria,p:Firmicutes,c:Clostridia,o:Clostridiales |
| OTU_338 | 47 | 11 | 1 | 0 | 1 | 0 | 0 | 0 | 0 | 0 | 0 | 0 | k:Bacteria,p:Bacteroidetes,c:Bacteroidia,o:Bacteroidales |
| OTU_339 | 3 | 15 | 9 | 3 | 0 | 5 | 5 | 11 | 19 | 6 | 3 | 15 | k:Bacteria,p:Firmicutes,c:Clostridia,o:Clostridiales |
| OTU_340 | 0 | 0 | 2 | 0 | 0 | 0 | 8 | 37 | 3 | 7 | 6 | 5 | k:Bacteria,p:Bacteroidetes,c:Bacteroidia,o:Bacteroidales,f:Rikenellaceae,g:Alistipes,s:Alistipes_indistinctus |
| OTU_341 | 5 | 7 | 4 | 4 | 13 | 2 | 8 | 20 | 8 | 12 | 13 | 10 | k:Bacteria,p:Bacteroidetes,c:Bacteroidia,o:Bacteroidales,f:Porphyromonadaceae |
| OTU_342 | 7 | 26 | 15 | 4 | 20 | 2 | 8 | 5 | 12 | 16 | 25 | 35 | k:Bacteria |
| OTU_343 | 1 | 0 | 1 | 16 | 0 | 0 | 0 | 0 | 0 | 37 | 0 | 0 | k:Bacteria,p:Firmicutes,c:Clostridia,o:Clostridiales,f:Ruminococcaceae |
| OTU_344 | 4 | 0 | 2 | 0 | 46 | 1 | 1 | 5 | 0 | 3 | 4 | 0 | k:Bacteria,p:Bacteroidetes,c:Bacteroidia,o:Bacteroidales,f:Porphyromonadaceae,g:Butyricimonas,s:Butyricimonas_synergistica |
| OTU_345 | 0 | 0 | 1 | 2 | 14 | 0 | 0 | 0 | 5 | 1 | 21 | 7 | k:Bacteria,p:Firmicutes,c:Clostridia,o:Clostridiales,f:Lachnospiraceae |
| OTU_346 | 3 | 0 | 1 | 2 | 2 | 50 | 0 | 0 | 0 | 1 | 0 | 0 | k:Bacteria,p:Firmicutes,c:Clostridia,o:Clostridiales,f:Ruminococcaceae |
| OTU_347 | 1 | 19 | 11 | 1 | 15 | 3 | 8 | 99 | 4 | 1 | 2 | 2 | k:Bacteria,p:Bacteroidetes,c:Bacteroidia,o:Bacteroidales,f:Porphyromonadaceae |
| OTU_348 | 2 | 2 | 4 | 3 | 2 | 8 | 6 | 21 | 5 | 19 | 5 | 5 | k:Bacteria,p:Firmicutes,c:Clostridia,o:Clostridiales,f:Ruminococcaceae |
| OTU_349 | 49 | 81 | 3 | 24 | 25 | 57 | 2 | 11 | 2 | 0 | 4 | 11 | k:Bacteria |
| OTU_350 | 2 | 5 | 6 | 3 | 3 | 1 | 7 | 14 | 2 | 2 | 4 | 10 | k:Bacteria,p:Firmicutes,c:Clostridia,o:Clostridiales,f:Lachnospiraceae |
| OTU_351 | 12 | 13 | 14 | 10 | 13 | 16 | 65 | 23 | 152 | 151 | 399 | 662 | k:Bacteria,p:Firmicutes,c:Clostridia,o:Clostridiales,f:Ruminococcaceae |
| OTU_352 | 4 | 1 | 1 | 10 | 10 | 16 | 2 | 0 | 0 | 1 | 3 | 2 | k:Bacteria,p:Firmicutes,c:Clostridia,o:Clostridiales,f:Ruminococcaceae |
| OTU_353 | 0 | 52 | 0 | 0 | 0 | 4 | 0 | 0 | 0 | 10 | 0 | 0 | k:Bacteria,p:Firmicutes,c:Clostridia,o:Clostridiales |
| OTU_354 | 7 | 1 | 0 | 0 | 1 | 10 | 3 | 5 | 3 | 1 | 11 | 4 | k:Bacteria |
| OTU_355 | 5 | 5 | 1 | 11 | 2 | 0 | 6 | 7 | 5 | 14 | 19 | 13 | k:Bacteria,p:Firmicutes,c:Clostridia,o:Clostridiales,f:Ruminococcaceae |
| OTU_356 | 0 | 0 | 0 | 0 | 0 | 0 | 0 | 51 | 0 | 0 | 0 | 0 | k:Bacteria,p:Tenericutes,c:Mollicutes |
| OTU_357 | 3 | 4 | 10 | 12 | 7 | 18 | 11 | 14 | 5 | 15 | 5 | 8 | k:Bacteria,p:Firmicutes,c:Clostridia,o:Clostridiales,f:Ruminococcaceae,g:Clostridium_III |
| OTU_358 | 3 | 3 | 0 | 57 | 0 | 57 | 0 | 0 | 0 | 0 | 1 | 0 | k:Bacteria |
| OTU_359 | 3 | 2 | 0 | 0 | 0 | 3 | 1 | 0 | 6 | 16 | 6 | 17 | k:Bacteria,p:Firmicutes,c:Clostridia,o:Clostridiales,f:Lachnospiraceae |
| OTU_360 | 19 | 27 | 20 | 10 | 2 | 26 | 0 | 2 | 3 | 3 | 26 | 7 | k:Bacteria |
| OTU_361 | 84 | 22 | 12 | 17 | 12 | 50 | 17 | 24 | 9 | 29 | 4 | 115 | k:Bacteria,p:Firmicutes,c:Clostridia,o:Clostridiales |
| OTU_362 | 56 | 0 | 2 | 0 | 0 | 0 | 0 | 0 | 0 | 0 | 0 | 0 | k:Bacteria |
| OTU_363 | 3 | 29 | 11 | 27 | 0 | 4 | 2 | 0 | 0 | 2 | 0 | 0 | k:Bacteria,p:Firmicutes,c:Clostridia,o:Clostridiales,f:Lachnospiraceae |
| OTU_364 | 0 | 0 | 0 | 8 | 5 | 4 | 27 | 19 | 1 | 8 | 5 | 0 | k:Bacteria,p:Firmicutes,c:Clostridia,o:Clostridiales,f:Ruminococcaceae |
| OTU_365 | 38 | 19 | 0 | 5 | 15 | 3 | 4 | 3 | 5 | 9 | 6 | 2 | k:Bacteria,p:Bacteroidetes,c:Bacteroidia,o:Bacteroidales |
| OTU_366 | 0 | 1 | 0 | 1 | 62 | 1 | 2 | 2 | 0 | 0 | 1 | 1 | k:Bacteria,p:Bacteroidetes,c:Bacteroidia,o:Bacteroidales,f:Porphyromonadaceae,g:Butyricimonas,s:Butyricimonas_virosa |
| OTU_367 | 12 | 16 | 7 | 5 | 5 | 10 | 4 | 1 | 3 | 0 | 2 | 3 | k:Bacteria,p:Firmicutes,c:Erysipelotrichia,o:Erysipelotrichales,f:Erysipelotrichaceae,g:Turicibacter,s:Turicibacter_sanguinis |
| OTU_368 | 6 | 37 | 8 | 4 | 0 | 3 | 22 | 23 | 26 | 11 | 0 | 7 | k:Bacteria,p:Firmicutes,c:Clostridia,o:Clostridiales,f:Lachnospiraceae |
| OTU_369 | 1 | 21 | 23 | 0 | 0 | 0 | 0 | 1 | 0 | 0 | 0 | 3 | k:Bacteria,p:Bacteroidetes,c:Bacteroidia,o:Bacteroidales,f:Prevotellaceae |
| OTU_370 | 3 | 1 | 0 | 2 | 4 | 10 | 4 | 6 | 10 | 1 | 4 | 7 | k:Bacteria,p:Bacteroidetes,c:Bacteroidia,o:Bacteroidales |
| OTU_371 | 44 | 2 | 0 | 1 | 3 | 8 | 0 | 0 | 0 | 0 | 0 | 0 | k:Bacteria,p:Firmicutes,c:Clostridia,o:Clostridiales |
| OTU_372 | 16 | 16 | 7 | 1 | 2 | 8 | 0 | 25 | 5 | 5 | 0 | 0 | k:Bacteria,p:Firmicutes,c:Clostridia |
| OTU_373 | 51 | 0 | 0 | 0 | 1 | 0 | 0 | 0 | 0 | 0 | 0 | 0 | k:Bacteria,p:Firmicutes,c:Clostridia,o:Clostridiales,f:Lachnospiraceae |
| OTU_374 | 202 | 71 | 108 | 129 | 108 | 162 | 131 | 176 | 114 | 139 | 107 | 186 | k:Bacteria,p:Firmicutes,c:Clostridia,o:Clostridiales,f:Ruminococcaceae |
| OTU_375 | 0 | 0 | 38 | 1 | 18 | 4 | 1 | 0 | 0 | 0 | 0 | 0 | k:Bacteria,p:Proteobacteria,c:Gammaproteobacteria,o:Pseudomonadales,f:Pseudomonadaceae,g:Pseudomonas |
| OTU_376 | 0 | 1 | 1 | 4 | 0 | 4 | 0 | 3 | 0 | 0 | 4 | 30 | k:Bacteria,p:Firmicutes,c:Clostridia,o:Clostridiales,f:Lachnospiraceae |
| OTU_377 | 8 | 8 | 11 | 11 | 9 | 8 | 24 | 5 | 5 | 10 | 5 | 16 | k:Bacteria,p:Firmicutes,c:Clostridia,o:Clostridiales,f:Lachnospiraceae |
| OTU_378 | 4 | 0 | 0 | 13 | 25 | 19 | 1 | 0 | 11 | 0 | 3 | 1 | k:Bacteria |
| OTU_379 | 0 | 3 | 0 | 0 | 1 | 0 | 0 | 57 | 0 | 1 | 0 | 3 | k:Bacteria,p:Firmicutes,c:Clostridia,o:Clostridiales,f:Lachnospiraceae |
| OTU_380 | 2 | 1 | 2 | 0 | 0 | 0 | 1 | 3 | 2 | 7 | 6 | 41 | k:Bacteria,p:Firmicutes,c:Clostridia,o:Clostridiales,f:Lachnospiraceae |
| OTU_381 | 5 | 2 | 2 | 0 | 20 | 0 | 1 | 4 | 1 | 1 | 3 | 1 | k:Bacteria,p:Bacteroidetes,c:Bacteroidia,o:Bacteroidales,f:Rikenellaceae,g:Alistipes |
| OTU_382 | 1 | 0 | 0 | 5 | 2 | 12 | 0 | 20 | 0 | 0 | 0 | 25 | k:Bacteria,p:Firmicutes,c:Clostridia,o:Clostridiales |
| OTU_383 | 7 | 11 | 7 | 3 | 6 | 0 | 11 | 5 | 2 | 1 | 3 | 2 | k:Bacteria,p:Bacteroidetes,c:Bacteroidia,o:Bacteroidales |
| OTU_384 | 15 | 6 | 12 | 4 | 1 | 29 | 2 | 0 | 16 | 6 | 6 | 2 | k:Bacteria |
| OTU_385 | 3 | 0 | 0 | 5 | 5 | 3 | 9 | 4 | 1 | 3 | 11 | 0 | k:Bacteria,p:Firmicutes,c:Clostridia,o:Clostridiales |
| OTU_386 | 192 | 8 | 15 | 0 | 3 | 1 | 10 | 38 | 14 | 0 | 7 | 7 | k:Bacteria,p:Firmicutes,c:Clostridia,o:Clostridiales,f:Lachnospiraceae |
| OTU_387 | 2 | 1 | 13 | 0 | 0 | 3 | 0 | 0 | 15 | 7 | 1 | 1 | k:Bacteria,p:Firmicutes,c:Clostridia,o:Clostridiales |
| OTU_388 | 6 | 3 | 0 | 14 | 3 | 4 | 0 | 0 | 0 | 9 | 0 | 0 | k:Bacteria,p:Firmicutes,c:Clostridia,o:Clostridiales,f:Lachnospiraceae |
| OTU_389 | 1 | 3 | 0 | 3 | 7 | 0 | 3 | 20 | 1 | 4 | 0 | 0 | k:Bacteria,p:Bacteroidetes,c:Bacteroidia,o:Bacteroidales |
| OTU_390 | 5 | 0 | 5 | 0 | 0 | 9 | 4 | 4 | 3 | 3 | 6 | 12 | k:Bacteria,p:Firmicutes,c:Erysipelotrichia,o:Erysipelotrichales,f:Erysipelotrichaceae,g:Holdemania,s:Holdemania_filiformis |
| OTU_391 | 8 | 1 | 0 | 0 | 59 | 1 | 3 | 1 | 1 | 1 | 4 | 3 | k:Bacteria,p:Bacteroidetes,c:Bacteroidia,o:Bacteroidales,f:Porphyromonadaceae,g:Parabacteroides,s:Parabacteroides_distasonis |
| OTU_392 | 3 | 3 | 4 | 22 | 23 | 10 | 8 | 24 | 18 | 8 | 21 | 34 | k:Bacteria,p:Firmicutes,c:Clostridia,o:Clostridiales,f:Ruminococcaceae |
| OTU_393 | 2 | 1 | 1 | 1 | 4 | 2 | 10 | 10 | 5 | 3 | 3 | 6 | k:Bacteria,p:Actinobacteria,c:Actinobacteria,o:Coriobacteriales,f:Coriobacteriaceae |
| OTU_394 | 2 | 6 | 2 | 22 | 9 | 11 | 7 | 14 | 10 | 4 | 7 | 10 | k:Bacteria,p:Firmicutes,c:Clostridia,o:Clostridiales,f:Lachnospiraceae |
| OTU_395 | 5 | 4 | 3 | 0 | 31 | 0 | 0 | 0 | 0 | 0 | 0 | 0 | k:Bacteria,p:Bacteroidetes,c:Bacteroidia,o:Bacteroidales,f:Rikenellaceae,g:Alistipes,s:Alistipes_indistinctus |
| OTU_396 | 1 | 1 | 1 | 3 | 1 | 15 | 10 | 3 | 4 | 6 | 0 | 3 | k:Bacteria,p:Firmicutes,c:Clostridia,o:Clostridiales,f:Lachnospiraceae |
| OTU_397 | 5 | 6 | 2 | 0 | 24 | 0 | 0 | 3 | 0 | 0 | 0 | 0 | k:Bacteria,p:Bacteroidetes |
| OTU_398 | 17 | 2 | 0 | 6 | 1 | 0 | 0 | 0 | 0 | 0 | 2 | 0 | k:Bacteria,p:Firmicutes,c:Clostridia,o:Clostridiales,f:Lachnospiraceae,g:Clostridium_XlVa |
| OTU_399 | 24 | 21 | 20 | 13 | 14 | 29 | 25 | 157 | 24 | 37 | 19 | 22 | k:Bacteria,p:Firmicutes,c:Clostridia,o:Clostridiales,f:Ruminococcaceae |
| OTU_400 | 12 | 17 | 4 | 8 | 16 | 1 | 6 | 4 | 0 | 0 | 2 | 0 | k:Bacteria,p:Firmicutes,c:Clostridia,o:Clostridiales,f:Lachnospiraceae |
| OTU_401 | 1 | 0 | 0 | 0 | 0 | 2 | 3 | 0 | 8 | 8 | 4 | 7 | k:Bacteria,p:Firmicutes,c:Clostridia,o:Clostridiales,f:Lachnospiraceae |
| OTU_402 | 6 | 6 | 12 | 37 | 28 | 21 | 26 | 23 | 17 | 12 | 38 | 27 | k:Bacteria,p:Firmicutes,c:Clostridia,o:Clostridiales,f:Ruminococcaceae |
| OTU_403 | 10 | 5 | 4 | 2 | 1 | 0 | 16 | 7 | 1 | 4 | 9 | 3 | k:Bacteria,p:Firmicutes,c:Clostridia,o:Clostridiales,f:Ruminococcaceae |
| OTU_404 | 5 | 13 | 43 | 3 | 5 | 4 | 0 | 1 | 0 | 0 | 0 | 0 | k:Bacteria,p:Firmicutes,c:Clostridia,o:Clostridiales,f:Lachnospiraceae |
| OTU_405 | 1 | 5 | 1 | 2 | 4 | 0 | 3 | 35 | 4 | 3 | 0 | 0 | k:Bacteria |
| OTU_406 | 8 | 6 | 5 | 2 | 33 | 0 | 7 | 13 | 5 | 4 | 1 | 7 | k:Bacteria,p:Bacteroidetes,c:Bacteroidia,o:Bacteroidales,f:Bacteroidaceae,g:Bacteroides,s:Bacteroides_xylanisolvens |
| OTU_407 | 0 | 9 | 0 | 18 | 0 | 15 | 2 | 8 | 1 | 3 | 0 | 4 | k:Bacteria,p:Actinobacteria,c:Actinobacteria,o:Coriobacteriales,f:Coriobacteriaceae |
| OTU_408 | 1 | 3 | 2 | 0 | 8 | 0 | 3 | 0 | 12 | 0 | 7 | 11 | k:Bacteria,p:Firmicutes |
| OTU_409 | 0 | 0 | 0 | 22 | 3 | 4 | 0 | 0 | 0 | 0 | 0 | 0 | k:Bacteria,p:Firmicutes,c:Clostridia,o:Clostridiales |
| OTU_410 | 5 | 8 | 11 | 2 | 5 | 5 | 4 | 4 | 2 | 2 | 3 | 3 | k:Bacteria,p:Firmicutes,c:Clostridia,o:Clostridiales |
| OTU_411 | 0 | 3 | 35 | 0 | 0 | 0 | 0 | 0 | 0 | 1 | 0 | 1 | k:Bacteria,p:Firmicutes,c:Clostridia,o:Clostridiales,f:Lachnospiraceae |
| OTU_412 | 17 | 2 | 0 | 0 | 12 | 0 | 5 | 9 | 0 | 0 | 5 | 1 | k:Bacteria,p:Bacteroidetes,c:Bacteroidia,o:Bacteroidales,f:Porphyromonadaceae |
| OTU_413 | 2 | 1 | 2 | 4 | 3 | 0 | 13 | 0 | 13 | 0 | 5 | 0 | k:Bacteria,p:Firmicutes,c:Clostridia,o:Clostridiales,f:Ruminococcaceae |
| OTU_414 | 2 | 0 | 0 | 9 | 2 | 1 | 2 | 7 | 1 | 4 | 1 | 18 | k:Bacteria,p:Firmicutes,c:Clostridia,o:Clostridiales,f:Lachnospiraceae |
| OTU_415 | 2 | 2 | 7 | 1 | 6 | 0 | 10 | 3 | 0 | 1 | 2 | 4 | k:Bacteria,p:Firmicutes,c:Clostridia,o:Clostridiales,f:Lachnospiraceae |
| OTU_416 | 6 | 11 | 19 | 11 | 43 | 0 | 12 | 10 | 1 | 8 | 2 | 9 | k:Bacteria,p:Firmicutes,c:Clostridia,o:Clostridiales,f:Lachnospiraceae |
| OTU_417 | 5 | 1 | 9 | 8 | 4 | 5 | 7 | 5 | 2 | 1 | 10 | 15 | k:Bacteria,p:Firmicutes,c:Clostridia,o:Clostridiales,f:Ruminococcaceae,g:Clostridium_III |
| OTU_418 | 2 | 1 | 1 | 0 | 1 | 5 | 0 | 0 | 0 | 22 | 4 | 1 | k:Bacteria,p:Firmicutes,c:Clostridia,o:Clostridiales,f:Ruminococcaceae |
| OTU_419 | 0 | 0 | 7 | 1 | 0 | 22 | 0 | 0 | 0 | 0 | 0 | 0 | k:Bacteria,p:Firmicutes,c:Clostridia,o:Clostridiales,f:Lachnospiraceae |
| OTU_420 | 5 | 2 | 10 | 13 | 15 | 21 | 9 | 3 | 1 | 40 | 3 | 9 | k:Bacteria,p:Candidatus_Saccharibacteria,g:Saccharibacteria_genera_incertae_sedis |
| OTU_421 | 8 | 1 | 0 | 0 | 22 | 2 | 0 | 0 | 0 | 0 | 1 | 0 | k:Bacteria,p:Bacteroidetes,c:Bacteroidia,o:Bacteroidales,f:Prevotellaceae,g:Paraprevotella,s:Paraprevotella_clara |
| OTU_422 | 3 | 5 | 3 | 9 | 3 | 9 | 6 | 3 | 4 | 11 | 5 | 11 | k:Bacteria,p:Firmicutes,c:Clostridia,o:Clostridiales,f:Lachnospiraceae |
| OTU_423 | 5 | 4 | 2 | 15 | 11 | 13 | 49 | 12 | 30 | 13 | 6 | 74 | k:Bacteria,p:Firmicutes,c:Clostridia,o:Clostridiales,f:Lachnospiraceae |
| OTU_424 | 5 | 12 | 10 | 1 | 0 | 7 | 1 | 0 | 1 | 1 | 0 | 3 | k:Bacteria,p:Actinobacteria,c:Actinobacteria,o:Coriobacteriales,f:Coriobacteriaceae |
| OTU_425 | 0 | 0 | 15 | 0 | 16 | 0 | 0 | 0 | 0 | 0 | 0 | 0 | k:Bacteria,p:Proteobacteria,c:Gammaproteobacteria,o:Oceanospirillales,f:Halomonadaceae,g:Halomonas |
| OTU_426 | 2 | 3 | 0 | 6 | 3 | 17 | 0 | 0 | 1 | 5 | 0 | 3 | k:Bacteria,p:Firmicutes,c:Clostridia,o:Clostridiales |
| OTU_427 | 2 | 1 | 0 | 0 | 0 | 2 | 2 | 0 | 18 | 5 | 7 | 9 | k:Bacteria,p:Firmicutes,c:Clostridia,o:Clostridiales,f:Lachnospiraceae |
| OTU_428 | 58 | 35 | 1 | 0 | 4 | 5 | 20 | 0 | 1 | 6 | 0 | 5 | k:Bacteria,p:Firmicutes,c:Clostridia,o:Clostridiales,f:Ruminococcaceae,g:Ruminococcus |
| OTU_429 | 0 | 1 | 0 | 13 | 6 | 18 | 0 | 0 | 0 | 0 | 0 | 0 | k:Bacteria,p:Firmicutes |
| OTU_430 | 3 | 1 | 5 | 0 | 0 | 1 | 0 | 0 | 0 | 0 | 34 | 0 | k:Bacteria,p:Firmicutes,c:Clostridia,o:Clostridiales,f:Lachnospiraceae,g:Clostridium_XlVa |
| OTU_431 | 7 | 5 | 1 | 3 | 2 | 4 | 4 | 0 | 0 | 0 | 1 | 1 | k:Bacteria |
| OTU_432 | 1 | 0 | 0 | 10 | 0 | 6 | 0 | 0 | 0 | 11 | 0 | 9 | k:Bacteria,p:Firmicutes,c:Clostridia,o:Clostridiales |
| OTU_433 | 3 | 0 | 2 | 2 | 0 | 0 | 6 | 5 | 2 | 2 | 11 | 4 | k:Bacteria,p:Firmicutes,c:Clostridia,o:Clostridiales,f:Lachnospiraceae |
| OTU_434 | 72 | 27 | 11 | 22 | 42 | 2 | 1 | 1 | 0 | 1 | 6 | 2 | k:Bacteria,p:Firmicutes,c:Clostridia,o:Clostridiales,f:Ruminococcaceae |
| OTU_435 | 5 | 0 | 0 | 0 | 0 | 0 | 2 | 9 | 3 | 5 | 14 | 3 | k:Bacteria,p:Firmicutes,c:Clostridia,o:Clostridiales,f:Lachnospiraceae |
| OTU_436 | 0 | 2 | 1 | 5 | 2 | 2 | 7 | 4 | 3 | 5 | 6 | 2 | k:Bacteria,p:Firmicutes,c:Clostridia,o:Clostridiales,f:Ruminococcaceae |
| OTU_437 | 9 | 2 | 0 | 1 | 0 | 7 | 2 | 3 | 0 | 0 | 0 | 2 | k:Bacteria |
| OTU_438 | 18 | 5 | 6 | 4 | 3 | 2 | 4 | 2 | 6 | 2 | 2 | 5 | k:Bacteria,p:Firmicutes,c:Clostridia,o:Clostridiales,f:Ruminococcaceae,g:Clostridium_IV |
| OTU_439 | 9 | 1 | 0 | 2 | 1 | 0 | 0 | 2 | 2 | 0 | 2 | 1 | k:Bacteria,p:Bacteroidetes,c:Bacteroidia,o:Bacteroidales,f:Rikenellaceae,g:Alistipes |
| OTU_440 | 0 | 0 | 0 | 7 | 1 | 11 | 1 | 5 | 1 | 2 | 2 | 6 | k:Bacteria,p:Firmicutes,c:Clostridia,o:Clostridiales,f:Christensenellaceae,g:Christensenella,s:Christensenella_minuta |
| OTU_441 | 4 | 1 | 2 | 3 | 12 | 2 | 12 | 11 | 3 | 2 | 5 | 3 | k:Bacteria,p:Firmicutes,c:Clostridia,o:Clostridiales,f:Ruminococcaceae |
| OTU_442 | 17 | 4 | 7 | 4 | 34 | 5 | 5 | 10 | 2 | 2 | 9 | 8 | k:Bacteria,p:Bacteroidetes,c:Bacteroidia,o:Bacteroidales,f:Porphyromonadaceae |
| OTU_443 | 2 | 6 | 0 | 7 | 2 | 6 | 3 | 1 | 0 | 1 | 2 | 7 | k:Bacteria,p:Actinobacteria,c:Actinobacteria,o:Coriobacteriales,f:Coriobacteriaceae |
| OTU_444 | 0 | 0 | 0 | 0 | 0 | 0 | 0 | 0 | 1 | 2 | 13 | 13 | k:Bacteria,p:Firmicutes,c:Clostridia,o:Clostridiales,f:Lachnospiraceae |
| OTU_445 | 0 | 4 | 3 | 9 | 7 | 3 | 11 | 15 | 11 | 8 | 14 | 12 | k:Bacteria,p:Firmicutes,c:Clostridia,o:Clostridiales,f:Ruminococcaceae,g:Oscillibacter,s:Oscillibacter_valericigenes |
| OTU_446 | 0 | 0 | 0 | 3 | 8 | 0 | 6 | 10 | 1 | 1 | 3 | 7 | k:Bacteria,p:Firmicutes,c:Clostridia,o:Clostridiales,f:Lachnospiraceae |
| OTU_447 | 0 | 0 | 0 | 0 | 0 | 2 | 0 | 0 | 2 | 2 | 7 | 11 | k:Bacteria,p:Firmicutes,c:Clostridia,o:Clostridiales,f:Lachnospiraceae |
| OTU_448 | 35 | 3 | 8 | 0 | 13 | 0 | 5 | 9 | 4 | 1 | 26 | 8 | k:Bacteria,p:Bacteroidetes,c:Bacteroidia,o:Bacteroidales,f:Bacteroidaceae,g:Bacteroides |
| OTU_449 | 4 | 3 | 0 | 5 | 1 | 9 | 5 | 2 | 2 | 1 | 0 | 5 | k:Bacteria,p:Firmicutes |
| OTU_450 | 4 | 5 | 5 | 2 | 1 | 6 | 6 | 3 | 2 | 5 | 3 | 8 | k:Bacteria,p:Firmicutes,c:Clostridia,o:Clostridiales,f:Lachnospiraceae,g:Blautia |
| OTU_451 | 0 | 2 | 26 | 0 | 0 | 1 | 1 | 0 | 0 | 1 | 0 | 0 | k:Bacteria,p:Firmicutes,c:Clostridia,o:Clostridiales,f:Lachnospiraceae |
| OTU_452 | 6 | 6 | 4 | 3 | 1 | 2 | 14 | 15 | 0 | 11 | 4 | 4 | k:Bacteria,p:Firmicutes,c:Clostridia,o:Clostridiales,f:Lachnospiraceae |
| OTU_453 | 8 | 23 | 10 | 12 | 3 | 14 | 9 | 4 | 3 | 4 | 7 | 11 | k:Bacteria,p:Firmicutes,c:Clostridia,o:Clostridiales,f:Lachnospiraceae |
| OTU_454 | 0 | 1 | 2 | 0 | 2 | 0 | 0 | 0 | 4 | 2 | 18 | 0 | k:Bacteria,p:Firmicutes,c:Clostridia,o:Clostridiales |
| OTU_455 | 7 | 1 | 0 | 0 | 12 | 1 | 0 | 2 | 1 | 0 | 2 | 0 | k:Bacteria,p:Bacteroidetes |
| OTU_456 | 1 | 0 | 0 | 0 | 0 | 2 | 0 | 1 | 6 | 0 | 7 | 7 | k:Bacteria,p:Firmicutes,c:Clostridia |
| OTU_457 | 7 | 2 | 5 | 4 | 20 | 2 | 7 | 13 | 3 | 7 | 25 | 15 | k:Bacteria,p:Firmicutes,c:Clostridia,o:Clostridiales,f:Ruminococcaceae |
| OTU_458 | 2 | 3 | 0 | 4 | 1 | 9 | 0 | 0 | 1 | 1 | 0 | 1 | k:Bacteria,p:Actinobacteria,c:Actinobacteria,o:Bifidobacteriales,f:Bifidobacteriaceae,g:Bifidobacterium,s:Bifidobacterium_pseudolongum_subsp._globosum |
| OTU_459 | 0 | 0 | 1 | 15 | 0 | 11 | 0 | 0 | 0 | 0 | 0 | 0 | k:Bacteria,p:Firmicutes,c:Clostridia,o:Clostridiales |
| OTU_460 | 23 | 0 | 0 | 0 | 1 | 1 | 0 | 0 | 0 | 0 | 0 | 0 | k:Bacteria,p:Firmicutes,c:Clostridia,o:Clostridiales,f:Ruminococcaceae,g:Ruminococcus |
| OTU_461 | 7 | 28 | 21 | 1 | 8 | 0 | 0 | 5 | 1 | 0 | 1 | 1 | k:Bacteria,p:Bacteroidetes,c:Bacteroidia,o:Bacteroidales |
| OTU_462 | 0 | 0 | 0 | 0 | 0 | 0 | 5 | 0 | 19 | 25 | 0 | 4 | k:Bacteria,p:Firmicutes,c:Clostridia,o:Clostridiales,f:Lachnospiraceae |
| OTU_463 | 2 | 3 | 0 | 0 | 2 | 1 | 19 | 12 | 3 | 3 | 0 | 2 | k:Bacteria,p:Bacteroidetes,c:Bacteroidia,o:Bacteroidales,f:Porphyromonadaceae |
| OTU_464 | 0 | 2 | 1 | 0 | 4 | 1 | 6 | 7 | 1 | 2 | 7 | 1 | k:Bacteria,p:Firmicutes,c:Clostridia,o:Clostridiales,f:Ruminococcaceae |
| OTU_465 | 0 | 0 | 1 | 0 | 2 | 0 | 2 | 15 | 1 | 2 | 26 | 0 | k:Bacteria,p:Firmicutes,c:Clostridia,o:Clostridiales |
| OTU_466 | 0 | 0 | 0 | 0 | 0 | 2 | 10 | 0 | 6 | 6 | 0 | 2 | k:Bacteria,p:Firmicutes,c:Clostridia,o:Clostridiales |
| OTU_467 | 0 | 0 | 0 | 1 | 0 | 3 | 1 | 0 | 0 | 3 | 5 | 13 | k:Bacteria,p:Firmicutes,c:Clostridia,o:Clostridiales |
| OTU_468 | 0 | 3 | 3 | 2 | 1 | 3 | 1 | 5 | 0 | 0 | 3 | 6 | k:Bacteria,p:Firmicutes,c:Erysipelotrichia,o:Erysipelotrichales,f:Erysipelotrichaceae,g:Clostridium_XVIII |
| OTU_469 | 12 | 13 | 12 | 17 | 5 | 39 | 30 | 68 | 33 | 15 | 82 | 243 | k:Bacteria,p:Firmicutes,c:Clostridia,o:Clostridiales,f:Ruminococcaceae |
| OTU_470 | 0 | 5 | 0 | 0 | 0 | 4 | 2 | 0 | 8 | 3 | 5 | 27 | k:Bacteria,p:Firmicutes,c:Clostridia,o:Clostridiales,f:Lachnospiraceae,g:Blautia,s:Blautia_faecis |
| OTU_471 | 4 | 2 | 13 | 0 | 0 | 0 | 0 | 0 | 0 | 0 | 0 | 0 | k:Bacteria,p:Firmicutes,c:Clostridia,o:Clostridiales,f:Ruminococcaceae |
| OTU_472 | 2 | 1 | 0 | 1 | 0 | 1 | 1 | 3 | 3 | 1 | 11 | 6 | k:Bacteria,p:Firmicutes,c:Clostridia,o:Clostridiales |
| OTU_473 | 2 | 1 | 4 | 7 | 0 | 1 | 4 | 1 | 1 | 2 | 3 | 8 | k:Bacteria,p:Firmicutes,c:Clostridia,o:Clostridiales,f:Lachnospiraceae |
| OTU_474 | 14 | 3 | 11 | 0 | 1 | 0 | 1 | 10 | 7 | 3 | 22 | 11 | k:Bacteria,p:Bacteroidetes,c:Bacteroidia,o:Bacteroidales |
| OTU_475 | 5 | 4 | 4 | 12 | 9 | 2 | 0 | 2 | 0 | 0 | 1 | 2 | k:Bacteria,p:Firmicutes,c:Clostridia,o:Clostridiales,f:Lachnospiraceae |
| OTU_476 | 4 | 1 | 0 | 1 | 0 | 3 | 0 | 0 | 7 | 0 | 0 | 4 | k:Bacteria,p:Firmicutes,c:Clostridia,o:Clostridiales |
| OTU_477 | 0 | 2 | 1 | 5 | 3 | 2 | 4 | 15 | 3 | 8 | 4 | 2 | k:Bacteria,p:Firmicutes,c:Clostridia,o:Clostridiales,f:Lachnospiraceae |
| OTU_478 | 1 | 0 | 3 | 8 | 8 | 13 | 9 | 4 | 3 | 9 | 13 | 15 | k:Bacteria,p:Firmicutes,c:Bacilli,o:Lactobacillales,f:Streptococcaceae,g:Streptococcus |
| OTU_479 | 4 | 0 | 0 | 0 | 36 | 0 | 0 | 0 | 0 | 0 | 2 | 0 | k:Bacteria,p:Bacteroidetes,c:Bacteroidia,o:Bacteroidales,f:Porphyromonadaceae,g:Barnesiella,s:Barnesiella_intestinihominis |
| OTU_480 | 3 | 0 | 0 | 2 | 27 | 0 | 0 | 0 | 0 | 0 | 0 | 0 | k:Bacteria,p:Firmicutes,c:Clostridia,o:Clostridiales,f:Lachnospiraceae,g:Acetatifactor,s:Acetatifactor_muris |
| OTU_481 | 0 | 0 | 0 | 0 | 0 | 0 | 0 | 0 | 3 | 0 | 8 | 8 | k:Bacteria,p:Firmicutes,c:Clostridia,o:Clostridiales,f:Ruminococcaceae |
| OTU_482 | 2 | 0 | 1 | 2 | 0 | 3 | 16 | 6 | 3 | 4 | 8 | 2 | k:Bacteria,p:Firmicutes,c:Clostridia,o:Clostridiales,f:Ruminococcaceae |
| OTU_483 | 5 | 0 | 0 | 0 | 20 | 0 | 1 | 0 | 0 | 0 | 0 | 0 | k:Bacteria,p:Bacteroidetes,c:Bacteroidia,o:Bacteroidales,f:Prevotellaceae |
| OTU_484 | 5 | 5 | 47 | 8 | 1 | 22 | 6 | 2 | 9 | 23 | 2 | 53 | k:Bacteria,p:Firmicutes,c:Clostridia,o:Clostridiales |
| OTU_485 | 0 | 1 | 0 | 2 | 4 | 2 | 1 | 3 | 2 | 0 | 6 | 9 | k:Bacteria |
| OTU_486 | 9 | 5 | 4 | 0 | 5 | 2 | 4 | 1 | 3 | 0 | 3 | 1 | k:Bacteria,p:Firmicutes,c:Clostridia,o:Clostridiales,f:Ruminococcaceae |
| OTU_487 | 1 | 6 | 3 | 0 | 5 | 1 | 0 | 0 | 0 | 0 | 14 | 3 | k:Bacteria,p:Firmicutes,c:Clostridia,o:Clostridiales,f:Lachnospiraceae |
| OTU_488 | 2 | 30 | 11 | 6 | 5 | 0 | 0 | 15 | 1 | 0 | 2 | 0 | k:Bacteria,p:Firmicutes,c:Clostridia,o:Clostridiales,f:Lachnospiraceae |
| OTU_489 | 0 | 1 | 0 | 2 | 0 | 2 | 2 | 0 | 1 | 2 | 2 | 2 | k:Bacteria,p:Actinobacteria,c:Actinobacteria,o:Coriobacteriales,f:Coriobacteriaceae |
| OTU_490 | 1 | 0 | 1 | 0 | 1 | 0 | 2 | 10 | 3 | 7 | 1 | 4 | k:Bacteria,p:Actinobacteria,c:Actinobacteria,o:Coriobacteriales,f:Coriobacteriaceae |
| OTU_491 | 2 | 8 | 5 | 11 | 3 | 3 | 0 | 0 | 0 | 0 | 0 | 0 | k:Bacteria,p:Firmicutes,c:Clostridia,o:Clostridiales,f:Lachnospiraceae |
| OTU_493 | 1 | 0 | 0 | 4 | 0 | 5 | 1 | 3 | 0 | 5 | 9 | 3 | k:Bacteria |
| OTU_494 | 0 | 1 | 0 | 0 | 0 | 0 | 1 | 28 | 0 | 0 | 0 | 0 | k:Bacteria,p:Firmicutes,c:Clostridia,o:Clostridiales,f:Lachnospiraceae |
| OTU_495 | 29 | 2 | 4 | 0 | 0 | 0 | 0 | 0 | 2 | 0 | 0 | 4 | k:Bacteria,p:Bacteroidetes,c:Bacteroidia,o:Bacteroidales,f:Prevotellaceae,g:Prevotella |
| OTU_496 | 5 | 4 | 4 | 3 | 1 | 0 | 0 | 0 | 0 | 2 | 0 | 0 | k:Bacteria,p:Firmicutes,c:Clostridia,o:Clostridiales,f:Lachnospiraceae,g:Clostridium_XlVa |
| OTU_497 | 1 | 1 | 3 | 3 | 2 | 6 | 1 | 2 | 4 | 1 | 2 | 10 | k:Bacteria,p:Firmicutes,c:Clostridia,o:Clostridiales |
| OTU_498 | 2 | 1 | 3 | 3 | 0 | 0 | 3 | 8 | 3 | 4 | 0 | 6 | k:Bacteria,p:Firmicutes,c:Clostridia,o:Clostridiales,f:Ruminococcaceae |
| OTU_499 | 3 | 0 | 22 | 0 | 0 | 3 | 1 | 0 | 1 | 0 | 4 | 0 | k:Bacteria,p:Firmicutes,c:Clostridia,o:Clostridiales,f:Lachnospiraceae,g:Lachnospiracea_incertae_sedis,s:Eubacterium_ventriosum |
| OTU_500 | 0 | 0 | 0 | 0 | 0 | 1 | 0 | 0 | 3 | 6 | 2 | 12 | k:Bacteria,p:Firmicutes,c:Clostridia,o:Clostridiales |
| OTU_501 | 3 | 2 | 4 | 1 | 3 | 0 | 2 | 0 | 5 | 2 | 2 | 13 | k:Bacteria,p:Firmicutes,c:Clostridia,o:Clostridiales,f:Lachnospiraceae |
| OTU_502 | 0 | 0 | 11 | 0 | 7 | 1 | 0 | 1 | 0 | 0 | 0 | 0 | k:Bacteria,p:Firmicutes,c:Clostridia,o:Clostridiales,f:Clostridiaceae_1,g:Clostridium_sensu_stricto |
| OTU_503 | 0 | 0 | 0 | 0 | 22 | 0 | 0 | 0 | 0 | 0 | 0 | 0 | k:Bacteria,p:Bacteroidetes,c:Bacteroidia,o:Bacteroidales,f:Rikenellaceae |
| OTU_504 | 12 | 2 | 2 | 2 | 0 | 2 | 0 | 0 | 1 | 0 | 0 | 0 | k:Bacteria,p:Firmicutes,c:Clostridia,o:Clostridiales,f:Ruminococcaceae |
| OTU_505 | 0 | 1 | 0 | 0 | 0 | 0 | 0 | 0 | 8 | 0 | 15 | 0 | k:Bacteria,p:Firmicutes,c:Clostridia,o:Clostridiales,f:Lachnospiraceae,g:Clostridium_XlVb,s:Clostridium_lactatifermentans |
| OTU_506 | 3 | 2 | 0 | 0 | 1 | 2 | 0 | 5 | 2 | 0 | 7 | 3 | k:Bacteria,p:Firmicutes,c:Clostridia,o:Clostridiales |
| OTU_507 | 13 | 28 | 0 | 0 | 0 | 5 | 0 | 1 | 0 | 0 | 1 | 3 | k:Bacteria |
| OTU_508 | 0 | 0 | 0 | 0 | 0 | 3 | 1 | 1 | 0 | 2 | 5 | 3 | k:Bacteria,p:Firmicutes,c:Clostridia,o:Clostridiales,f:Ruminococcaceae |
| OTU_509 | 0 | 0 | 0 | 0 | 0 | 0 | 22 | 0 | 0 | 0 | 2 | 0 | k:Bacteria,p:Firmicutes,c:Clostridia,o:Clostridiales,f:Lachnospiraceae |
| OTU_510 | 2 | 1 | 5 | 1 | 2 | 4 | 1 | 0 | 0 | 0 | 1 | 0 | k:Bacteria,p:Firmicutes,c:Clostridia,o:Clostridiales |
| OTU_511 | 0 | 0 | 0 | 23 | 0 | 0 | 0 | 0 | 0 | 0 | 0 | 0 | k:Bacteria,p:Firmicutes,c:Clostridia,o:Clostridiales,f:Lachnospiraceae |
| OTU_512 | 0 | 0 | 0 | 2 | 0 | 2 | 4 | 11 | 0 | 0 | 0 | 1 | k:Bacteria,p:Actinobacteria,c:Actinobacteria,o:Coriobacteriales,f:Coriobacteriaceae |
| OTU_513 | 10 | 15 | 23 | 26 | 13 | 13 | 13 | 28 | 28 | 14 | 5 | 14 | k:Bacteria,p:Firmicutes,c:Clostridia,o:Clostridiales,f:Lachnospiraceae,g:Clostridium_XlVa |
| OTU_514 | 0 | 0 | 0 | 3 | 5 | 5 | 1 | 2 | 0 | 3 | 1 | 4 | k:Bacteria,p:Firmicutes,c:Clostridia,o:Clostridiales,f:Ruminococcaceae,g:Sporobacter,s:Sporobacter_termitidis |
| OTU_515 | 10 | 7 | 9 | 0 | 1 | 2 | 1 | 1 | 1 | 1 | 1 | 3 | k:Bacteria,p:Firmicutes,c:Clostridia,o:Clostridiales |
| OTU_516 | 0 | 0 | 0 | 0 | 0 | 1 | 0 | 0 | 2 | 0 | 0 | 14 | k:Bacteria,p:Firmicutes,c:Clostridia,o:Clostridiales,f:Lachnospiraceae |
| OTU_517 | 0 | 1 | 0 | 0 | 0 | 1 | 4 | 3 | 0 | 4 | 6 | 0 | k:Bacteria,p:Firmicutes,c:Erysipelotrichia,o:Erysipelotrichales,f:Erysipelotrichaceae |
| OTU_518 | 5 | 0 | 0 | 0 | 7 | 0 | 1 | 3 | 2 | 0 | 2 | 1 | k:Bacteria,p:Bacteroidetes,c:Bacteroidia,o:Bacteroidales,f:Porphyromonadaceae |
| OTU_519 | 0 | 1 | 0 | 4 | 0 | 5 | 10 | 0 | 6 | 1 | 2 | 4 | k:Bacteria,p:Firmicutes,c:Clostridia,o:Clostridiales |
| OTU_520 | 10 | 8 | 5 | 8 | 10 | 7 | 13 | 16 | 7 | 13 | 21 | 18 | k:Bacteria,p:Firmicutes,c:Clostridia,o:Clostridiales,f:Ruminococcaceae |
| OTU_521 | 0 | 2 | 3 | 1 | 0 | 1 | 61 | 7 | 2 | 0 | 1 | 2 | k:Bacteria,p:Firmicutes,c:Clostridia,o:Clostridiales,f:Lachnospiraceae |
| OTU_522 | 4 | 6 | 2 | 5 | 5 | 3 | 13 | 19 | 9 | 6 | 7 | 26 | k:Bacteria,p:Firmicutes,c:Clostridia,o:Clostridiales,f:Lachnospiraceae |
| OTU_523 | 2 | 6 | 1 | 0 | 0 | 1 | 0 | 20 | 0 | 0 | 5 | 0 | k:Bacteria,p:Firmicutes,c:Clostridia,o:Clostridiales,f:Lachnospiraceae |
| OTU_524 | 1 | 0 | 0 | 0 | 0 | 18 | 0 | 0 | 0 | 0 | 0 | 0 | k:Bacteria,p:Firmicutes |
| OTU_525 | 0 | 7 | 2 | 16 | 9 | 1 | 8 | 19 | 7 | 0 | 8 | 17 | k:Bacteria,p:Firmicutes,c:Clostridia,o:Clostridiales,f:Lachnospiraceae,g:Acetatifactor,s:Acetatifactor_muris |
| OTU_526 | 6 | 35 | 2 | 1 | 0 | 8 | 0 | 0 | 2 | 2 | 2 | 1 | k:Bacteria |
| OTU_527 | 35 | 1 | 0 | 0 | 0 | 0 | 1 | 0 | 0 | 0 | 0 | 0 | k:Bacteria,p:Firmicutes,c:Clostridia,o:Clostridiales |
| OTU_528 | 1 | 17 | 0 | 0 | 6 | 4 | 2 | 1 | 12 | 1 | 5 | 1 | k:Bacteria,p:Proteobacteria,c:Betaproteobacteria,o:Burkholderiales,f:Sutterellaceae,g:Parasutterella,s:Parasutterella_excrementihominis |
| OTU_529 | 0 | 1 | 0 | 5 | 0 | 5 | 3 | 3 | 1 | 1 | 1 | 4 | k:Bacteria,p:Actinobacteria,c:Actinobacteria |
| OTU_530 | 0 | 0 | 0 | 2 | 1 | 3 | 1 | 5 | 1 | 3 | 0 | 5 | k:Bacteria,p:Firmicutes,c:Clostridia,o:Clostridiales,f:Ruminococcaceae |
| OTU_531 | 6 | 1 | 1 | 1 | 18 | 2 | 1 | 2 | 0 | 2 | 4 | 4 | k:Bacteria,p:Bacteroidetes,c:Bacteroidia,o:Bacteroidales,f:Rikenellaceae,g:Alistipes |
| OTU_532 | 4 | 1 | 0 | 1 | 0 | 3 | 1 | 1 | 2 | 0 | 1 | 1 | k:Bacteria,p:Firmicutes,c:Clostridia,o:Clostridiales,f:Ruminococcaceae |
| OTU_533 | 18 | 4 | 2 | 0 | 1 | 0 | 0 | 1 | 0 | 0 | 1 | 0 | k:Bacteria,p:Firmicutes,c:Clostridia,o:Clostridiales,f:Lachnospiraceae |
| OTU_534 | 2 | 8 | 0 | 4 | 1 | 3 | 3 | 2 | 1 | 1 | 2 | 4 | k:Bacteria,p:Firmicutes,c:Clostridia,o:Clostridiales,f:Ruminococcaceae |
| OTU_535 | 0 | 0 | 0 | 0 | 2 | 0 | 4 | 0 | 1 | 1 | 9 | 0 | k:Bacteria,p:Firmicutes,c:Clostridia,o:Clostridiales,f:Lachnospiraceae |
| OTU_536 | 5 | 16 | 3 | 10 | 1 | 9 | 12 | 5 | 13 | 11 | 13 | 14 | k:Bacteria,p:Firmicutes,c:Clostridia,o:Clostridiales,f:Lachnospiraceae |
| OTU_537 | 23 | 6 | 4 | 0 | 4 | 6 | 1 | 3 | 0 | 0 | 0 | 0 | k:Bacteria,p:Bacteroidetes,c:Bacteroidia,o:Bacteroidales,f:Porphyromonadaceae |
| OTU_538 | 13 | 0 | 0 | 0 | 0 | 0 | 0 | 0 | 0 | 0 | 0 | 0 | k:Bacteria |
| OTU_539 | 0 | 17 | 0 | 3 | 0 | 1 | 0 | 0 | 0 | 0 | 0 | 0 | k:Bacteria |
| OTU_540 | 1 | 0 | 0 | 5 | 3 | 6 | 0 | 0 | 0 | 0 | 0 | 0 | k:Bacteria,p:Firmicutes,c:Clostridia,o:Clostridiales |
| OTU_541 | 0 | 0 | 14 | 0 | 7 | 1 | 0 | 0 | 0 | 0 | 0 | 0 | k:Bacteria,p:Fusobacteria,c:Fusobacteriia,o:Fusobacteriales,f:Fusobacteriaceae,g:Fusobacterium,s:Fusobacterium_necrophorum_subsp._funduliforme |
| OTU_542 | 0 | 0 | 1 | 7 | 1 | 3 | 3 | 2 | 1 | 16 | 3 | 1 | k:Bacteria,p:Firmicutes,c:Clostridia,o:Clostridiales,f:Ruminococcaceae |
| OTU_543 | 0 | 1 | 0 | 2 | 0 | 5 | 0 | 2 | 0 | 0 | 3 | 4 | k:Bacteria,p:Firmicutes,c:Clostridia,o:Clostridiales |
| OTU_544 | 1 | 0 | 0 | 2 | 2 | 0 | 4 | 2 | 3 | 5 | 2 | 2 | k:Bacteria,p:Firmicutes,c:Clostridia,o:Clostridiales,f:Ruminococcaceae |
| OTU_545 | 0 | 0 | 1 | 1 | 0 | 12 | 1 | 0 | 0 | 5 | 2 | 0 | k:Bacteria |
| OTU_546 | 0 | 0 | 0 | 5 | 0 | 4 | 1 | 3 | 1 | 0 | 0 | 1 | k:Bacteria,p:Firmicutes,c:Clostridia,o:Clostridiales |
| OTU_547 | 0 | 3 | 0 | 0 | 0 | 3 | 0 | 10 | 0 | 12 | 1 | 0 | k:Bacteria,p:Firmicutes,c:Clostridia,o:Clostridiales |
| OTU_548 | 4 | 21 | 3 | 0 | 9 | 3 | 0 | 0 | 0 | 0 | 0 | 0 | k:Bacteria,p:Firmicutes,c:Clostridia,o:Clostridiales |
| OTU_549 | 2 | 4 | 1 | 3 | 2 | 9 | 5 | 7 | 1 | 12 | 12 | 18 | k:Bacteria,p:Firmicutes,c:Clostridia,o:Clostridiales,f:Lachnospiraceae |
| OTU_550 | 1 | 1 | 2 | 1 | 0 | 2 | 0 | 2 | 2 | 2 | 3 | 4 | k:Bacteria,p:Firmicutes,c:Clostridia,o:Clostridiales,f:Ruminococcaceae |
| OTU_551 | 13 | 15 | 9 | 28 | 35 | 6 | 6 | 3 | 6 | 7 | 8 | 24 | k:Bacteria,p:Firmicutes,c:Clostridia,o:Clostridiales,f:Ruminococcaceae,g:Clostridium_IV,s:Clostridium_leptum |
| OTU_552 | 0 | 0 | 0 | 0 | 0 | 0 | 1 | 8 | 0 | 6 | 0 | 0 | k:Bacteria,p:Firmicutes,c:Clostridia,o:Clostridiales |
| OTU_553 | 1 | 0 | 0 | 1 | 0 | 0 | 4 | 4 | 1 | 3 | 2 | 6 | k:Bacteria,p:Actinobacteria,c:Actinobacteria,o:Coriobacteriales,f:Coriobacteriaceae |
| OTU_554 | 0 | 0 | 0 | 4 | 0 | 0 | 1 | 3 | 5 | 2 | 1 | 8 | k:Bacteria,p:Firmicutes,c:Clostridia,o:Clostridiales,f:Lachnospiraceae |
| OTU_555 | 2 | 4 | 1 | 1 | 1 | 0 | 0 | 1 | 5 | 0 | 6 | 1 | k:Bacteria,p:Bacteroidetes,c:Bacteroidia,o:Bacteroidales,f:Porphyromonadaceae |
| OTU_556 | 0 | 0 | 26 | 1 | 11 | 2 | 0 | 0 | 0 | 0 | 0 | 2 | k:Bacteria,p:Proteobacteria,c:Gammaproteobacteria,o:Pseudomonadales,f:Moraxellaceae,g:Acinetobacter,s:Acinetobacter_baumannii |
| OTU_557 | 0 | 0 | 0 | 0 | 0 | 0 | 1 | 3 | 5 | 1 | 0 | 2 | k:Bacteria,p:Firmicutes,c:Clostridia,o:Clostridiales,f:Lachnospiraceae,g:Clostridium_XlVa |
| OTU_558 | 1 | 2 | 0 | 1 | 0 | 1 | 2 | 0 | 1 | 0 | 0 | 1 | k:Bacteria,p:Firmicutes,c:Clostridia,o:Clostridiales,f:Ruminococcaceae |
| OTU_559 | 2 | 1 | 1 | 0 | 0 | 1 | 0 | 4 | 1 | 2 | 5 | 1 | k:Bacteria,p:Firmicutes,c:Clostridia,o:Clostridiales |
| OTU_560 | 0 | 0 | 24 | 0 | 15 | 1 | 0 | 0 | 0 | 0 | 0 | 0 | k:Bacteria,p:Firmicutes,c:Bacilli,o:Bacillales |
| OTU_561 | 4 | 2 | 0 | 5 | 33 | 6 | 47 | 4 | 40 | 4 | 47 | 74 | k:Bacteria,p:Firmicutes,c:Clostridia,o:Clostridiales,f:Ruminococcaceae |
| OTU_562 | 7 | 6 | 1 | 0 | 8 | 0 | 0 | 1 | 1 | 0 | 0 | 0 | k:Bacteria,p:Bacteroidetes,c:Bacteroidia,o:Bacteroidales,f:Rikenellaceae,g:Alistipes |
| OTU_563 | 3 | 0 | 5 | 0 | 0 | 0 | 0 | 0 | 1 | 1 | 3 | 2 | k:Bacteria,p:Firmicutes,c:Clostridia,o:Clostridiales,f:Lachnospiraceae |
| OTU_564 | 0 | 0 | 0 | 0 | 0 | 1 | 1 | 1 | 7 | 1 | 8 | 7 | k:Bacteria,p:Firmicutes,c:Clostridia,o:Clostridiales,f:Ruminococcaceae |
| OTU_565 | 0 | 2 | 0 | 0 | 2 | 0 | 0 | 3 | 12 | 3 | 6 | 0 | k:Bacteria,p:Proteobacteria,c:Betaproteobacteria |
| OTU_566 | 0 | 1 | 4 | 14 | 2 | 1 | 0 | 0 | 1 | 0 | 1 | 0 | k:Bacteria,p:Firmicutes,c:Clostridia,o:Clostridiales,f:Ruminococcaceae,g:Clostridium_IV |
| OTU_567 | 19 | 10 | 3 | 8 | 16 | 4 | 9 | 2 | 5 | 7 | 8 | 8 | k:Bacteria,p:Bacteroidetes,c:Bacteroidia,o:Bacteroidales,f:Porphyromonadaceae |
| OTU_568 | 0 | 1 | 0 | 19 | 0 | 0 | 4 | 3 | 4 | 1 | 2 | 1 | k:Bacteria,p:Firmicutes,c:Clostridia,o:Clostridiales,f:Lachnospiraceae |
| OTU_569 | 6 | 3 | 0 | 2 | 0 | 4 | 1 | 6 | 3 | 0 | 0 | 0 | k:Bacteria |
| OTU_570 | 0 | 1 | 0 | 4 | 0 | 0 | 0 | 2 | 7 | 2 | 1 | 3 | k:Bacteria,p:Firmicutes,c:Clostridia,o:Clostridiales,f:Lachnospiraceae |
| OTU_571 | 1 | 4 | 5 | 2 | 2 | 3 | 0 | 0 | 0 | 0 | 7 | 0 | k:Bacteria,p:Firmicutes,c:Clostridia,o:Clostridiales,f:Lachnospiraceae |
| OTU_572 | 0 | 0 | 0 | 0 | 0 | 0 | 1 | 4 | 1 | 6 | 2 | 0 | k:Bacteria,p:Bacteroidetes,c:Bacteroidia,o:Bacteroidales |
| OTU_573 | 1 | 1 | 1 | 0 | 3 | 5 | 9 | 6 | 4 | 2 | 1 | 6 | k:Bacteria,p:Firmicutes |
| OTU_574 | 3 | 1 | 0 | 2 | 2 | 2 | 4 | 5 | 2 | 0 | 5 | 10 | k:Bacteria,p:Firmicutes,c:Clostridia,o:Clostridiales,f:Ruminococcaceae |
| OTU_575 | 0 | 0 | 0 | 0 | 0 | 0 | 2 | 1 | 1 | 5 | 0 | 3 | k:Bacteria,p:Firmicutes,c:Clostridia,o:Clostridiales,f:Lachnospiraceae |
| OTU_576 | 0 | 0 | 0 | 0 | 0 | 0 | 0 | 0 | 0 | 15 | 0 | 0 | k:Bacteria,p:Firmicutes,c:Clostridia,o:Clostridiales,f:Lachnospiraceae,g:Coprococcus,s:Coprococcus_eutactus |
| OTU_577 | 0 | 0 | 0 | 0 | 7 | 0 | 0 | 0 | 0 | 0 | 0 | 0 | k:Bacteria,p:Bacteroidetes,c:Sphingobacteriia,o:Sphingobacteriales,f:Sphingobacteriaceae,g:Pedobacter,s:Pedobacter_bauzanensis |
| OTU_578 | 1 | 2 | 2 | 8 | 4 | 0 | 0 | 0 | 1 | 1 | 12 | 0 | k:Bacteria,p:Firmicutes,c:Clostridia,o:Clostridiales,f:Lachnospiraceae |
| OTU_579 | 3 | 1 | 1 | 2 | 1 | 1 | 0 | 1 | 1 | 0 | 1 | 1 | k:Bacteria,p:Firmicutes |
| OTU_580 | 0 | 2 | 4 | 2 | 0 | 0 | 5 | 3 | 1 | 2 | 0 | 1 | k:Bacteria,p:Firmicutes,c:Clostridia,o:Clostridiales,f:Lachnospiraceae,g:Clostridium_XlVb |
| OTU_581 | 0 | 0 | 0 | 1 | 1 | 1 | 0 | 2 | 0 | 2 | 0 | 4 | k:Bacteria,p:Firmicutes,c:Clostridia,o:Clostridiales,f:Ruminococcaceae |
| OTU_582 | 0 | 0 | 14 | 0 | 3 | 1 | 0 | 1 | 0 | 0 | 0 | 0 | k:Bacteria,p:Firmicutes,c:Bacilli,o:Lactobacillales,f:Lactobacillaceae,g:Lactobacillus |
| OTU_583 | 2 | 1 | 7 | 3 | 4 | 1 | 7 | 4 | 3 | 0 | 0 | 3 | k:Bacteria,p:Firmicutes,c:Clostridia,o:Clostridiales,f:Lachnospiraceae |
| OTU_584 | 0 | 1 | 0 | 10 | 4 | 4 | 0 | 0 | 0 | 0 | 0 | 0 | k:Bacteria |
| OTU_585 | 0 | 0 | 0 | 2 | 1 | 8 | 0 | 0 | 1 | 1 | 0 | 3 | k:Bacteria,p:Proteobacteria,c:Alphaproteobacteria |
| OTU_586 | 1 | 3 | 0 | 0 | 1 | 1 | 1 | 1 | 0 | 1 | 0 | 1 | k:Bacteria,p:Firmicutes,c:Clostridia,o:Clostridiales,f:Ruminococcaceae |
| OTU_587 | 0 | 0 | 2 | 0 | 5 | 0 | 6 | 2 | 1 | 0 | 1 | 0 | k:Bacteria,p:Proteobacteria,c:Epsilonproteobacteria,o:Campylobacterales,f:Helicobacteraceae,g:Helicobacter,s:Helicobacter_rodentium |
| OTU_588 | 0 | 1 | 1 | 1 | 0 | 0 | 0 | 1 | 3 | 0 | 1 | 1 | k:Bacteria,p:Actinobacteria,c:Actinobacteria,o:Coriobacteriales,f:Coriobacteriaceae |
| OTU_589 | 2 | 1 | 3 | 5 | 1 | 3 | 0 | 3 | 0 | 0 | 0 | 0 | k:Bacteria,p:Firmicutes,c:Clostridia,o:Clostridiales,f:Ruminococcaceae |
| OTU_590 | 0 | 0 | 0 | 0 | 4 | 0 | 4 | 0 | 0 | 0 | 2 | 2 | k:Bacteria,p:Firmicutes,c:Clostridia,o:Clostridiales,f:Ruminococcaceae |
| OTU_591 | 0 | 0 | 0 | 1 | 0 | 0 | 1 | 4 | 1 | 2 | 1 | 3 | k:Bacteria,p:Actinobacteria,c:Actinobacteria,o:Coriobacteriales,f:Coriobacteriaceae |
| OTU_592 | 1 | 10 | 0 | 0 | 0 | 0 | 0 | 4 | 0 | 13 | 0 | 0 | k:Bacteria,p:Firmicutes,c:Clostridia,o:Clostridiales,f:Ruminococcaceae |
| OTU_593 | 3 | 0 | 3 | 0 | 1 | 1 | 3 | 1 | 0 | 4 | 2 | 5 | k:Bacteria,p:Firmicutes,c:Clostridia,o:Clostridiales,f:Ruminococcaceae |
| OTU_594 | 1 | 2 | 0 | 2 | 2 | 0 | 5 | 2 | 2 | 2 | 0 | 6 | k:Bacteria,p:Firmicutes,c:Clostridia,o:Clostridiales,f:Lachnospiraceae |
| OTU_595 | 0 | 3 | 1 | 6 | 1 | 3 | 10 | 3 | 1 | 3 | 2 | 5 | k:Bacteria,p:Firmicutes,c:Clostridia,o:Clostridiales,f:Lachnospiraceae |
| OTU_596 | 1 | 1 | 0 | 0 | 1 | 1 | 4 | 0 | 0 | 0 | 0 | 2 | k:Bacteria,p:Firmicutes,c:Clostridia,o:Clostridiales,f:Lachnospiraceae |
| OTU_597 | 0 | 0 | 0 | 0 | 0 | 1 | 0 | 0 | 7 | 1 | 1 | 3 | k:Bacteria,p:Actinobacteria,c:Actinobacteria,o:Coriobacteriales,f:Coriobacteriaceae,g:Collinsella,s:Collinsella_aerofaciens |
| OTU_598 | 2 | 37 | 10 | 11 | 5 | 14 | 22 | 169 | 11 | 8 | 7 | 15 | k:Bacteria |
| OTU_599 | 0 | 0 | 0 | 0 | 0 | 0 | 1 | 7 | 1 | 2 | 0 | 1 | k:Bacteria,p:Firmicutes,c:Clostridia,o:Clostridiales |
| OTU_600 | 12 | 0 | 0 | 0 | 0 | 0 | 0 | 0 | 0 | 0 | 0 | 0 | k:Bacteria,p:Firmicutes,c:Clostridia,o:Clostridiales,f:Lachnospiraceae |
| OTU_601 | 13 | 85 | 27 | 33 | 21 | 15 | 0 | 0 | 16 | 83 | 2 | 48 | k:Bacteria,p:Firmicutes,c:Clostridia,o:Clostridiales,f:Ruminococcaceae,g:Clostridium_IV |
| OTU_602 | 4 | 0 | 1 | 2 | 2 | 0 | 0 | 0 | 0 | 1 | 0 | 0 | k:Bacteria,p:Firmicutes,c:Clostridia,o:Clostridiales,f:Ruminococcaceae |
| OTU_603 | 0 | 0 | 1 | 0 | 0 | 4 | 1 | 0 | 1 | 0 | 0 | 7 | k:Bacteria,p:Firmicutes,c:Clostridia,o:Clostridiales |
| OTU_604 | 0 | 0 | 0 | 0 | 0 | 0 | 5 | 3 | 0 | 0 | 5 | 10 | k:Bacteria,p:Firmicutes,c:Clostridia,o:Clostridiales |
| OTU_605 | 13 | 4 | 0 | 0 | 6 | 0 | 0 | 0 | 0 | 0 | 0 | 0 | k:Bacteria |
| OTU_606 | 0 | 0 | 0 | 1 | 3 | 0 | 8 | 3 | 4 | 4 | 2 | 0 | k:Bacteria,p:Firmicutes,c:Clostridia,o:Clostridiales,f:Lachnospiraceae,g:Clostridium_XlVa |
| OTU_607 | 4 | 0 | 0 | 0 | 0 | 5 | 0 | 0 | 0 | 0 | 0 | 0 | k:Bacteria,p:Firmicutes,c:Clostridia,o:Clostridiales |
| OTU_608 | 0 | 0 | 0 | 2 | 0 | 0 | 3 | 4 | 0 | 1 | 0 | 0 | k:Bacteria,p:Firmicutes,c:Clostridia,o:Clostridiales,f:Ruminococcaceae |
| OTU_609 | 8 | 7 | 0 | 0 | 0 | 1 | 1 | 0 | 0 | 0 | 0 | 0 | k:Bacteria,p:Bacteroidetes,c:Bacteroidia,o:Bacteroidales,f:Prevotellaceae,g:Prevotella |
| OTU_610 | 0 | 0 | 4 | 0 | 6 | 0 | 0 | 0 | 0 | 0 | 0 | 0 | k:Bacteria,p:Proteobacteria,c:Alphaproteobacteria,o:Rhizobiales |
| OTU_611 | 0 | 0 | 0 | 0 | 2 | 2 | 0 | 1 | 0 | 1 | 1 | 0 | k:Bacteria,p:Firmicutes,c:Clostridia,o:Clostridiales,f:Ruminococcaceae |
| OTU_612 | 0 | 0 | 0 | 0 | 3 | 0 | 3 | 1 | 0 | 1 | 0 | 3 | k:Bacteria,p:Firmicutes,c:Clostridia |
| OTU_613 | 2 | 0 | 0 | 0 | 1 | 0 | 4 | 5 | 1 | 1 | 3 | 1 | k:Bacteria,p:Firmicutes,c:Clostridia,o:Clostridiales |
| OTU_614 | 36 | 68 | 62 | 10 | 51 | 25 | 10 | 5 | 14 | 16 | 9 | 27 | k:Bacteria,p:Firmicutes,c:Clostridia,o:Clostridiales |
| OTU_615 | 0 | 3 | 2 | 0 | 0 | 2 | 1 | 0 | 0 | 2 | 4 | 1 | k:Bacteria,p:Actinobacteria,c:Actinobacteria,o:Coriobacteriales,f:Coriobacteriaceae |
| OTU_616 | 2 | 2 | 1 | 0 | 0 | 2 | 2 | 0 | 0 | 4 | 1 | 0 | k:Bacteria,p:Firmicutes,c:Clostridia,o:Clostridiales,f:Ruminococcaceae |
| OTU_617 | 3 | 0 | 5 | 0 | 1 | 1 | 2 | 2 | 0 | 0 | 2 | 0 | k:Bacteria,p:Firmicutes,c:Clostridia,o:Clostridiales,f:Lachnospiraceae,g:Clostridium_XlVb,s:Clostridium_lactatifermentans |
| OTU_618 | 0 | 0 | 0 | 3 | 0 | 4 | 0 | 2 | 2 | 0 | 0 | 2 | k:Bacteria,p:Firmicutes,c:Bacilli,o:Lactobacillales,f:Aerococcaceae |
| OTU_619 | 1 | 0 | 0 | 2 | 0 | 1 | 1 | 1 | 2 | 1 | 2 | 1 | k:Bacteria,p:Firmicutes,c:Clostridia,o:Clostridiales,f:Ruminococcaceae,g:Clostridium_IV |
| OTU_620 | 1 | 0 | 0 | 1 | 0 | 0 | 0 | 4 | 2 | 2 | 9 | 3 | k:Bacteria,p:Firmicutes,c:Clostridia |
| OTU_621 | 1 | 0 | 1 | 0 | 5 | 0 | 1 | 2 | 2 | 0 | 0 | 0 | k:Bacteria,p:Firmicutes,c:Clostridia,o:Clostridiales |
| OTU_622 | 5 | 1 | 1 | 4 | 0 | 5 | 1 | 0 | 1 | 0 | 0 | 4 | k:Bacteria,p:Firmicutes,c:Negativicutes,o:Selenomonadales,f:Veillonellaceae,g:Veillonella |
| OTU_623 | 1 | 2 | 0 | 4 | 4 | 9 | 0 | 0 | 0 | 0 | 0 | 0 | k:Bacteria,p:Firmicutes,c:Clostridia,o:Clostridiales |
| OTU_624 | 0 | 2 | 14 | 0 | 0 | 23 | 17 | 0 | 3 | 7 | 37 | 12 | k:Bacteria |
| OTU_625 | 0 | 2 | 6 | 0 | 1 | 2 | 1 | 2 | 0 | 0 | 0 | 0 | k:Bacteria,p:Firmicutes,c:Clostridia,o:Clostridiales,f:Lachnospiraceae |
| OTU_626 | 2 | 0 | 0 | 2 | 1 | 3 | 2 | 0 | 1 | 0 | 0 | 1 | k:Bacteria,p:Firmicutes,c:Clostridia,o:Clostridiales,f:Ruminococcaceae |
| OTU_627 | 0 | 1 | 2 | 1 | 0 | 0 | 0 | 2 | 0 | 0 | 8 | 1 | k:Bacteria,p:Firmicutes,c:Clostridia,o:Clostridiales |
| OTU_628 | 13 | 12 | 3 | 5 | 2 | 3 | 5 | 10 | 3 | 5 | 6 | 2 | k:Bacteria,p:Firmicutes,c:Clostridia,o:Clostridiales,f:Ruminococcaceae |
| OTU_629 | 0 | 0 | 75 | 0 | 59 | 9 | 0 | 1 | 1 | 1 | 0 | 0 | k:Bacteria,p:Firmicutes,c:Bacilli,o:Lactobacillales,f:Enterococcaceae,g:Enterococcus,s:Enterococcus_saccharolyticus |
| OTU_630 | 1 | 1 | 2 | 1 | 0 | 2 | 1 | 0 | 1 | 1 | 0 | 1 | k:Bacteria,p:Actinobacteria,c:Actinobacteria,o:Coriobacteriales,f:Coriobacteriaceae |
| OTU_631 | 0 | 2 | 4 | 0 | 0 | 0 | 3 | 0 | 3 | 8 | 1 | 4 | k:Bacteria,p:Firmicutes,c:Clostridia,o:Clostridiales,f:Lachnospiraceae |
| OTU_632 | 0 | 0 | 0 | 11 | 0 | 1 | 0 | 0 | 0 | 0 | 0 | 0 | k:Bacteria,p:Firmicutes,c:Clostridia,o:Clostridiales,f:Lachnospiraceae |
| OTU_633 | 0 | 0 | 0 | 0 | 6 | 3 | 0 | 0 | 5 | 0 | 0 | 0 | k:Bacteria,p:Firmicutes,c:Erysipelotrichia,o:Erysipelotrichales,f:Erysipelotrichaceae,g:Erysipelotrichaceae_incertae_sedis,s:Eubacterium_dolichum |
| OTU_634 | 0 | 0 | 1 | 0 | 0 | 0 | 2 | 1 | 1 | 1 | 1 | 4 | k:Bacteria,p:Firmicutes,c:Clostridia,o:Clostridiales,f:Ruminococcaceae,g:Clostridium_IV |
| OTU_635 | 0 | 0 | 0 | 0 | 0 | 0 | 0 | 0 | 0 | 0 | 7 | 0 | k:Bacteria,p:Firmicutes,c:Clostridia,o:Clostridiales |
| OTU_636 | 1 | 0 | 0 | 1 | 1 | 2 | 7 | 7 | 2 | 0 | 2 | 0 | k:Bacteria |
| OTU_637 | 0 | 1 | 1 | 1 | 1 | 0 | 5 | 21 | 11 | 4 | 31 | 9 | k:Bacteria,p:Firmicutes,c:Clostridia,o:Clostridiales,f:Lachnospiraceae,g:Acetatifactor,s:Acetatifactor_muris |
| OTU_638 | 3 | 0 | 0 | 1 | 1 | 0 | 2 | 1 | 0 | 0 | 0 | 2 | k:Bacteria,p:Actinobacteria,c:Actinobacteria,o:Coriobacteriales,f:Coriobacteriaceae |
| OTU_639 | 1 | 0 | 0 | 5 | 0 | 5 | 0 | 0 | 0 | 0 | 0 | 0 | k:Bacteria,p:Firmicutes |
| OTU_640 | 0 | 0 | 0 | 1 | 1 | 1 | 2 | 2 | 0 | 5 | 0 | 2 | k:Bacteria,p:Firmicutes,c:Clostridia,o:Clostridiales,f:Ruminococcaceae,g:Ruminococcus |
| OTU_641 | 11 | 10 | 0 | 0 | 2 | 0 | 28 | 14 | 0 | 0 | 2 | 8 | k:Bacteria |
| OTU_642 | 2 | 0 | 0 | 1 | 3 | 3 | 0 | 1 | 1 | 1 | 0 | 2 | k:Bacteria,p:Firmicutes,c:Clostridia,o:Clostridiales,f:Ruminococcaceae |
| OTU_643 | 0 | 1 | 0 | 0 | 0 | 0 | 1 | 1 | 2 | 0 | 0 | 1 | k:Bacteria,p:Firmicutes,c:Clostridia,o:Clostridiales |
| OTU_644 | 0 | 3 | 0 | 2 | 0 | 13 | 1 | 0 | 1 | 1 | 0 | 0 | k:Bacteria,p:Actinobacteria,c:Actinobacteria,o:Coriobacteriales,f:Coriobacteriaceae,g:Olsenella |
| OTU_645 | 0 | 0 | 0 | 0 | 0 | 0 | 0 | 0 | 1 | 0 | 6 | 2 | k:Bacteria,p:Spirochaetes,c:Spirochaetia,o:Spirochaetales,f:Spirochaetaceae,g:Sphaerochaeta |
| OTU_646 | 0 | 4 | 0 | 0 | 0 | 0 | 0 | 1 | 0 | 0 | 0 | 1 | k:Bacteria |
| OTU_647 | 0 | 1 | 2 | 0 | 6 | 0 | 1 | 0 | 0 | 0 | 0 | 0 | k:Bacteria,p:Bacteroidetes,c:Bacteroidia,o:Bacteroidales,f:Porphyromonadaceae |
| OTU_648 | 0 | 0 | 5 | 0 | 1 | 0 | 1 | 0 | 1 | 1 | 4 | 2 | k:Bacteria |
| OTU_649 | 2 | 0 | 0 | 0 | 0 | 1 | 1 | 0 | 1 | 0 | 2 | 7 | k:Bacteria,p:Firmicutes,c:Clostridia,o:Clostridiales |
| OTU_650 | 0 | 0 | 0 | 0 | 3 | 0 | 2 | 1 | 0 | 0 | 0 | 4 | k:Bacteria |
| OTU_651 | 6 | 0 | 0 | 0 | 1 | 0 | 0 | 0 | 0 | 0 | 0 | 0 | k:Bacteria |
| OTU_652 | 1 | 4 | 3 | 5 | 0 | 1 | 0 | 0 | 0 | 4 | 1 | 0 | k:Bacteria,p:Firmicutes,c:Clostridia,o:Clostridiales,f:Lachnospiraceae,g:Clostridium_XlVa |
| OTU_653 | 1 | 0 | 0 | 3 | 1 | 0 | 9 | 0 | 0 | 0 | 4 | 0 | k:Bacteria,p:Firmicutes,c:Clostridia,o:Clostridiales,f:Lachnospiraceae,g:Acetatifactor,s:Acetatifactor_muris |
| OTU_654 | 1 | 2 | 0 | 0 | 0 | 0 | 0 | 0 | 0 | 3 | 2 | 3 | k:Bacteria,p:Firmicutes,c:Clostridia,o:Clostridiales |
| OTU_655 | 0 | 5 | 0 | 1 | 0 | 3 | 0 | 0 | 1 | 0 | 3 | 0 | k:Bacteria,p:Firmicutes,c:Clostridia,o:Clostridiales,f:Ruminococcaceae |
| OTU_656 | 0 | 2 | 1 | 4 | 0 | 0 | 1 | 1 | 0 | 2 | 3 | 1 | k:Bacteria,p:Firmicutes,c:Clostridia,o:Clostridiales,f:Lachnospiraceae |
| OTU_657 | 5 | 0 | 0 | 3 | 0 | 1 | 0 | 0 | 0 | 0 | 0 | 0 | k:Bacteria,p:Firmicutes,c:Clostridia |
| OTU_658 | 39 | 20 | 11 | 1 | 79 | 4 | 3 | 18 | 12 | 2 | 10 | 6 | k:Bacteria,p:Bacteroidetes,c:Bacteroidia,o:Bacteroidales,f:Bacteroidaceae,g:Bacteroides,s:Bacteroides_nordii |
| OTU_659 | 0 | 0 | 5 | 0 | 5 | 0 | 0 | 0 | 0 | 0 | 0 | 0 | k:Bacteria,p:Firmicutes,c:Bacilli,o:Bacillales,f:Paenibacillaceae_1,g:Brevibacillus |
| OTU_660 | 1 | 0 | 1 | 2 | 0 | 4 | 2 | 3 | 0 | 3 | 0 | 2 | k:Bacteria,p:Firmicutes,c:Clostridia,o:Clostridiales |
| OTU_661 | 0 | 14 | 1 | 0 | 0 | 0 | 1 | 0 | 0 | 0 | 2 | 0 | k:Bacteria,p:Firmicutes,c:Clostridia,o:Clostridiales,f:Lachnospiraceae |
| OTU_662 | 116 | 51 | 395 | 0 | 74 | 15 | 9 | 25 | 0 | 2 | 8 | 1 | k:Bacteria,p:Firmicutes,c:Clostridia,o:Clostridiales,f:Ruminococcaceae,g:Ruminococcus,s:Ruminococcus_bromii |
| OTU_663 | 0 | 0 | 0 | 0 | 1 | 1 | 5 | 0 | 0 | 2 | 3 | 1 | k:Bacteria,p:Firmicutes,c:Clostridia,o:Clostridiales,f:Lachnospiraceae |
| OTU_664 | 0 | 0 | 0 | 0 | 0 | 0 | 2 | 0 | 4 | 3 | 2 | 4 | k:Bacteria |
| OTU_665 | 7 | 97 | 6 | 0 | 0 | 0 | 0 | 11 | 1 | 0 | 1 | 22 | k:Bacteria,p:Firmicutes,c:Clostridia,o:Clostridiales,f:Lachnospiraceae |
| OTU_666 | 8 | 0 | 0 | 0 | 0 | 0 | 0 | 0 | 0 | 0 | 0 | 0 | k:Bacteria,p:Firmicutes,c:Clostridia,o:Clostridiales,f:Lachnospiraceae |
| OTU_667 | 0 | 1 | 0 | 0 | 0 | 0 | 1 | 0 | 1 | 2 | 4 | 3 | k:Bacteria,p:Firmicutes,c:Clostridia,o:Clostridiales |
| OTU_668 | 3 | 1 | 0 | 4 | 1 | 6 | 0 | 0 | 0 | 0 | 0 | 0 | k:Bacteria,p:Actinobacteria,c:Actinobacteria,o:Coriobacteriales,f:Coriobacteriaceae |
| OTU_669 | 0 | 1 | 1 | 0 | 0 | 1 | 0 | 1 | 1 | 0 | 1 | 1 | k:Bacteria,p:Actinobacteria,c:Actinobacteria,o:Coriobacteriales,f:Coriobacteriaceae |
| OTU_670 | 2 | 1 | 1 | 1 | 0 | 5 | 0 | 0 | 0 | 0 | 0 | 0 | k:Bacteria,p:Firmicutes,c:Clostridia,o:Clostridiales,f:Ruminococcaceae |
| OTU_671 | 0 | 0 | 0 | 0 | 0 | 0 | 0 | 0 | 0 | 15 | 3 | 0 | k:Bacteria,p:Firmicutes,c:Erysipelotrichia,o:Erysipelotrichales,f:Erysipelotrichaceae,g:Clostridium_XVIII |
| OTU_672 | 2 | 0 | 0 | 0 | 0 | 6 | 0 | 0 | 0 | 0 | 0 | 0 | k:Bacteria |
| OTU_673 | 0 | 1 | 0 | 2 | 0 | 1 | 0 | 0 | 0 | 2 | 0 | 0 | k:Bacteria,p:Proteobacteria |
| OTU_674 | 0 | 0 | 9 | 0 | 0 | 0 | 0 | 0 | 0 | 0 | 0 | 0 | k:Bacteria,p:Proteobacteria,c:Gammaproteobacteria,o:Pseudomonadales,f:Moraxellaceae,g:Enhydrobacter,s:Enhydrobacter_aerosaccus |
| OTU_675 | 0 | 0 | 0 | 0 | 0 | 1 | 0 | 0 | 0 | 2 | 0 | 2 | k:Bacteria,p:Firmicutes |
| OTU_676 | 0 | 0 | 3 | 0 | 0 | 5 | 0 | 0 | 0 | 0 | 2 | 0 | k:Bacteria,p:Firmicutes,c:Clostridia,o:Clostridiales |
| OTU_677 | 1 | 0 | 0 | 5 | 2 | 6 | 0 | 0 | 2 | 0 | 1 | 0 | k:Bacteria,p:Firmicutes,c:Clostridia,o:Clostridiales,f:Ruminococcaceae |
| OTU_678 | 0 | 0 | 0 | 0 | 0 | 0 | 0 | 0 | 0 | 2 | 1 | 3 | k:Bacteria,p:Firmicutes,c:Clostridia,o:Clostridiales |
| OTU_679 | 0 | 0 | 0 | 0 | 0 | 5 | 0 | 2 | 0 | 0 | 0 | 0 | k:Bacteria,p:Proteobacteria,c:Gammaproteobacteria,o:Pseudomonadales,f:Moraxellaceae,g:Psychrobacter,s:Psychrobacter_sanguinis |
| OTU_680 | 0 | 0 | 0 | 0 | 1 | 0 | 1 | 0 | 2 | 0 | 0 | 2 | k:Bacteria,p:Proteobacteria,c:Deltaproteobacteria,o:Desulfovibrionales |
| OTU_681 | 0 | 3 | 0 | 2 | 0 | 1 | 0 | 2 | 1 | 4 | 0 | 0 | k:Bacteria,p:Firmicutes,c:Clostridia,o:Clostridiales,f:Ruminococcaceae |
| OTU_682 | 0 | 0 | 4 | 0 | 0 | 1 | 0 | 3 | 0 | 2 | 3 | 2 | k:Bacteria,p:Firmicutes,c:Clostridia,o:Clostridiales,f:Ruminococcaceae,g:Oscillibacter |
| OTU_683 | 0 | 0 | 2 | 0 | 0 | 1 | 0 | 0 | 0 | 0 | 0 | 2 | k:Bacteria,p:Firmicutes,c:Clostridia,o:Clostridiales,f:Ruminococcaceae |
| OTU_684 | 0 | 0 | 3 | 0 | 2 | 0 | 0 | 0 | 0 | 0 | 0 | 0 | k:Bacteria,p:Firmicutes,c:Clostridia,o:Clostridiales,f:Ruminococcaceae,g:Clostridium_IV |
| OTU_685 | 0 | 0 | 0 | 0 | 0 | 0 | 2 | 1 | 1 | 0 | 4 | 1 | k:Bacteria,p:Firmicutes,c:Clostridia,o:Clostridiales,f:Lachnospiraceae |
| OTU_686 | 0 | 0 | 0 | 0 | 0 | 0 | 0 | 0 | 3 | 8 | 0 | 0 | k:Bacteria,p:Bacteroidetes,c:Bacteroidia,o:Bacteroidales,f:Porphyromonadaceae |
| OTU_687 | 0 | 0 | 0 | 0 | 0 | 0 | 0 | 2 | 2 | 1 | 0 | 0 | k:Bacteria,p:Firmicutes,c:Clostridia,o:Clostridiales |
| OTU_688 | 0 | 0 | 2 | 0 | 0 | 1 | 0 | 0 | 0 | 0 | 0 | 0 | k:Bacteria,p:Actinobacteria,c:Actinobacteria,o:Actinomycetales,f:Microbacteriaceae |
| OTU_689 | 1 | 1 | 1 | 13 | 1 | 205 | 40 | 7 | 13 | 2 | 2 | 74 | k:Bacteria,p:Firmicutes,c:Clostridia,o:Clostridiales,f:Lachnospiraceae |
| OTU_690 | 2 | 0 | 2 | 0 | 0 | 1 | 0 | 0 | 0 | 0 | 0 | 0 | k:Archaea,p:Euryarchaeota,c:Methanobacteria,o:Methanobacteriales,f:Methanobacteriaceae,g:Methanosphaera |
| OTU_691 | 1 | 0 | 0 | 7 | 6 | 0 | 8 | 9 | 9 | 3 | 13 | 1 | k:Bacteria,p:Firmicutes,c:Clostridia,o:Clostridiales,f:Ruminococcaceae |
| OTU_692 | 57 | 22 | 16 | 33 | 100 | 2 | 176 | 136 | 97 | 4 | 54 | 18 | k:Bacteria,p:Bacteroidetes,c:Bacteroidia,o:Bacteroidales |
| OTU_693 | 0 | 0 | 1 | 2 | 0 | 0 | 0 | 0 | 0 | 0 | 0 | 0 | k:Bacteria,p:Firmicutes,c:Clostridia,o:Clostridiales,f:Clostridiaceae_1,g:Clostridium_sensu_stricto |
| OTU_694 | 2 | 0 | 0 | 0 | 0 | 0 | 1 | 0 | 2 | 1 | 0 | 2 | k:Bacteria,p:Firmicutes,c:Clostridia,o:Clostridiales,f:Lachnospiraceae |
| OTU_695 | 3 | 4 | 0 | 2 | 3 | 1 | 5 | 8 | 0 | 1 | 0 | 0 | k:Bacteria,p:Firmicutes,c:Clostridia,o:Clostridiales,f:Ruminococcaceae,g:Butyricicoccus,s:Butyricicoccus_pullicaecorum |
| OTU_696 | 1 | 2 | 1 | 0 | 1 | 1 | 1 | 0 | 3 | 1 | 2 | 2 | k:Bacteria,p:Proteobacteria,c:Betaproteobacteria,o:Burkholderiales |
| OTU_697 | 0 | 2 | 0 | 4 | 1 | 0 | 1 | 2 | 0 | 2 | 2 | 2 | k:Bacteria,p:Actinobacteria,c:Actinobacteria,o:Coriobacteriales,f:Coriobacteriaceae |
| OTU_698 | 1 | 2 | 279 | 1 | 159 | 23 | 7 | 2 | 1 | 1 | 0 | 3 | k:Bacteria,p:Firmicutes,c:Bacilli,o:Bacillales,f:Bacillaceae_1,g:Bacillus |
| OTU_699 | 1 | 0 | 1 | 0 | 3 | 0 | 0 | 0 | 0 | 0 | 0 | 0 | k:Bacteria,p:Bacteroidetes,c:Bacteroidia,o:Bacteroidales,f:Porphyromonadaceae,g:Odoribacter |
| OTU_700 | 31 | 9 | 0 | 7 | 1 | 6 | 2 | 2 | 3 | 2 | 0 | 11 | k:Bacteria,p:Firmicutes,c:Clostridia,o:Clostridiales,f:Lachnospiraceae |
| OTU_701 | 0 | 0 | 2 | 1 | 0 | 1 | 0 | 2 | 1 | 1 | 0 | 0 | k:Bacteria,p:Firmicutes,c:Clostridia,o:Clostridiales |
| OTU_702 | 0 | 1 | 8 | 3 | 3 | 0 | 0 | 0 | 2 | 0 | 0 | 0 | k:Bacteria |
| OTU_703 | 0 | 0 | 0 | 7 | 0 | 0 | 0 | 0 | 2 | 0 | 0 | 0 | k:Bacteria,p:Firmicutes |
| OTU_704 | 0 | 1 | 0 | 0 | 0 | 0 | 0 | 3 | 0 | 0 | 0 | 0 | k:Bacteria |
| OTU_705 | 0 | 0 | 1 | 0 | 3 | 0 | 0 | 0 | 0 | 0 | 0 | 0 | k:Bacteria,p:Bacteroidetes,c:Flavobacteriia,o:Flavobacteriales,f:Flavobacteriaceae,g:Chishuiella,s:Chishuiella_changwenlii |
| OTU_706 | 0 | 6 | 5 | 0 | 0 | 0 | 0 | 0 | 0 | 0 | 0 | 0 | k:Bacteria,p:Firmicutes,c:Clostridia,o:Clostridiales |
| OTU_707 | 5 | 0 | 0 | 2 | 0 | 0 | 0 | 0 | 0 | 0 | 0 | 0 | k:Bacteria,p:Firmicutes,c:Clostridia,o:Clostridiales,f:Lachnospiraceae |
| OTU_708 | 3 | 0 | 0 | 0 | 0 | 0 | 0 | 0 | 0 | 0 | 0 | 0 | k:Bacteria |
| OTU_709 | 0 | 0 | 0 | 0 | 2 | 0 | 2 | 1 | 1 | 0 | 6 | 0 | k:Bacteria,p:Firmicutes,c:Clostridia,o:Clostridiales,f:Ruminococcaceae |
| OTU_710 | 1 | 0 | 0 | 0 | 0 | 0 | 0 | 0 | 1 | 0 | 3 | 1 | k:Bacteria,p:Firmicutes,c:Clostridia,o:Clostridiales |
| OTU_711 | 2 | 0 | 1 | 0 | 0 | 4 | 2 | 0 | 0 | 0 | 0 | 2 | k:Bacteria,p:Firmicutes,c:Clostridia,o:Clostridiales,f:Ruminococcaceae |
| OTU_712 | 7 | 23 | 11 | 0 | 6 | 8 | 1 | 0 | 0 | 1 | 3 | 0 | k:Bacteria,p:Firmicutes,c:Clostridia,o:Clostridiales |
| OTU_713 | 1 | 1 | 1 | 0 | 0 | 1 | 3 | 0 | 1 | 2 | 7 | 0 | k:Bacteria,p:Firmicutes,c:Clostridia,o:Clostridiales,f:Lachnospiraceae |
| OTU_714 | 2 | 1 | 0 | 0 | 0 | 3 | 0 | 0 | 0 | 0 | 0 | 1 | k:Bacteria,p:Actinobacteria,c:Actinobacteria,o:Coriobacteriales,f:Coriobacteriaceae |
| OTU_715 | 0 | 1 | 0 | 0 | 0 | 0 | 0 | 0 | 3 | 1 | 3 | 4 | k:Bacteria,p:Firmicutes,c:Clostridia,o:Clostridiales,f:Lachnospiraceae |
| OTU_716 | 0 | 0 | 0 | 0 | 4 | 0 | 0 | 0 | 0 | 0 | 0 | 0 | k:Bacteria,p:Proteobacteria,c:Alphaproteobacteria |
| OTU_717 | 0 | 0 | 0 | 0 | 0 | 0 | 0 | 5 | 0 | 1 | 1 | 0 | k:Bacteria,p:Firmicutes,c:Clostridia,o:Clostridiales,f:Ruminococcaceae |
| OTU_718 | 0 | 0 | 0 | 1 | 0 | 1 | 1 | 1 | 1 | 3 | 2 | 0 | k:Bacteria,p:Firmicutes,c:Clostridia,o:Clostridiales,f:Lachnospiraceae |
| OTU_719 | 0 | 0 | 0 | 0 | 0 | 3 | 1 | 0 | 0 | 1 | 1 | 4 | k:Bacteria,p:Firmicutes,c:Clostridia,o:Clostridiales,f:Ruminococcaceae |
| OTU_720 | 0 | 3 | 0 | 1 | 1 | 0 | 0 | 0 | 0 | 1 | 0 | 0 | k:Bacteria,p:Firmicutes,c:Clostridia,o:Clostridiales,f:Lachnospiraceae |
| OTU_721 | 0 | 0 | 2 | 0 | 3 | 0 | 0 | 0 | 0 | 0 | 0 | 0 | k:Bacteria,p:Proteobacteria,c:Gammaproteobacteria,o:Oceanospirillales,f:Oceanospirillaceae,g:Marinomonas |
| OTU_722 | 0 | 0 | 5 | 0 | 4 | 1 | 0 | 0 | 0 | 0 | 0 | 0 | k:Bacteria,p:Firmicutes,c:Bacilli,o:Bacillales,f:Paenibacillaceae_1,g:Paenibacillus |
| OTU_723 | 1 | 0 | 1 | 0 | 0 | 0 | 2 | 2 | 0 | 2 | 0 | 1 | k:Bacteria,p:Bacteroidetes,c:Bacteroidia,o:Bacteroidales,f:Porphyromonadaceae |
| OTU_724 | 0 | 0 | 0 | 0 | 0 | 1 | 10 | 3 | 0 | 0 | 3 | 5 | k:Bacteria,p:Firmicutes,c:Clostridia,o:Clostridiales,f:Lachnospiraceae |
| OTU_725 | 5 | 8 | 74 | 0 | 0 | 1 | 0 | 0 | 1 | 0 | 0 | 1 | k:Bacteria,p:Firmicutes,c:Clostridia,o:Clostridiales,f:Lachnospiraceae |
| OTU_726 | 3 | 0 | 0 | 9 | 5 | 0 | 2 | 0 | 0 | 0 | 0 | 0 | k:Bacteria,p:Firmicutes,c:Clostridia,o:Clostridiales,f:Lachnospiraceae |
| OTU_727 | 3 | 0 | 0 | 0 | 0 | 2 | 0 | 0 | 0 | 1 | 1 | 4 | k:Bacteria,p:Firmicutes,c:Clostridia,o:Clostridiales,f:Ruminococcaceae |
| OTU_728 | 0 | 0 | 0 | 0 | 1 | 4 | 0 | 0 | 0 | 0 | 0 | 0 | k:Bacteria,p:Proteobacteria,c:Alphaproteobacteria |
| OTU_729 | 0 | 0 | 0 | 0 | 0 | 0 | 0 | 1 | 1 | 0 | 9 | 2 | k:Bacteria,p:Firmicutes,c:Clostridia,o:Clostridiales,f:Lachnospiraceae |
| OTU_730 | 0 | 0 | 1 | 0 | 2 | 1 | 0 | 0 | 0 | 0 | 0 | 0 | k:Bacteria,p:Proteobacteria,c:Alphaproteobacteria,o:Rhodospirillales,g:Reyranella,s:Reyranella_massiliensis |
| OTU_731 | 0 | 0 | 4 | 0 | 0 | 0 | 0 | 0 | 0 | 0 | 0 | 1 | k:Bacteria,p:Firmicutes,c:Clostridia,o:Clostridiales,f:Ruminococcaceae,g:Clostridium_IV |
| OTU_732 | 0 | 0 | 1 | 1 | 1 | 0 | 0 | 0 | 0 | 0 | 2 | 1 | k:Bacteria,p:Firmicutes,c:Clostridia,o:Clostridiales,f:Lachnospiraceae |
| OTU_733 | 28 | 111 | 151 | 61 | 18 | 23 | 42 | 62 | 26 | 29 | 13 | 45 | k:Bacteria,p:Firmicutes,c:Clostridia,o:Clostridiales,f:Lachnospiraceae |
| OTU_734 | 0 | 0 | 0 | 0 | 1 | 3 | 1 | 0 | 0 | 0 | 0 | 0 | k:Bacteria |
| OTU_735 | 0 | 1 | 1 | 0 | 0 | 0 | 1 | 0 | 0 | 4 | 0 | 0 | k:Bacteria,p:Firmicutes |
| OTU_736 | 0 | 0 | 1 | 1 | 0 | 1 | 1 | 0 | 0 | 0 | 3 | 0 | k:Bacteria,p:Firmicutes,c:Clostridia,o:Clostridiales,f:Lachnospiraceae |
| OTU_737 | 0 | 0 | 1 | 0 | 1 | 0 | 0 | 0 | 0 | 0 | 0 | 0 | k:Bacteria,p:Proteobacteria,c:Gammaproteobacteria,o:Xanthomonadales,f:Xanthomonadaceae,g:Stenotrophomonas |
| OTU_738 | 5 | 0 | 0 | 0 | 0 | 10 | 0 | 0 | 10 | 3 | 12 | 22 | k:Bacteria,p:Firmicutes,c:Clostridia,o:Clostridiales,f:Lachnospiraceae |
| OTU_739 | 1 | 200 | 0 | 0 | 0 | 12 | 0 | 0 | 0 | 0 | 0 | 0 | k:Bacteria |
| OTU_740 | 0 | 0 | 1 | 0 | 0 | 0 | 0 | 0 | 0 | 0 | 0 | 2 | k:Bacteria |
| OTU_741 | 5 | 15 | 22 | 10 | 8 | 1 | 43 | 18 | 30 | 8 | 3 | 16 | k:Bacteria,p:Firmicutes,c:Clostridia,o:Clostridiales,f:Lachnospiraceae |
| OTU_742 | 11 | 25 | 30 | 8 | 47 | 39 | 9 | 100 | 31 | 9 | 18 | 43 | k:Bacteria,p:Firmicutes,c:Clostridia,o:Clostridiales,f:Lachnospiraceae,g:Clostridium_XlVa |
| OTU_743 | 0 | 0 | 1 | 0 | 0 | 2 | 0 | 0 | 0 | 0 | 5 | 0 | k:Bacteria,p:Proteobacteria,c:Gammaproteobacteria,o:Enterobacteriales,f:Enterobacteriaceae,g:Proteus |
| OTU_744 | 0 | 0 | 3 | 0 | 4 | 0 | 0 | 0 | 0 | 0 | 0 | 0 | k:Bacteria,p:Proteobacteria,c:Alphaproteobacteria,o:Rhizobiales,f:Phyllobacteriaceae,g:Chelativorans,s:Chelativorans_multitrophicus |
| OTU_745 | 9 | 34 | 42 | 0 | 0 | 0 | 0 | 1 | 1 | 6 | 0 | 0 | k:Bacteria,p:Firmicutes,c:Clostridia,o:Clostridiales,f:Lachnospiraceae |
| OTU_746 | 3 | 5 | 0 | 6 | 21 | 79 | 1 | 9 | 3 | 0 | 15 | 84 | k:Bacteria,p:Firmicutes,c:Clostridia,o:Clostridiales |
| OTU_747 | 0 | 0 | 2 | 0 | 2 | 0 | 0 | 0 | 0 | 0 | 0 | 0 | k:Bacteria,p:Proteobacteria,c:Gammaproteobacteria,o:Xanthomonadales,f:Xanthomonadaceae,g:Stenotrophomonas |
| OTU_748 | 1 | 1 | 0 | 1 | 0 | 0 | 1 | 0 | 0 | 1 | 1 | 1 | k:Bacteria,p:Firmicutes,c:Clostridia,o:Clostridiales |
| OTU_749 | 0 | 0 | 0 | 0 | 1 | 0 | 0 | 2 | 2 | 0 | 2 | 2 | k:Bacteria,p:Firmicutes,c:Clostridia,o:Clostridiales,f:Ruminococcaceae |
| OTU_750 | 79 | 67 | 22 | 35 | 32 | 53 | 202 | 189 | 93 | 172 | 28 | 81 | k:Bacteria,p:Firmicutes,c:Clostridia,o:Clostridiales,f:Ruminococcaceae,g:Clostridium_IV |
| OTU_751 | 0 | 0 | 0 | 1 | 0 | 0 | 0 | 0 | 0 | 1 | 0 | 3 | k:Bacteria,p:Firmicutes,c:Clostridia,o:Clostridiales,f:Ruminococcaceae |
| OTU_752 | 0 | 0 | 1 | 0 | 3 | 1 | 0 | 0 | 1 | 2 | 2 | 0 | k:Bacteria,p:Tenericutes,c:Mollicutes,o:Mycoplasmatales,f:Mycoplasmataceae,g:Mycoplasma |
| OTU_753 | 0 | 0 | 1 | 0 | 0 | 0 | 0 | 0 | 0 | 1 | 0 | 1 | k:Bacteria,p:Firmicutes,c:Clostridia,o:Clostridiales |
| OTU_754 | 0 | 1 | 0 | 0 | 0 | 0 | 0 | 0 | 0 | 0 | 1 | 1 | k:Bacteria,p:Firmicutes,c:Clostridia,o:Clostridiales |
| OTU_755 | 0 | 0 | 0 | 0 | 0 | 0 | 0 | 1 | 0 | 0 | 3 | 0 | k:Bacteria,p:Firmicutes,c:Clostridia,o:Clostridiales,f:Lachnospiraceae |
| OTU_756 | 0 | 0 | 0 | 1 | 1 | 2 | 0 | 0 | 0 | 0 | 0 | 10 | k:Bacteria,p:Deferribacteres,c:Deferribacteres,o:Deferribacterales,f:Deferribacteraceae,g:Mucispirillum,s:Mucispirillum_schaedleri |
| OTU_757 | 0 | 0 | 0 | 1 | 0 | 0 | 0 | 0 | 0 | 1 | 1 | 0 | k:Bacteria,p:Proteobacteria,c:Alphaproteobacteria |
| OTU_758 | 0 | 0 | 3 | 0 | 0 | 1 | 0 | 0 | 0 | 0 | 0 | 0 | k:Bacteria,p:Firmicutes,c:Clostridia,o:Clostridiales,f:Clostridiales_Incertae_Sedis_XIII,g:Mogibacterium |
| OTU_759 | 1 | 0 | 0 | 1 | 4 | 0 | 0 | 0 | 0 | 0 | 0 | 0 | k:Bacteria,p:Firmicutes,c:Clostridia,o:Clostridiales |
| OTU_760 | 0 | 1 | 0 | 0 | 0 | 3 | 0 | 0 | 0 | 0 | 0 | 1 | k:Bacteria,p:Firmicutes,c:Erysipelotrichia,o:Erysipelotrichales,f:Erysipelotrichaceae |
| OTU_761 | 1 | 0 | 0 | 0 | 0 | 2 | 0 | 0 | 1 | 1 | 1 | 0 | k:Bacteria,p:Firmicutes |
| OTU_762 | 2 | 0 | 0 | 1 | 0 | 0 | 1 | 0 | 0 | 0 | 10 | 0 | k:Bacteria,p:Firmicutes,c:Clostridia,o:Clostridiales,f:Lachnospiraceae |
| OTU_763 | 0 | 0 | 0 | 2 | 1 | 0 | 0 | 0 | 0 | 0 | 1 | 0 | k:Bacteria,p:Proteobacteria |
| OTU_764 | 0 | 0 | 1 | 0 | 6 | 0 | 0 | 0 | 0 | 0 | 0 | 0 | k:Bacteria,p:Bacteroidetes,c:Flavobacteriia,o:Flavobacteriales,f:Flavobacteriaceae |
| OTU_765 | 2 | 3 | 14 | 0 | 0 | 2 | 1 | 0 | 0 | 5 | 0 | 12 | k:Bacteria,p:Firmicutes,c:Clostridia,o:Clostridiales,f:Lachnospiraceae |
| OTU_766 | 0 | 0 | 4 | 14 | 0 | 1 | 37 | 2 | 0 | 1 | 20 | 1 | k:Bacteria,p:Firmicutes,c:Clostridia,o:Clostridiales,f:Lachnospiraceae,g:Clostridium_XlVa |
| OTU_767 | 21 | 17 | 14 | 16 | 11 | 3 | 15 | 19 | 4 | 11 | 2 | 21 | k:Bacteria,p:Firmicutes,c:Clostridia,o:Clostridiales,f:Lachnospiraceae |
| OTU_768 | 0 | 0 | 0 | 0 | 0 | 1 | 0 | 0 | 1 | 0 | 1 | 2 | k:Bacteria,p:Firmicutes,c:Clostridia,o:Clostridiales,f:Lachnospiraceae |
| OTU_769 | 1 | 1 | 1 | 1 | 0 | 1 | 0 | 0 | 2 | 0 | 1 | 1 | k:Bacteria,p:Firmicutes,c:Clostridia,o:Clostridiales,f:Lachnospiraceae |
| OTU_770 | 0 | 0 | 0 | 1 | 0 | 0 | 2 | 3 | 1 | 0 | 0 | 0 | k:Bacteria,p:Firmicutes,c:Clostridia,o:Clostridiales,f:Lachnospiraceae |
| OTU_771 | 0 | 0 | 0 | 0 | 2 | 0 | 0 | 0 | 0 | 0 | 0 | 0 | k:Bacteria,p:Firmicutes,c:Clostridia,o:Clostridiales,f:Lachnospiraceae |
| OTU_772 | 0 | 3 | 0 | 0 | 0 | 0 | 0 | 0 | 1 | 0 | 0 | 0 | k:Bacteria,p:Firmicutes,c:Clostridia,o:Clostridiales,f:Ruminococcaceae |
| OTU_773 | 4 | 25 | 1 | 1 | 3 | 253 | 1 | 0 | 0 | 2 | 0 | 1 | k:Bacteria,p:Firmicutes,c:Clostridia,o:Clostridiales,f:Ruminococcaceae,g:Ruminococcus |
| OTU_774 | 0 | 0 | 0 | 2 | 0 | 1 | 0 | 0 | 0 | 0 | 0 | 0 | k:Bacteria,p:Firmicutes,c:Clostridia,o:Clostridiales,f:Ruminococcaceae |
| OTU_775 | 26 | 5 | 1 | 1 | 66 | 3 | 11 | 26 | 58 | 0 | 95 | 11 | k:Bacteria,p:Bacteroidetes,c:Bacteroidia,o:Bacteroidales,f:Bacteroidaceae,g:Bacteroides,s:Bacteroides_sartorii |
| OTU_776 | 2 | 1 | 1 | 7 | 0 | 2 | 2 | 3 | 3 | 1 | 4 | 4 | k:Bacteria,p:Firmicutes,c:Clostridia,o:Clostridiales,f:Ruminococcaceae,g:Oscillibacter |
| OTU_777 | 1 | 7 | 0 | 0 | 0 | 0 | 0 | 0 | 0 | 0 | 0 | 0 | k:Bacteria,p:Firmicutes,c:Clostridia,o:Clostridiales,f:Lachnospiraceae |
| OTU_778 | 0 | 0 | 0 | 0 | 2 | 0 | 0 | 1 | 0 | 0 | 4 | 1 | k:Bacteria,p:Firmicutes,c:Clostridia,o:Clostridiales |
| OTU_779 | 0 | 4 | 0 | 0 | 3 | 1 | 2 | 0 | 1 | 0 | 2 | 1 | k:Bacteria |
| OTU_780 | 0 | 0 | 1 | 0 | 0 | 1 | 2 | 0 | 1 | 0 | 0 | 1 | k:Bacteria,p:Firmicutes,c:Erysipelotrichia,o:Erysipelotrichales,f:Erysipelotrichaceae,g:Clostridium_XVIII |
| OTU_781 | 0 | 1 | 1 | 2 | 1 | 8 | 0 | 0 | 2 | 0 | 21 | 0 | k:Bacteria,p:Firmicutes,c:Clostridia,o:Clostridiales |
| OTU_782 | 2 | 0 | 1 | 0 | 1 | 1 | 1 | 0 | 0 | 1 | 1 | 3 | k:Bacteria,p:Firmicutes,c:Bacilli,o:Bacillales,f:Staphylococcaceae,g:Jeotgalicoccus,s:Jeotgalicoccus_nanhaiensis |
| OTU_783 | 1 | 5 | 0 | 1 | 0 | 0 | 1 | 0 | 0 | 0 | 0 | 0 | k:Bacteria,p:Firmicutes,c:Clostridia,o:Clostridiales |
| OTU_784 | 0 | 0 | 4 | 0 | 0 | 0 | 0 | 0 | 0 | 0 | 0 | 0 | k:Bacteria,p:Proteobacteria,c:Alphaproteobacteria |
| OTU_785 | 1 | 0 | 0 | 0 | 0 | 0 | 0 | 2 | 0 | 0 | 1 | 0 | k:Bacteria,p:Firmicutes,c:Clostridia,o:Clostridiales |
| OTU_786 | 0 | 2 | 0 | 0 | 0 | 0 | 0 | 0 | 0 | 0 | 0 | 0 | k:Bacteria,p:Firmicutes,c:Clostridia,o:Clostridiales,f:Lachnospiraceae |
| OTU_787 | 0 | 3 | 0 | 0 | 0 | 1 | 0 | 0 | 0 | 0 | 0 | 0 | k:Bacteria,p:Firmicutes,c:Clostridia,o:Clostridiales |
| OTU_788 | 0 | 0 | 0 | 0 | 0 | 0 | 0 | 1 | 0 | 2 | 0 | 0 | k:Bacteria,p:Firmicutes,c:Clostridia,o:Clostridiales,f:Ruminococcaceae |
| OTU_789 | 0 | 0 | 0 | 0 | 0 | 1 | 0 | 0 | 4 | 0 | 5 | 3 | k:Bacteria,p:Firmicutes,c:Clostridia,o:Clostridiales,f:Ruminococcaceae,g:Anaerotruncus,s:Anaerotruncus_colihominis |
| OTU_790 | 3 | 0 | 0 | 0 | 1 | 0 | 1 | 0 | 9 | 4 | 1 | 58 | k:Bacteria,p:Firmicutes,c:Clostridia,o:Clostridiales,f:Ruminococcaceae |
| OTU_791 | 13 | 6 | 23 | 18 | 0 | 0 | 24 | 25 | 21 | 56 | 3 | 1 | k:Bacteria,p:Firmicutes,c:Clostridia,o:Clostridiales,f:Lachnospiraceae |
| OTU_792 | 0 | 0 | 3 | 0 | 2 | 1 | 1 | 0 | 0 | 0 | 0 | 0 | k:Bacteria,p:Actinobacteria,c:Actinobacteria,o:Actinomycetales,f:Micrococcaceae,g:Arthrobacter |
| OTU_793 | 0 | 1 | 7 | 0 | 1 | 0 | 3 | 0 | 1 | 1 | 17 | 1 | k:Bacteria,p:Firmicutes,c:Clostridia,o:Clostridiales,f:Ruminococcaceae,g:Ruminococcus,s:Ruminococcus_callidus |
| OTU_794 | 0 | 0 | 0 | 0 | 0 | 0 | 0 | 0 | 6 | 0 | 0 | 0 | k:Bacteria,p:Firmicutes,c:Clostridia,o:Clostridiales |
| OTU_795 | 4 | 3 | 1 | 0 | 0 | 1 | 0 | 2 | 0 | 2 | 1 | 0 | k:Bacteria,p:Firmicutes,c:Clostridia,o:Clostridiales |
| OTU_796 | 1 | 0 | 0 | 0 | 0 | 0 | 0 | 2 | 0 | 0 | 0 | 0 | k:Bacteria,p:Firmicutes,c:Clostridia,o:Clostridiales,f:Lachnospiraceae |
| OTU_797 | 0 | 0 | 0 | 0 | 1 | 0 | 1 | 1 | 0 | 0 | 1 | 0 | k:Bacteria,p:Bacteroidetes,c:Bacteroidia,o:Bacteroidales,f:Porphyromonadaceae,g:Parabacteroides,s:Parabacteroides_goldsteinii |
| OTU_798 | 0 | 1 | 0 | 0 | 0 | 0 | 0 | 0 | 2 | 1 | 0 | 10 | k:Bacteria,p:Firmicutes,c:Clostridia,o:Clostridiales,f:Lachnospiraceae,g:Blautia,s:Blautia_wexlerae |
| OTU_799 | 0 | 2 | 1 | 0 | 0 | 0 | 3 | 5 | 1 | 0 | 2 | 0 | k:Bacteria,p:Firmicutes,c:Clostridia,o:Clostridiales,f:Lachnospiraceae,g:Blautia |
| OTU_800 | 0 | 0 | 0 | 1 | 0 | 0 | 1 | 1 | 0 | 1 | 0 | 0 | k:Bacteria,p:Firmicutes,c:Clostridia,o:Clostridiales,f:Ruminococcaceae,g:Clostridium_III |
| OTU_801 | 0 | 0 | 5 | 0 | 0 | 0 | 0 | 0 | 1 | 1 | 0 | 0 | k:Bacteria,p:Firmicutes,c:Clostridia,o:Clostridiales,f:Lachnospiraceae |
| OTU_802 | 0 | 0 | 0 | 1 | 0 | 2 | 0 | 0 | 0 | 0 | 0 | 1 | k:Bacteria,p:Firmicutes |
| OTU_803 | 0 | 4 | 0 | 0 | 0 | 0 | 0 | 0 | 0 | 0 | 0 | 0 | k:Bacteria,p:Firmicutes,c:Clostridia,o:Clostridiales,f:Lachnospiraceae |
| OTU_804 | 2 | 0 | 0 | 1 | 0 | 0 | 0 | 0 | 0 | 0 | 0 | 0 | k:Bacteria,p:Firmicutes,c:Clostridia,o:Clostridiales |
| OTU_805 | 0 | 0 | 0 | 0 | 0 | 0 | 0 | 0 | 0 | 0 | 1 | 1 | k:Bacteria,p:Firmicutes,c:Clostridia,o:Clostridiales,f:Ruminococcaceae |
| OTU_806 | 35 | 5 | 4 | 15 | 0 | 4 | 2 | 0 | 4 | 4 | 0 | 11 | k:Bacteria,p:Firmicutes,c:Clostridia,o:Clostridiales,f:Lachnospiraceae |
| OTU_807 | 0 | 1 | 1 | 0 | 1 | 0 | 0 | 0 | 0 | 1 | 0 | 0 | k:Bacteria,p:Firmicutes,c:Clostridia,o:Clostridiales,f:Lachnospiraceae |
| OTU_808 | 1 | 2 | 0 | 1 | 3 | 4 | 43 | 4 | 16 | 1 | 9 | 2 | k:Bacteria |
| OTU_809 | 0 | 0 | 0 | 0 | 0 | 1 | 0 | 0 | 0 | 0 | 0 | 2 | k:Bacteria,p:Firmicutes,c:Clostridia,o:Clostridiales |
| OTU_810 | 0 | 3 | 0 | 0 | 0 | 1 | 0 | 2 | 0 | 3 | 1 | 0 | k:Bacteria,p:Firmicutes,c:Clostridia,o:Clostridiales,f:Ruminococcaceae |
| OTU_811 | 0 | 0 | 1 | 2 | 1 | 0 | 0 | 0 | 0 | 0 | 0 | 0 | k:Bacteria,p:Actinobacteria,c:Actinobacteria,o:Coriobacteriales,f:Coriobacteriaceae,g:Enterorhabdus,s:Enterorhabdus_mucosicola |
| OTU_812 | 0 | 0 | 0 | 4 | 0 | 0 | 0 | 0 | 0 | 0 | 0 | 0 | k:Bacteria,p:Proteobacteria,c:Betaproteobacteria,o:Burkholderiales,f:Oxalobacteraceae |
| OTU_813 | 2 | 0 | 0 | 0 | 0 | 0 | 0 | 0 | 0 | 0 | 0 | 0 | k:Bacteria,p:Firmicutes,c:Clostridia,o:Clostridiales,f:Lachnospiraceae |
| OTU_814 | 0 | 1 | 3 | 0 | 0 | 0 | 0 | 0 | 0 | 0 | 0 | 0 | k:Bacteria,p:Firmicutes,c:Clostridia,o:Clostridiales,f:Lachnospiraceae |
| OTU_815 | 0 | 0 | 0 | 0 | 1 | 0 | 0 | 1 | 0 | 0 | 7 | 4 | k:Bacteria,p:Actinobacteria,c:Actinobacteria,o:Actinomycetales,f:Corynebacteriaceae,g:Corynebacterium |
| OTU_816 | 1 | 2 | 9 | 12 | 3 | 3 | 1 | 26 | 24 | 21 | 42 | 8 | k:Bacteria,p:Firmicutes,c:Clostridia,o:Clostridiales,f:Lachnospiraceae |
| OTU_817 | 0 | 0 | 0 | 1 | 0 | 1 | 0 | 0 | 3 | 0 | 1 | 0 | k:Bacteria,p:Firmicutes,c:Clostridia,o:Clostridiales,f:Lachnospiraceae |
| OTU_818 | 0 | 0 | 0 | 1 | 0 | 2 | 0 | 0 | 0 | 0 | 2 | 0 | k:Bacteria,p:Actinobacteria,c:Actinobacteria,o:Actinomycetales,f:Actinomycetaceae,g:Actinomyces |

Table S3. Predicted significantly KEGG pathway UUO rats versus sham rats

| Predicted KEGG pathway | Sham | | UUO | | P |
| --- | --- | --- | --- | --- | --- |
|  | Relative abundance | SD | Relative abundance | SD |  |
| K07148; uncharacterized protein | 3.19E-03 | 1.93E-03 | 1.24E-03 | 1.62E-04 | 3.36E-02 |
| K06020; sulfate-transporting ATPase [EC:3.6.3.25] | 5.69E-03 | 1.13E-03 | 2.08E-03 | 6.54E-04 | 5.01E-05 |
| K07506; AraC family transcriptional regulator | 2.80E-03 | 1.18E-03 | 1.63E-03 | 1.32E-04 | 3.65E-02 |
| K00038; 3alpha(or 20beta)-hydroxysteroid dehydrogenase [EC:1.1.1.53] | 9.11E-04 | 4.83E-04 | 4.28E-04 | 7.02E-05 | 3.53E-02 |
| K06034; sulfopyruvate decarboxylase subunit alpha [EC:4.1.1.79] | 2.84E-05 | 2.45E-05 | 4.16E-06 | 2.35E-06 | 3.68E-02 |
| K06076; long-chain fatty acid transport protein | 4.19E-03 | 2.21E-03 | 1.96E-03 | 3.81E-04 | 3.51E-02 |
| K07679; two-component system, NarL family, sensor histidine kinase EvgS [EC:2.7.13.3] | 3.71E-03 | 1.26E-03 | 2.46E-03 | 2.19E-04 | 3.74E-02 |
| K03559; biopolymer transport protein ExbD | 3.12E-03 | 2.11E-03 | 9.84E-04 | 2.74E-04 | 3.39E-02 |
| K14092; energy-converting hydrogenase A subunit A | 8.61E-06 | 8.05E-06 | 8.62E-07 | 7.50E-07 | 4.06E-02 |
| K14115; energy-converting hydrogenase B subunit F | 1.69E-05 | 1.66E-05 | 4.36E-07 | 1.55E-07 | 3.49E-02 |
| K06606; inosose isomerase [EC:5.3.99.-] | 6.39E-04 | 5.66E-04 | 7.70E-05 | 5.18E-05 | 3.58E-02 |
| K02063; thiamine transport system permease protein | 1.60E-03 | 1.16E-03 | 4.28E-04 | 1.51E-04 | 3.46E-02 |
| K01574; acetoacetate decarboxylase [EC:4.1.1.4] | 9.98E-04 | 6.04E-04 | 3.84E-04 | 1.67E-04 | 3.68E-02 |
| K03337; 5-deoxy-glucuronate isomerase [EC:5.3.1.-] | 6.09E-03 | 1.76E-03 | 4.29E-03 | 5.92E-04 | 3.91E-02 |
| K00111; glycerol-3-phosphate dehydrogenase [EC:1.1.5.3] | 6.28E-02 | 5.75E-03 | 5.49E-02 | 5.43E-03 | 3.36E-02 |
| K03567; glycine cleavage system transcriptional repressor | 2.71E-04 | 2.01E-04 | 6.62E-05 | 6.29E-05 | 3.89E-02 |
| K09145; hypothetical protein | 1.09E-03 | 7.11E-04 | 3.78E-04 | 1.82E-04 | 3.83E-02 |
| K00274; monoamine oxidase [EC:1.4.3.4] | 9.45E-03 | 2.32E-03 | 6.97E-03 | 9.14E-04 | 3.49E-02 |
| K02806; PTS system, nitrogen regulatory IIA component [EC:2.7.1.69] | 8.45E-03 | 1.76E-03 | 6.48E-03 | 8.70E-04 | 3.39E-02 |
| K11624; two-component system, NarL family, response regulator YdfI | 2.30E-04 | 1.34E-04 | 9.00E-05 | 3.81E-05 | 3.36E-02 |
| K01592; tyrosine decarboxylase [EC:4.1.1.25] | 8.72E-05 | 4.26E-05 | 4.40E-05 | 8.61E-06 | 3.49E-02 |
| K00011; aldehyde reductase [EC:1.1.1.21] | 1.77E-05 | 1.47E-05 | 3.09E-06 | 8.29E-07 | 3.53E-02 |
| K04026; ethanolamine utilization protein EutL | 3.79E-03 | 7.71E-04 | 2.82E-03 | 6.08E-04 | 3.72E-02 |
| K01425; glutaminase [EC:3.5.1.2] | 3.10E-02 | 6.21E-03 | 3.80E-02 | 3.66E-03 | 3.78E-02 |
| K01843; lysine 2,3-aminomutase [EC:5.4.3.2] | 1.86E-02 | 1.42E-03 | 2.13E-02 | 2.33E-03 | 3.49E-02 |
| K05831; LysW-gamma-L-lysine carboxypeptidase | 8.76E-06 | 6.42E-06 | 2.22E-06 | 1.29E-06 | 3.49E-02 |
| K02445; MFS transporter, OPA family, glycerol-3-phosphate transporter | 1.03E-02 | 3.53E-03 | 6.53E-03 | 1.29E-03 | 3.37E-02 |
| K02549; O-succinylbenzoate synthase [EC:4.2.1.113] | 5.07E-03 | 3.78E-03 | 1.40E-03 | 3.71E-04 | 3.94E-02 |
| K01664; para-aminobenzoate synthetase component II [EC:2.6.1.85] | 1.67E-03 | 9.36E-04 | 7.46E-04 | 1.69E-04 | 3.91E-02 |
| K03640; peptidoglycan-associated lipoprotein | 1.85E-03 | 1.20E-03 | 6.72E-04 | 2.09E-04 | 3.96E-02 |
| K00293; saccharopine dehydrogenase (NADP+, L-glutamate forming) [EC:1.5.1.10] | 5.44E-06 | 4.48E-06 | 1.02E-06 | 6.87E-07 | 3.77E-02 |
| K06374; spore maturation protein B | 1.25E-02 | 1.81E-03 | 1.52E-02 | 2.15E-03 | 3.98E-02 |
| K11623; two-component system, NarL family, sensor histidine kinase YdfH [EC:2.7.13.3] | 2.69E-04 | 1.90E-04 | 8.32E-05 | 3.49E-05 | 4.00E-02 |
| K01387; microbial collagenase [EC:3.4.24.3] | 5.76E-03 | 2.86E-03 | 2.88E-03 | 6.41E-04 | 3.70E-02 |
| K04749; anti-sigma B factor antagonist | 3.65E-03 | 6.63E-04 | 2.93E-03 | 3.11E-04 | 3.70E-02 |
| K00588; caffeoyl-CoA O-methyltransferase [EC:2.1.1.104] | 6.49E-04 | 1.92E-04 | 4.54E-04 | 4.52E-05 | 3.61E-02 |
| K03409; chemotaxis protein CheX | 1.09E-02 | 2.74E-03 | 1.40E-02 | 1.58E-03 | 3.99E-02 |
| K02109; F-type H+-transporting ATPase subunit b [EC:3.6.3.14] | 3.60E-02 | 9.22E-04 | 3.35E-02 | 2.38E-03 | 3.98E-02 |
| K11708; manganese/zinc/iron transport system permease protein | 1.23E-03 | 8.75E-04 | 3.50E-04 | 1.58E-04 | 3.54E-02 |
| K07587; O-phosphoseryl-tRNA synthetase [EC:6.1.1.27] | 6.77E-06 | 6.53E-06 | 3.99E-07 | 1.70E-07 | 3.77E-02 |
| K02283; pilus assembly protein CpaF | 8.62E-02 | 1.16E-02 | 1.02E-01 | 1.09E-02 | 3.77E-02 |
| K09835; prolycopene isomerase [EC:5.2.1.13] | 3.39E-04 | 1.29E-04 | 1.88E-04 | 8.71E-05 | 3.86E-02 |
| K07572; putative nucleotide binding protein | 1.68E-04 | 3.79E-05 | 2.13E-04 | 2.61E-05 | 3.65E-02 |
| K07453; putative restriction endonuclease | 1.17E-04 | 7.83E-05 | 3.77E-05 | 1.33E-05 | 3.46E-02 |
| K05297; rubredoxin-NAD+ reductase [EC:1.18.1.1] | 3.58E-03 | 3.31E-04 | 4.23E-03 | 5.66E-04 | 3.59E-02 |
| K06405; stage V sporulation protein AC | 1.37E-02 | 1.50E-03 | 1.58E-02 | 1.57E-03 | 3.49E-02 |
| K00615; transketolase [EC:2.2.1.1] | 1.80E-01 | 1.30E-02 | 1.63E-01 | 1.11E-02 | 3.45E-02 |
| K02124; V-type H+-transporting ATPase subunit K [EC:3.6.3.14] | 1.09E-02 | 1.08E-03 | 1.31E-02 | 1.93E-03 | 3.64E-02 |

Table S4. Identified plasma metabolites, fold Changes (FC), and p Values from UUO vs. sham rats

| No. | Rt_m/z | FC | p (one-way ANOVA) | P (Mann−Whitney U test) | FDR | Metabolites | Class |
| --- | --- | --- | --- | --- | --- | --- | --- |
| 1 | 4.63_790.5742m/z | 0.03 | 2.01E-06 | 3.70E-03 | 4.40E-04 | PE(40:7) | Glycerophospholipids |
| 2 | 4.64_381.3104m/z | 24.98 | 5.93E-06 | 3.95E-03 | 6.49E-04 | MG(20:3) | Glycerolipids |
| 3 | 1.74_431.2452m/z | 6.58 | 7.64E-06 | 3.95E-03 | 5.58E-04 | Chenodeoxycholic acid | Steroids |
| 4 | 2.08_221.1523m/z | 7.70 | 9.02E-06 | 3.95E-03 | 4.94E-04 | Acetylcarnitine | Acyl carnitines |
| 5 | 1.35_701.4934m/z | 0.16 | 2.87E-05 | 3.95E-03 | 1.26E-03 | PE(32:4) | Glycerophospholipids |
| 6 | 1.21_737.4198m/z | 0.30 | 4.23E-05 | 3.95E-03 | 1.55E-03 | PA(36:3) | Glycerophospholipids |
| 7 | 1.18_374.2045m/z | 0.32 | 6.32E-05 | 3.95E-03 | 1.98E-03 | Tetracosahexaenoic acid | Fatty acyls |
| 8 | 1.34_340.7595m/z | 0.34 | 6.90E-05 | 3.95E-03 | 1.89E-03 | Phytosphingosine | Amines |
| 9 | 1.31_260.1852m/z | 0.23 | 8.04E-05 | 3.95E-03 | 1.96E-03 | Hexanoylcarnitine | Acyl carnitines |
| 10 | 2.08_203.1419m/z | 20.58 | 8.24E-05 | 3.35E-03 | 1.80E-03 | Asymmetric dimethylarginine | Other |
| 11 | 2.08_215.1785m/z | 7.92 | 1.57E-04 | 3.88E-03 | 3.12E-03 | Oxoamide | Other |
| 12 | 0.62_127.0318m/z | 0.17 | 1.60E-04 | 3.88E-03 | 2.93E-03 | Butyrate | Organic acids |
| 13 | 2.08_175.1471m/z | 6.00 | 1.81E-04 | 3.95E-03 | 3.06E-03 | Phenol sulphate | Organic acids |
| 14 | 2.26_424.3420m/z | 2.33 | 1.97E-04 | 3.95E-03 | 3.08E-03 | Linoelaidyl carnitine | Acyl carnitines |
| 15 | 1.19_834.4734m/z | 0.39 | 2.57E-04 | 3.95E-03 | 3.75E-03 | PS(37:5) | Glycerophospholipids |
| 16 | 2.08_275.2001m/z | 5.11 | 2.86E-04 | 3.95E-03 | 3.91E-03 | MG(12:0) | Glycerolipids |
| 17 | 4.28_784.5815m/z | 0.11 | 3.62E-04 | 3.95E-03 | 4.66E-03 | PC(36:3) | Glycerophospholipids |
| 18 | 1.16_572.3439m/z | 0.50 | 3.88E-04 | 3.95E-03 | 4.72E-03 | LPC(22:4) | Glycerophospholipids |
| 19 | 1.41_169.0754m/z | 0.05 | 4.36E-04 | 2.80E-03 | 5.03E-03 | Lysine | Amino acids |
| 20 | 1.29_491.2976m/z | 0.10 | 4.77E-04 | 5.59E-03 | 5.22E-03 | LPE(18:4) | Glycerophospholipids |
| 21 | 3.40_814.5133m/z | 5.11 | 4.89E-04 | 3.95E-03 | 5.10E-03 | PS(35:1) | Glycerophospholipids |
| 22 | 2.25_448.3418m/z | 4.00 | 4.89E-04 | 3.95E-03 | 4.87E-03 | Oleoylcarnitine | Acyl carnitines |
| 23 | 2.08_247.1681m/z | 11.93 | 5.09E-04 | 3.88E-03 | 4.85E-03 | Butenylcarnitine | Acyl carnitines |
| 24 | 4.64_876.8005m/z | 19.13 | 5.25E-04 | 3.88E-03 | 4.79E-03 | TG(52:2) | Glycerolipids |
| 25 | 2.48_426.5496m/z | 18.33 | 7.08E-04 | 3.88E-03 | 6.20E-03 | Allocholic acid | Steroids |
| 26 | 1.97_396.3099m/z | 2.18 | 1.04E-03 | 5.00E-03 | 8.77E-03 | 3-hydroxytridecanoyl carnitine | Acyl carnitines |
| 27 | 1.88_526.3418m/z | 0.58 | 1.20E-03 | 3.95E-03 | 9.71E-03 | LPE(22:6) | Glycerophospholipids |
| 28 | 1.27_566.3287m/z | 0.04 | 1.22E-03 | 3.35E-03 | 9.50E-03 | LPE(24:1) | Glycerophospholipids |
| 29 | 0.72_186.0866m/z | 2.90 | 1.33E-03 | 3.95E-03 | 1.00E-02 | Uric acid | Organic acids |
| 30 | 2.08_289.2149m/z | 8.99 | 1.41E-03 | 3.88E-03 | 1.03E-02 | MG(13:0) | Glycerolipids |
| 31 | 1.85_746.4723m/z | 0.32 | 1.48E-03 | 3.95E-03 | 1.04E-02 | PC(32:5) | Glycerophospholipids |
| 32 | 1.75_159.1158m/z | 5.50 | 1.48E-03 | 3.95E-03 | 1.02E-02 | phenylacetic acid | Other |
| 33 | 3.20_466.3261m/z | 3.81 | 1.54E-03 | 3.95E-03 | 1.02E-02 | Glycocholic acid | Steroids |
| 34 | 1.99_568.3596m/z | 0.56 | 1.60E-03 | 3.95E-03 | 1.03E-02 | LPC(22:6) | Glycerophospholipids |
| 35 | 2.48_426.3577m/z | 2.12 | 1.75E-03 | 3.95E-03 | 1.10E-02 | Ursocholic acid | Steroids |
| 36 | 2.96_240.0986m/z | 1.87 | 1.99E-03 | 2.16E-03 | 1.21E-02 | Cysteine S-sulfate | Organic acids |
| 37 | 2.57_414.3567m/z | 5.85 | 2.15E-03 | 1.63E-02 | 1.27E-02 | Heptadecanoyl carnitine | Acyl carnitines |
| 38 | 0.70_134.0318m/z | 0.52 | 2.27E-03 | 8.13E-03 | 1.31E-02 | Cytosine | Amino acids |
| 39 | 2.21_386.3254m/z | 7.86 | 2.27E-03 | 1.04E-02 | 1.28E-02 | Pentadecanoylcarnitine | Acyl carnitines |
| 40 | 1.83_399.2363m/z | 19.46 | 2.31E-03 | 2.80E-03 | 1.26E-02 | MG(20:5) | Glycerolipids |
| 41 | 1.99_546.3476m/z | 0.47 | 2.50E-03 | 3.95E-03 | 1.34E-02 | LPC(20:3) | Glycerophospholipids |
| 42 | 4.46_413.3218m/z | 6.38 | 2.53E-03 | 6.12E-03 | 1.32E-02 | MG(22:1) | Glycerolipids |
| 43 | 2.85_732.5535m/z | 0.06 | 2.55E-03 | 5.59E-03 | 1.30E-02 | PC(32:1) | Glycerophospholipids |
| 44 | 1.86_660.4188m/z | 0.29 | 2.69E-03 | 3.95E-03 | 1.34E-02 | PE(30:2) | Glycerophospholipids |
| 45 | 2.04_508.3570m/z | 0.06 | 2.74E-03 | 1.04E-02 | 1.33E-02 | LPE(20:1) | Glycerophospholipids |
| 46 | 5.64_506.3602m/z | 0.22 | 2.76E-03 | 2.50E-02 | 1.31E-02 | LPE(20:2) | Glycerophospholipids |
| 47 | 4.28_792.5892m/z | 0.23 | 2.90E-03 | 6.49E-03 | 1.35E-02 | PE(40:6) | Glycerophospholipids |
| 48 | 1.79_494.3416m/z | 0.21 | 2.94E-03 | 1.04E-02 | 1.34E-02 | LPC(16:1) | Glycerophospholipids |
| 49 | 5.62_162.1255m/z | 0.54 | 2.96E-03 | 5.00E-03 | 1.32E-02 | Tryptophanol | Other |
| 50 | 1.31_199.0204m/z | 0.08 | 3.01E-03 | 3.70E-03 | 1.32E-02 | Tyrosine | Amino acids |
| 51 | 0.74_280.1383m/z | 2.17 | 3.04E-03 | 1.04E-02 | 1.31E-02 | Hexenoylcarnitine | Acyl carnitines |
| 52 | 0.59_176.1019m/z | 2.07 | 3.06E-03 | 1.03E-02 | 1.29E-02 | Allantoin | Other |
| 53 | 2.12_598.3977m/z | 0.40 | 3.28E-03 | 1.04E-02 | 1.36E-02 | LPC(22:2) | Glycerophospholipids |
| 54 | 2.04_209.1319m/z | 7.88 | 3.32E-03 | 3.88E-03 | 1.35E-02 | Kynurenine | Amino acids |
| 55 | 5.64_504.3442m/z | 0.09 | 3.33E-03 | 1.63E-02 | 1.33E-02 | LPE(20:3) | Glycerophospholipids |
| 56 | 1.78_207.1369m/z | 46.15 | 3.36E-03 | 2.80E-03 | 1.31E-02 | Dimethylglycine | Amino acids |
| 57 | 1.78_538.3682m/z | 0.34 | 3.42E-03 | 3.74E-02 | 1.31E-02 | LPE(22:0) | Glycerophospholipids |
| 58 | 1.90_835.5591m/z | 2.99 | 3.56E-03 | 3.95E-03 | 1.34E-02 | PI(34:2) | Glycerophospholipids |
| 59 | 2.04_287.1997m/z | 9.10 | 3.62E-03 | 3.70E-03 | 1.34E-02 | Hexadecanedioic acid | Fatty acyls |
| 60 | 2.65_385.2724m/z | 17.42 | 3.78E-03 | 3.70E-03 | 1.38E-02 | S-Adenosylhomocysteine | Other |
| 61 | 2.82_478.3284m/z | 0.17 | 4.18E-03 | 1.04E-02 | 1.50E-02 | LPE(15:0) | Glycerophospholipids |
| 62 | 2.05_239.1789m/z | 7.74 | 4.21E-03 | 6.49E-03 | 1.49E-02 | Canavalmine | Amines |
| 63 | 1.84_768.4847m/z | 0.22 | 4.41E-03 | 6.39E-03 | 1.53E-02 | PS(35:5) | Glycerophospholipids |
| 64 | 0.59_285.1079m/z | 2.10 | 4.42E-03 | 1.04E-02 | 1.51E-02 | p-Cresyl glucuronide | Other |
| 65 | 1.88_548.3547m/z | 0.60 | 4.53E-03 | 3.95E-03 | 1.53E-02 | LPC(20:2) | Glycerophospholipids |
| 66 | 2.08_375.2878m/z | 32.31 | 4.59E-03 | 3.35E-03 | 1.52E-02 | MG(18:3) | Glycerolipids |
| 67 | 2.18_429.2297m/z | 8.46 | 4.81E-03 | 9.75E-03 | 1.57E-02 | Nutriacholic acid | Steroids |
| 68 | 2.04_213.1631m/z | 4.11 | 5.02E-03 | 1.61E-02 | 1.62E-02 | 3-Hydroxyhippuric acid | Organic acids |
| 69 | 4.40_284.2932m/z | 3.51 | 5.07E-03 | 9.75E-03 | 1.61E-02 | Octadecanamide | Fatty acyls |
| 70 | 2.04_293.2250m/z | 13.98 | 5.07E-03 | 9.11E-03 | 1.59E-02 | Heptadecanoic acid | Fatty acyls |
| 71 | 1.84_787.5200m/z | 0.28 | 5.08E-03 | 1.04E-02 | 1.57E-02 | PS(35:4) | Glycerophospholipids |
| 72 | 1.70_641.4258m/z | 0.35 | 5.21E-03 | 2.50E-02 | 1.58E-02 | DG(35:4) | Glycerolipids |
| 73 | 1.90_482.3142m/z | 0.45 | 5.38E-03 | 1.63E-02 | 1.61E-02 | LPC(15:0) | Glycerophospholipids |
| 74 | 2.05_313.2154m/z | 4.27 | 5.40E-03 | 1.03E-02 | 1.60E-02 | Androsterone | Steroids |
| 75 | 2.00_552.3831m/z | 0.39 | 5.42E-03 | 5.47E-02 | 1.58E-02 | LPC(20:0) | Glycerophospholipids |
| 76 | 1.76_714.4729m/z | 0.31 | 5.59E-03 | 1.63E-02 | 1.61E-02 | PE(31:1) | Glycerophospholipids |
| 77 | 0.79_126.0653m/z | 0.39 | 5.70E-03 | 1.03E-02 | 1.62E-02 | Taurine | Amino acids |
| 78 | 3.67_441.2394m/z | 3.15 | 5.72E-03 | 6.49E-03 | 1.61E-02 | MG(22:6) | Glycerolipids |
| 79 | 1.85_407.2776m/z | 42.72 | 5.74E-03 | 3.70E-03 | 1.59E-02 | 3-Oxocholic acid | Steroids |
| 80 | 1.78_480.2777m/z | 0.66 | 5.87E-03 | 3.95E-03 | 1.61E-02 | LPE(18:1) | Glycerophospholipids |
| 81 | 5.66_156.8888m/z | 0.79 | 5.95E-03 | 1.04E-02 | 1.61E-02 | Aspartic acid | Amino acids |
| 82 | 2.05_183.1160m/z | 11.87 | 6.04E-03 | 6.12E-03 | 1.61E-02 | Homovanillate | Organic acids |
| 83 | 2.95_522.6229m/z | 5.20 | 6.05E-03 | 1.03E-02 | 1.60E-02 | Tauroursodeoxycholic acid | Steroids |
| 84 | 1.49_845.4360m/z | 0.11 | 6.06E-03 | 2.47E-02 | 1.58E-02 | PI(32:2) | Glycerophospholipids |
| 85 | 2.05_141.0693m/z | 13.84 | 6.09E-03 | 3.88E-03 | 1.57E-02 | Succinate | Organic acids |
| 86 | 2.85_760.5835m/z | 0.14 | 6.23E-03 | 1.04E-02 | 1.59E-02 | PC(34:1) | Glycerophospholipids |
| 87 | 0.94_214.1063m/z | 4.76 | 6.63E-03 | 1.03E-02 | 1.67E-02 | Indoxyl sulfate | Organic acids |
| 88 | 2.05_779.5237m/z | 0.32 | 6.75E-03 | 2.50E-02 | 1.68E-02 | PE(38:7) | Glycerophospholipids |
| 89 | 2.05_181.1001m/z | 48.23 | 6.77E-03 | 3.35E-03 | 1.67E-02 | Oxalic acid | Organic acids |
| 90 | 2.60_184.0730m/z | 1.49 | 6.89E-03 | 8.66E-03 | 1.68E-02 | spermidine | Amines |
| 91 | 1.84_790.4979m/z | 0.34 | 6.95E-03 | 6.49E-03 | 1.67E-02 | PC(34:5) | Glycerophospholipids |
| 92 | 1.54_708.4735m/z | 0.34 | 6.99E-03 | 1.63E-02 | 1.66E-02 | PS(30:0) | Glycerophospholipids |
| 93 | 3.92_353.2323m/z | 2.00 | 7.23E-03 | 6.49E-03 | 1.70E-02 | Progesterone | Steroids |
| 94 | 2.04_225.1630m/z | 6.39 | 7.31E-03 | 1.61E-02 | 1.70E-02 | 5-Hydroxykynurenine | Amino acids |
| 95 | 2.04_152.0613m/z | 23.53 | 7.33E-03 | 3.88E-03 | 1.69E-02 | Creatinine | Organic acids |
| 96 | 1.67_274.2740m/z | 3.20 | 7.50E-03 | 3.95E-03 | 1.71E-02 | Palmitic acid | Fatty acyls |
| 97 | 2.04_165.0693m/z | 15.06 | 7.82E-03 | 1.56E-02 | 1.77E-02 | Glutamate | Amino acids |
| 98 | 3.99_813.6836m/z | 7.46 | 7.88E-03 | 1.45E-02 | 1.76E-02 | TG(49:4) | Glycerolipids |
| 99 | 5.63_188.1407m/z | 0.68 | 8.09E-03 | 1.04E-02 | 1.79E-02 | Tryptophan | Amino acids |
| 100 | 2.04_823.5492m/z | 0.23 | 8.40E-03 | 1.04E-02 | 1.84E-02 | PG(40:6) | Glycerophospholipids |
| 101 | 2.86_282.2780m/z | 4.12 | 8.49E-03 | 1.04E-02 | 1.84E-02 | Fumarycarnitine | Acyl carnitines |
| 102 | 3.44_300.9912m/z | 12.53 | 8.60E-03 | 4.76E-03 | 1.85E-02 | Homovanillic acid sulfate | Organic acids |
| 103 | 2.05_155.0844m/z | 12.74 | 8.73E-03 | 3.88E-03 | 1.86E-02 | Glutarate | Organic acids |
| 104 | 1.12_372.2378m/z | 4.37 | 9.00E-03 | 5.47E-02 | 1.90E-02 | Aspidospermine | Amines |
| 105 | 1.84_227.1417m/z | 174.79 | 9.15E-03 | 3.35E-03 | 1.91E-02 | p-Cresyl sulfate | Organic acids |
| 106 | 2.66_460.2818m/z | 2.30 | 9.25E-03 | 2.50E-02 | 1.91E-02 | Linolenyl carnitine | Acyl carnitines |
| 107 | 1.85_426.2512m/z | 54.89 | 9.32E-03 | 1.32E-02 | 1.91E-02 | Cholate | Steroids |
| 108 | 1.84_253.1579m/z | 24.92 | 9.45E-03 | 3.95E-03 | 1.92E-02 | Dodecanedioic acid | Fatty acyls |
| 109 | 1.84_317.2260m/z | 25.26 | 9.54E-03 | 3.95E-03 | 1.92E-02 | Linolenic acid | Fatty acyls |
| 110 | 1.84_239.1782m/z | 488.94 | 9.57E-03 | 2.75E-03 | 1.91E-02 | Decyl acetate | Fatty acyls |
| 111 | 1.21_381.2316m/z | 0.56 | 9.63E-03 | 2.50E-02 | 1.90E-02 | Tetracosapentaenoic acid (24:5n-6) | Fatty acyls |
| 112 | 2.75_428.3733m/z | 2.46 | 9.76E-03 | 1.63E-02 | 1.91E-02 | Stearoylcarnitine | Acyl carnitines |
| 113 | 2.05_836.5548m/z | 7.38 | 9.94E-03 | 6.12E-03 | 1.93E-02 | PS(40:6) | Glycerophospholipids |
| 114 | 1.75_309.2563m/z | 4.70 | 1.01E-02 | 3.95E-03 | 1.94E-02 | Eicosadienoic acid | Fatty acyls |
| 115 | 4.14_783.5756m/z | 0.07 | 1.05E-02 | 1.63E-02 | 2.00E-02 | PE(38:5) | Glycerophospholipids |
| 116 | 3.14_856.5820m/z | 0.13 | 1.06E-02 | 1.61E-02 | 2.00E-02 | PC(40:6) | Glycerophospholipids |
| 117 | 2.17_539.3618m/z | 0.59 | 1.08E-02 | 1.63E-02 | 2.01E-02 | LPC(18:1) | Glycerophospholipids |
| 118 | 3.72_468.3427m/z | 1.51 | 1.15E-02 | 1.04E-02 | 2.14E-02 | Coprocholic acid | Steroids |
| 119 | 1.76_758.4988m/z | 0.26 | 1.18E-02 | 3.74E-02 | 2.17E-02 | PS(34:3) | Glycerophospholipids |
| 120 | 1.21_530.3315m/z | 0.47 | 1.22E-02 | 2.50E-02 | 2.22E-02 | LPE(22:4) | Glycerophospholipids |
| 121 | 2.18_319.2413m/z | 23.61 | 1.23E-02 | 3.88E-03 | 2.23E-02 | Leukotriene A4 | Fatty acyls |
| 122 | 2.19_518.3430m/z | 0.66 | 1.24E-02 | 1.63E-02 | 2.22E-02 | LPC(18:3) | Glycerophospholipids |
| 123 | 2.08_822.5776m/z | 0.45 | 1.27E-02 | 3.74E-02 | 2.26E-02 | PE(42:5) | Glycerophospholipids |
| 124 | 1.47_130.1584m/z | 0.71 | 1.27E-02 | 2.50E-02 | 2.24E-02 | Uracil | Other |
| 125 | 1.85_243.1369m/z | 57.36 | 1.30E-02 | 3.88E-03 | 2.28E-02 | Thymidine | Amines |
| 126 | 0.74_85.0278m/z | 0.67 | 1.33E-02 | 2.50E-02 | 2.30E-02 | Imidazolone | Organic acids |
| 127 | 2.10_778.5510m/z | 0.49 | 1.34E-02 | 2.50E-02 | 2.32E-02 | PC(36:6) | Glycerophospholipids |
| 128 | 3.05_522.4888m/z | 2.52 | 1.36E-02 | 2.50E-02 | 2.32E-02 | Taurochenodesoxycholic acid | Steroids |
| 129 | 2.00_524.3330m/z | 0.46 | 1.36E-02 | 3.95E-03 | 2.31E-02 | LPC(18:0) | Glycerophospholipids |
| 130 | 2.37_878.6038m/z | 3.12 | 1.38E-02 | 1.04E-02 | 2.32E-02 | PC(44:12) | Glycerophospholipids |
| 131 | 2.05_223.1476m/z | 31.35 | 1.42E-02 | 2.01E-02 | 2.37E-02 | 3-Indolehydracrylic acid | Organic acids |
| 132 | 3.14_828.5513m/z | 0.03 | 1.46E-02 | 8.70E-02 | 2.43E-02 | PC(38:6) | Glycerophospholipids |
| 133 | 2.41_516.3057m/z | 0.49 | 1.47E-02 | 1.04E-02 | 2.42E-02 | LPC(18:4) | Glycerophospholipids |
| 134 | 0.63_144.0647m/z | 0.39 | 1.53E-02 | 1.04E-02 | 2.50E-02 | Serine | Amino acids |
| 135 | 3.05_959.6446m/z | 2.40 | 1.54E-02 | 6.49E-03 | 2.49E-02 | TG(58:13) | Glycerolipids |
| 136 | 2.65_804.5567m/z | 0.28 | 1.60E-02 | 3.74E-02 | 2.58E-02 | PC(38:7) | Glycerophospholipids |
| 137 | 3.48_836.6117m/z | 0.22 | 1.62E-02 | 3.74E-02 | 2.59E-02 | PC(38:2) | Glycerophospholipids |
| 138 | 4.64_314.1046m/z | 12.92 | 1.64E-02 | 4.76E-03 | 2.61E-02 | Decenoylcarnitine | Acyl carnitines |
| 139 | 1.19_794.4806m/z | 0.60 | 1.69E-02 | 3.74E-02 | 2.66E-02 | PC(34:2) | Glycerophospholipids |
| 140 | 0.96_271.1396m/z | 23.71 | 1.73E-02 | 4.95E-02 | 2.71E-02 | Phenyl glucuronide | Other |
| 141 | 3.43_305.2463m/z | 2.81 | 1.74E-02 | 5.47E-02 | 2.70E-02 | Arachidonic acid | Fatty acyls |
| 142 | 0.69_231.1698m/z | 1.84 | 1.83E-02 | 6.49E-02 | 2.83E-02 | Isocitrate | Organic acids |
| 143 | 2.30_321.2568m/z | 13.41 | 1.86E-02 | 3.95E-03 | 2.86E-02 | 3-Oxooctadecanoic acid | Fatty acyls |
| 144 | 2.04_843.5366m/z | 0.13 | 1.99E-02 | 6.51E-02 | 3.02E-02 | PG(42:10) | Glycerophospholipids |
| 145 | 1.94_753.4799m/z | 0.75 | 2.01E-02 | 2.50E-02 | 3.04E-02 | PE(36:6) | Glycerophospholipids |
| 146 | 3.35_423.3250m/z | 8.15 | 2.02E-02 | 8.70E-02 | 3.02E-02 | 7-Ketocholesterol | Steroids |
| 147 | 1.85_285.1843m/z | 142.31 | 2.02E-02 | 2.16E-03 | 3.01E-02 | Stearic acid | Fatty acyls |
| 148 | 3.48_691.4618m/z | 17.96 | 2.05E-02 | 6.12E-03 | 3.03E-02 | DG(38:7) | Glycerolipids |
| 149 | 2.91_496.6016m/z | 0.64 | 2.06E-02 | 3.74E-02 | 3.02E-02 | LPC(16:0) | Glycerophospholipids |
| 150 | 1.84_225.1629m/z | 313.22 | 2.06E-02 | 2.16E-03 | 3.01E-02 | 3-Hydroxykynurenine | Amino acids |
| 151 | 3.06_811.4887m/z | 2.75 | 2.08E-02 | 1.63E-02 | 3.01E-02 | PG(36:3) | Glycerophospholipids |
| 152 | 1.54_664.4467m/z | 0.35 | 2.08E-02 | 3.74E-02 | 3.00E-02 | PE(30:0) | Glycerophospholipids |
| 153 | 3.86_211.1215m/z | 2.71 | 2.08E-02 | 5.47E-02 | 2.98E-02 | Phenaceturate | Organic acids |
| 154 | 1.78_344.2788m/z | 1.36 | 2.10E-02 | 3.74E-02 | 2.99E-02 | Sucrose | Other |
| 155 | 4.13_832.5830m/z | 0.36 | 2.15E-02 | 2.50E-02 | 3.04E-02 | PC(40:7) | Glycerophospholipids |
| 156 | 2.59_221.1525m/z | 145.23 | 2.16E-02 | 2.16E-03 | 3.04E-02 | 5-Hydroxytryptophan | Amino acids |
| 157 | 2.59_184.0723m/z | 1.58 | 2.19E-02 | 2.50E-02 | 3.05E-02 | γ-Guanidinobutyrate | Organic acids |
| 158 | 4.14_782.5661m/z | 0.06 | 2.20E-02 | 2.00E-01 | 3.05E-02 | PC(36:4) | Glycerophospholipids |
| 159 | 3.35_735.4877m/z | 16.37 | 2.22E-02 | 1.04E-02 | 3.05E-02 | DG(42:6) | Glycerolipids |
| 160 | 1.67_276.2795m/z | 6.18 | 2.22E-02 | 3.95E-03 | 3.04E-02 | 3-hydroxypentadecanoic acid | Fatty acyls |
| 161 | 2.28_544.3228m/z | 0.57 | 2.25E-02 | 3.74E-02 | 3.06E-02 | LPC(20:4) | Glycerophospholipids |
| 162 | 3.93_279.2315m/z | 1.50 | 2.29E-02 | 3.74E-02 | 3.09E-02 | Hydroxyisovalerylcarnitine | Acyl carnitines |
| 163 | 2.04_801.5347m/z | 0.30 | 2.35E-02 | 5.47E-02 | 3.16E-02 | PS(36:4) | Glycerophospholipids |
| 164 | 2.15_353.2453m/z | 20.42 | 2.37E-02 | 3.95E-03 | 3.16E-02 | MG(16:0) | Glycerolipids |
| 165 | 2.04_277.1957m/z | 12.94 | 2.43E-02 | 1.52E-02 | 3.22E-02 | Urocanate | Organic acids |
| 166 | 3.97_50.0931m/z | 0.01 | 2.44E-02 | 1.35E-01 | 3.21E-02 | Methanol | Other |
| 167 | 1.97_634.4003m/z | 0.64 | 2.46E-02 | 7.82E-02 | 3.23E-02 | PE(28:1) | Glycerophospholipids |
| 168 | 1.11_728.4692m/z | 0.45 | 2.51E-02 | 2.50E-02 | 3.28E-02 | PE(35:5) | Glycerophospholipids |
| 169 | 1.96_662.4488m/z | 0.62 | 2.55E-02 | 5.47E-02 | 3.30E-02 | PE(30:1) | Glycerophospholipids |
| 170 | 2.30_339.2676m/z | 5.14 | 2.55E-02 | 3.95E-03 | 3.29E-02 | MG(15:0) | Glycerolipids |
| 171 | 2.66_215.1790m/z | 3.69 | 2.58E-02 | 8.66E-03 | 3.31E-02 | Citrate | Organic acids |
| 172 | 5.64_502.3284m/z | 0.19 | 2.65E-02 | 3.74E-02 | 3.37E-02 | LPE(20:4) | Glycerophospholipids |
| 173 | 1.37_704.3771m/z | 0.12 | 2.65E-02 | 1.03E-02 | 3.36E-02 | PE(32:5) | Glycerophospholipids |
| 174 | 3.15_183.1157m/z | 3.24 | 2.74E-02 | 2.00E-01 | 3.45E-02 | Sorbitol | Other |
| 175 | 1.76_706.4622m/z | 0.38 | 2.77E-02 | 5.42E-02 | 3.47E-02 | PS(30:1) | Glycerophospholipids |
| 176 | 1.22_808.4961m/z | 0.62 | 2.87E-02 | 3.74E-02 | 3.57E-02 | PS(38:6) | Glycerophospholipids |
| 177 | 1.79_197.6096m/z | 6.64 | 2.89E-02 | 6.49E-02 | 3.58E-02 | 5-Hydroxytryptamine | Amines |
| 178 | 3.11_739.5072m/z | 8.62 | 2.97E-02 | 3.95E-03 | 3.65E-02 | DG(44:10) | Glycerolipids |
| 179 | 1.69_362.3264m/z | 3.99 | 3.00E-02 | 3.95E-03 | 3.67E-02 | MG(17:0) | Glycerolipids |
| 180 | 3.35_459.3464m/z | 5.62 | 3.04E-02 | 8.74E-02 | 3.70E-02 | 3-Sulfodeoxycholic acid | Steroids |
| 181 | 0.57_136.0463m/z | 0.62 | 3.19E-02 | 3.74E-02 | 3.85E-02 | 2-Hydroxyvalerate | Organic acids |
| 182 | 1.79_510.3188m/z | 0.41 | 3.19E-02 | 7.82E-02 | 3.84E-02 | LPC(17:0) | Glycerophospholipids |
| 183 | 4.05_780.5501m/z | 0.21 | 3.19E-02 | 2.47E-02 | 3.82E-02 | PC(36:5) | Glycerophospholipids |
| 184 | 2.70_832.4656m/z | 2.22 | 3.30E-02 | 2.50E-02 | 3.93E-02 | PS(40:8) | Glycerophospholipids |
| 185 | 1.68_320.3058m/z | 5.33 | 3.30E-02 | 6.49E-03 | 3.91E-02 | MG(14:0) | Glycerolipids |
| 186 | 2.58_431.2076m/z | 158.16 | 3.47E-02 | 2.80E-03 | 4.08E-02 | Ursodeoxycholic acid | Steroids |
| 187 | 0.72_216.0975m/z | 1.84 | 3.47E-02 | 5.47E-02 | 4.06E-02 | Phenylacetylglutamine | Organic acids |
| 188 | 1.17_184.0751m/z | 1.60 | 3.63E-02 | 2.50E-02 | 4.23E-02 | Carnitine | Other |
| 189 | 0.56_139.0571m/z | 2.24 | 3.66E-02 | 2.45E-02 | 4.24E-02 | 8-Hydroxypurine | Other |
| 190 | 0.73_334.1126m/z | 8.14 | 3.69E-02 | 3.95E-03 | 4.25E-02 | N2,N2-Dimethylguanosine | Other |
| 191 | 3.40_124.9994m/z | 6.63 | 3.69E-02 | 4.59E-02 | 4.23E-02 | 2-Ketobutyric acid | Organic acids |
| 192 | 2.85_743.4861m/z | 5.09 | 3.69E-02 | 1.63E-02 | 4.21E-02 | PG(34:4) | Glycerophospholipids |
| 193 | 3.00_431.3154m/z | 13.37 | 3.72E-02 | 2.50E-02 | 4.22E-02 | Deoxycholic acid | Steroids |
| 194 | 0.56_170.0317m/z | 0.54 | 3.76E-02 | 5.47E-02 | 4.24E-02 | Creatine | Organic acids |
| 195 | 2.18_495.3375m/z | 0.42 | 3.78E-02 | 3.74E-02 | 4.25E-02 | LPE(18:2) | Glycerophospholipids |
| 196 | 1.10_174.0541m/z | 3.53 | 3.84E-02 | 6.51E-02 | 4.29E-02 | Adenine | Other |
| 197 | 1.10_262.1068m/z | 1.81 | 3.84E-02 | 5.42E-02 | 4.27E-02 | Pseudouridine | Other |
| 198 | 4.40_810.6013m/z | 0.20 | 3.86E-02 | 2.47E-02 | 4.27E-02 | PC(38:4) | Glycerophospholipids |
| 199 | 1.88_493.3372m/z | 0.60 | 3.88E-02 | 3.74E-02 | 4.27E-02 | LPE(18:3) | Glycerophospholipids |
| 200 | 4.35_270.2788m/z | 1.37 | 3.88E-02 | 5.47E-02 | 4.25E-02 | Octadecylamine | Amines |
| 201 | 1.95_722.4536m/z | 0.36 | 4.02E-02 | 3.74E-02 | 4.37E-02 | PS(31:0) | Glycerophospholipids |
| 202 | 0.89_198.1114m/z | 1.91 | 4.06E-02 | 5.47E-02 | 4.40E-02 | Guanidinosuccinate | Organic acids |
| 203 | 0.73_191.0441m/z | 1.76 | 4.11E-02 | 1.09E-01 | 4.43E-02 | Oxalosuccinic acid | Organic acids |
| 204 | 2.36_488.2302m/z | 2.06 | 4.15E-02 | 3.70E-02 | 4.46E-02 | Glycoursodeoxycholic acid | Steroids |
| 205 | 3.09_327.2310m/z | 3.27 | 4.21E-02 | 1.09E-01 | 4.50E-02 | Atherosperminine | Amines |
| 206 | 2.28_371.2556m/z | 59.85 | 4.22E-02 | 1.52E-02 | 4.49E-02 | Adrenic acid | Fatty acyls |
| 207 | 1.67_361.2003m/z | 1.98 | 4.23E-02 | 1.09E-01 | 4.48E-02 | Aldosterone | Steroids |
| 208 | 3.47_262.2520m/z | 17.24 | 4.26E-02 | 8.74E-02 | 4.49E-02 | Methylmalonylcarnitine | Acyl carnitines |
| 209 | 1.94_733.4675m/z | 0.57 | 4.40E-02 | 7.82E-02 | 4.61E-02 | PE(34:4) | Glycerophospholipids |
| 210 | 2.60_464.3127m/z | 1.49 | 4.47E-02 | 1.09E-01 | 4.66E-02 | Octadecenoylcarnitine | Acyl carnitines |
| 211 | 1.69_817.5818m/z | 62.80 | 4.52E-02 | 4.63E-02 | 4.69E-02 | PC(38:9) | Glycerophospholipids |
| 212 | 2.86_949.6254m/z | 1.48 | 4.55E-02 | 5.47E-02 | 4.70E-02 | TG(57:11) | Glycerolipids |
| 213 | 1.54_246.2423m/z | 11.11 | 4.57E-02 | 1.32E-01 | 4.69E-02 | Myristic acid | Fatty acyls |
| 214 | 1.11_839.5398m/z | 0.47 | 4.57E-02 | 5.47E-02 | 4.68E-02 | PG(38:3) | Glycerophospholipids |
| 215 | 1.66_195.1012m/z | 1.20 | 4.80E-02 | 5.47E-02 | 4.89E-02 | 5-Hydroxytryptophol | Other |
| 216 | 1.44_281.1164m/z | 5.30 | 4.83E-02 | 1.63E-02 | 4.90E-02 | Prolylphenylalanine | Organic acids |
| 217 | 1.81_237.1482m/z | 2.29 | 4.84E-02 | 7.82E-02 | 4.88E-02 | 3-Oxododecanoic acid | Fatty acyls |
| 218 | 2.67_429.1924m/z | 2.00 | 4.84E-02 | 1.09E-01 | 4.86E-02 | 12-Ketodeoxycholic acid | Steroids |
| 219 | 1.06_228.1219m/z | 1.47 | 4.94E-02 | 7.82E-02 | 4.94E-02 | Kynurenic acid | Organic acids |

Table S5. Identified 102 Plasma metabolites from UUO vs. sham rats

| Metabolites | Metabolite class |
| --- | --- |
| Lysine | Amino acids |
| Tyrosine | Amino acids |
| Kynurenine | Amino acids |
| Dimethylglycine | Amino acids |
| Taurine | Amino acids |
| 5-Hydroxykynurenine | Amino acids |
| Glutamate | Amino acids |
| Serine | Amino acids |
| 3-Hydroxykynurenine | Amino acids |
| 5-Hydroxytryptophan | Amino acids |
| Tryptophan | Amino acids |
| Phytosphingosine | Amines |
| 5-Hydroxytryptamine | Amines |
| Aspidospermine | Amines |
| Thymidine | Amines |
| spermidine | Amines |
| Butyrate | Organic acids |
| Phenol sulphate | Organic acids |
| Uric acid | Organic acids |
| 3-Hydroxyhippuric acid | Organic acids |
| Homovanillate | Organic acids |
| Succinate | Organic acids |
| Indoxyl sulfate | Organic acids |
| Oxalic acid | Organic acids |
| Creatinine | Organic acids |
| Homovanillic acid sulfate | Organic acids |
| Glutarate | Organic acids |
| p-Cresyl sulfate | Organic acids |
| 3-Indolehydracrylic acid | Organic acids |
| Urocanate | Organic acids |
| Citrate | Organic acids |
| 2-Ketobutyric acid | Organic acids |
| Kynurenic acid | Organic acids |
| Asymmetric dimethylarginine | Other |
| Oxoamide | Other |
| phenylacetic acid | Other |
| Allantoin | Other |
| S-Adenosylhomocysteine | Other |
| p-Cresyl glucuronide | Other |
| Phenyl glucuronide | Other |
| 8-Hydroxypurine | Other |
| N2,N2-Dimethylguanosine | Other |
| Carnitine | Other |
| Acetylcarnitine | Acyl carnitines |
| Linoelaidyl carnitine | Acyl carnitines |
| Oleoylcarnitine | Acyl carnitines |
| Butenylcarnitine | Acyl carnitines |
| 3-hydroxytridecanoylcarnitine | Acyl carnitines |
| Heptadecanoyl carnitine | Acyl carnitines |
| Pentadecanoylcarnitine | Acyl carnitines |
| Hexenoylcarnitine | Acyl carnitines |
| Fumarycarnitine | Acyl carnitines |
| Linolenyl carnitine | Acyl carnitines |
| Stearoylcarnitine | Acyl carnitines |
| Decenoylcarnitine | Acyl carnitines |
| Tetracosahexaenoic acid | Fatty acyls |
| Hexadecanedioic acid | Fatty acyls |
| Octadecanamide | Fatty acyls |
| Heptadecanoic acid | Fatty acyls |
| Palmitic acid | Fatty acyls |
| Dodecanedioic acid | Fatty acyls |
| Linolenic acid | Fatty acyls |
| Decyl acetate | Fatty acyls |
| Eicosadienoic acid | Fatty acyls |
| Leukotriene A4 | Fatty acyls |
| 3-Oxooctadecanoic acid | Fatty acyls |
| Stearic acid | Fatty acyls |
| 3-hydroxypentadecanoic acid | Fatty acyls |
| Adrenic acid | Fatty acyls |
| Chenodeoxycholic acid | Steroids |
| Allocholic acid | Steroids |
| Glycocholic acid | Steroids |
| Ursocholic acid | Steroids |
| Nutriacholic acid | Steroids |
| Androsterone | Steroids |
| 3-Oxocholic acid | Steroids |
| Tauroursodeoxycholic acid | Steroids |
| Cholate | Steroids |
| Taurochenodesoxycholic acid | Steroids |
| Ursodeoxycholic acid | Steroids |
| Deoxycholic acid | Steroids |
| Glycoursodeoxycholic acid | Steroids |
| PE(40:7) | Glycerophospholipids |
| PE(32:4) | Glycerophospholipids |
| PC(36:3) | Glycerophospholipids |
| LPE(18:4) | Glycerophospholipids |
| LPE(24:1) | Glycerophospholipids |
| PC(32:1) | Glycerophospholipids |
| PE(40:6) | Glycerophospholipids |
| MG(20:3) | Glycerolipids |
| TG(52:2) | Glycerolipids |
| MG(20:5) | Glycerolipids |
| MG(22:1) | Glycerolipids |
| MG(18:3) | Glycerolipids |
| MG(22:6) | Glycerolipids |
| TG(49:4) | Glycerolipids |
| TG(58:13) | Glycerolipids |
| DG(38:7) | Glycerolipids |
| MG(16:0) | Glycerolipids |
| MG(15:0) | Glycerolipids |
| DG(44:10) | Glycerolipids |
| MG(17:0) | Glycerolipids |

Table S6. The synthesis and metabolism associated KEGG pathway of tryptophan in UUO and sham rats

| Predicted KEGG pathway | Sham | | UUO | | P |
| --- | --- | --- | --- | --- | --- |
|  | Relative abundance | SD | Relative abundance | SD |  |
| K00453; tryptophan 2,3-dioxygenase [EC:1.13.11.11] | 6.99E-06 | 7.00E-06 | 1.06E-06 | 1.06E-06 | 8.43E-02 |
| K00463; indoleamine 2,3-dioxygenase [EC:1.13.11.52] | 5.57E-10 | 5.58E-10 | 2.21E-10 | 2.22E-10 | 4.10E-01 |
| K00466; tryptophan 2-monooxygenase [EC:1.13.12.3] | 4.86E-06 | 4.86E-06 | 1.92E-06 | 1.92E-06 | 3.10E-02 |
| K01667; tryptophanase [EC:4.1.99.1] | 1.12E-04 | 1.12E-04 | 1.18E-04 | 1.18E-04 | 6.53E-01 |
| K01695; tryptophan synthase alpha chain [EC:4.2.1.20] | 4.16E-04 | 4.16E-04 | 4.24E-04 | 4.24E-04 | 6.40E-01 |
| K01696; tryptophan synthase beta chain [EC:4.2.1.20] | 6.20E-04 | 6.20E-04 | 6.35E-04 | 6.35E-04 | 6.17E-01 |
| K01867; tryptophanyl-tRNA synthetase [EC:6.1.1.2] | 8.46E-04 | 8.46E-04 | 8.64E-04 | 8.64E-04 | 4.12E-01 |
| K02846; N-methyl-L-tryptophan oxidase [EC:1.5.3.-] | 2.30E-06 | 2.30E-06 | 1.08E-06 | 1.08E-06 | 1.98E-01 |
| K03835; tryptophan-specific transport protein | 1.82E-06 | 1.82E-06 | 1.35E-06 | 1.35E-06 | 5.02E-01 |
| K03836; low affinity tryptophan permease | 1.61E-08 | 1.60E-08 | 1.40E-07 | 1.40E-07 | 3.37E-01 |
| K06285; transcription attenuation protein (tryptophan RNA-binding attenuator protein) | 1.19E-05 | 1.19E-05 | 1.50E-05 | 1.50E-05 | 1.34E-01 |
| K07185; tryptophan-rich sensory protein | 5.87E-05 | 5.87E-05 | 6.73E-05 | 6.73E-05 | 1.20E-01 |
| K16187; tryptophan synthase/phosphoribosylanthranilate isomerase [EC:4.2.1.20 5.3.1.24] | 2.39E-07 | 2.39E-07 | 3.70E-08 | 3.70E-08 | 4.55E-02 |

Table S7. The synthesis and metabolism associated-enzyme of tryptophan in UUO and sham rats

| Predicted KEGG pathway | Sham | | UUO | | P |
| --- | --- | --- | --- | --- | --- |
|  | Relative abundance | SD | Relative abundance | SD |  |
| EC:1.13.11.11 tryptophan 2,3-dioxygenase | 6.99E-06 | 7.00E-06 | 1.06E-06 | 1.06E-06 | 8.43E-02 |
| EC:1.13.11.52 indoleamine 2,3-dioxygenase | 5.57E-10 | 5.58E-10 | 2.21E-10 | 2.22E-10 | 4.10E-01 |
| EC:1.13.12.3 tryptophan 2-monooxygenase | 4.86E-06 | 4.86E-06 | 1.92E-06 | 1.92E-06 | 3.10E-02 |
| EC:1.14.13.9 kynurenine 3-monooxygenase | 3.88E-07 | 3.89E-07 | 6.84E-08 | 6.85E-08 | 7.22E-02 |
| EC:1.14.19.9 tryptophan 7-halogenase | 1.18E-05 | 1.18E-05 | 4.12E-07 | 4.12E-07 | 1.24E-01 |
| EC:2.5.1.72 quinolinate synthase | 3.49E-04 | 3.49E-04 | 3.28E-04 | 3.28E-04 | 1.33E-01 |
| EC:3.7.1.3 kynureninase | 1.18E-05 | 1.18E-05 | 3.25E-06 | 3.25E-06 | 9.14E-02 |
| EC:4.1.99.1 tryptophanase | 1.12E-04 | 1.12E-04 | 1.18E-04 | 1.18E-04 | 6.53E-01 |
| EC:4.2.1.20 tryptophan synthase | 1.23E-03 | 1.23E-03 | 1.26E-03 | 1.26E-03 | 5.11E-01 |
| EC:6.1.1.2 tryptophan---tRNA ligase | 8.46E-04 | 8.46E-04 | 8.64E-04 | 8.64E-04 | 4.12E-01 |
